# A review of Grey and academic literature of evaluation guidance relevant to public health interventions

**DOI:** 10.1186/s12913-017-2588-2

**Published:** 2017-09-12

**Authors:** Sarah Denford, Charles Abraham, Margaret Callaghan, Peter Aighton, Frank De Vocht, Steven Arris

**Affiliations:** 1 Psychology Applied to Health group, Institute of Health Research 2.09 College house, St Lukes Campus, University of Exeter Medical School, Exeter, EX1 2LU UK; 2 School of Social and Community Medicine, University of Bristol, Bristol, UK; 3 University of Sheffield, Sheffield, UK

**Keywords:** Evaluation guidance, Public health, Review

## Abstract

**Background:**

Public Health evaluation is essential to understanding what does and does not work, and robust demonstration of effectiveness may be crucial to securing future funding. Despite this, programs are often implemented with poor, incomplete or no evaluation. Public health practitioners are frequently required to provide evidence for the effectiveness of their services; thus, there is a growing need for evaluation guidance on how to evaluate public health programs. The aim of this study is to identify accessible high-quality, evaluation guidance, available to researchers and practitioners and to catalogue, summarise and categorise the content of a subset of accessible, quality guides to evaluation.

**Methods:**

We systematically reviewed grey and academic literature for documents providing support for evaluation of complex health interventions. Searches were conducted January to March 2015, and included academic databases, internet search engines, and consultations with academic and practicing public health experts. Data were extracted by two authors and sent to the authors of the guidance documents for comments.

**Results:**

Our initial search identified 402 unique documents that were screened to identify those that were (1) developed by or for a national or international organization (2) freely available to all (3) published during or after 2000 (4) specific to public health. This yielded 98 documents from 43 organisations. Of these, 48 were reviewed in detail. This generated a detailed catalogue of quality evaluation guidance. The content included in documents covers 37 facets of evaluation.

**Conclusions:**

A wide range of guidance on evaluation of public health initiatives is available. Time and knowledge constraints may mean that busy practitioners find it challenging to access the most, up-to-date, relevant and useful guidance. This review presents links to and reviews of 48 quality guides to evaluation as well as categorising their content. This facilitates quick and each access to multiple selected sources of specific guidance.

**Electronic supplementary material:**

The online version of this article (10.1186/s12913-017-2588-2) contains supplementary material, which is available to authorized users.

## Background

Evaluation is foundational to identification, implementation and dissemination of effective and cost-effective interventions. There have been many calls to ensure and improve evaluation of interventions and initiatives designed to improve public health. For example, the US Centre for Disease Control and Prevention note that in order to improve the health of the public, “*we must devote our skill - and our will – to evaluating the effects of public health actions*” [1]. In the UK, the 2011 House of Lords report on Behaviour change recommend that “*a lot more could and should, be done to improve the evaluation of interventions*” [2] and a study by the EPPI-centre at the University of London led Public Health England to call for improved evaluation; stating that “*whilst interventions are being commissioned by a variety of organisations, data informing the relative ‘success’ of the interventions, in terms of the intended health outcomes, was patchy and inconsistent”* [3]. Evaluation is essential to understanding what does and does not work and robust demonstration of effectiveness may be crucial to securing future funding.

Evaluation of public health interventions may be complex and expensive, in part, because such interventions are themselves complex [4]. Public health interventions may, for example, attempt to engage and multiple individuals, organisations, and / or communities and target changes in knowledge, attitudes, and practices at multiple levels over long periods of time. They also operate in complex demographic and socio-economic contexts [4]. These “real life” complexities render evaluation challenging. Nonetheless, when financial resources are scarce, it is crucial that evidence of effectiveness directs selection of interventions. Repeatedly funding ineffective programs not only squanders valuable resources but has opportunity costs resulting from failure to implement potentially beneficial interventions. Without good evaluation, it is impossible to distinguish between programs that are having a substantial health impact, those that need to be adapted for different populations, those that need to be withdrawn, and those that may be harmful [5]. Despite this, programs are often implemented with poor, incomplete or no evaluation.

Evaluability assessment facilitates decisions by practitioners, commissioners and researchers about what programs most need to be evaluated. Ogilvie et al. identify five key questions that should be answered before investing in an evaluation [6]. These focus on the stage of development or intervention implementation, whether or not the results of the evaluation are likely to lead to changes in policy or practice, how widespread or important effects of an intervention are likely to be (i.e., is it likely to have a large effect on a large number of people), and how will findings of the evaluation contribute to existing evidence? Such guidance enables evaluation priorities to be identified when funds for evaluations are limited.

When evaluation is undertaken it is vital that it is conducted in a manner that will produce robust answers to the questions addressed. This requires expertise and, not all practitioners may have had adequate training to enable them to undertake evaluations without support. Consequently, public health practitioners and commissioners may feel under-skilled to conduct evaluations [7]. This highlights the need for high-quality, useable, feely available and practical evaluation guidance on how to evaluate public health programs.

Appeals for better evaluation practice led to a proliferation of guidance and advice to support evaluation of public health programs [4, 6]. For example, the US Centres for Disease Control and Prevention (CDC) developed a framework to guide public health professionals perform evaluations. The framework includes a series of steps; from engaging stakeholders through to dissemination of findings. Many of these activities are part of routine practice but others are not. This framework includes a series of standards that are intended to ensure that evaluations are well designed, rigorous, and suitable for purpose. Different guidance is provided by the revised UK Medical Research Council (MRC) framework [4], aimed mainly at academic researchers. This provides an overview of the phases and processes involved in the development, implementation and evaluation of complex interventions and, in contrast, to an earlier MRC framework [4], this revised guide provides advice on how to evaluate highly complex programs using a variety of methods – not just controlled clinical trials. These are just two of many guides to public health evaluation (see too, for example, [1, 8–10].

Such guidance has the potential to facilitate the quality of evaluations and increase the number of programs with strong evidence for their effectiveness which, in turn would allow withdrawal of programs that are ineffective or lack evidence of effectiveness. However, to be useful such guidance has to be accessible to practitioners and commissioners; they need to know which guides to evaluation to use for what purpose. Unfortunately, the many different guides available may be a barrier to identification of relevant guidance. Novice evaluators who turn to the internet for guidance are faced with many choices and have no map describing the content of available evaluation and so may find it difficult to know what guidance to follow. Some guides provide generic advice on evaluation generally, others on particular types of evaluation (e.g., process or economic evaluation). Some documents are written for academics, or policy makers, or funders, or experienced evaluators. Other guides are topic specific; such as the UK Public Health England frameworks for obesity prevention. This can be overwhelming for novice evaluators who have no easy way to select appropriate high-quality guidance for particular evaluation projects [7]. This lack of guidance on how to access and use guidance on public health guidance is clear from conversations with practitioners. For example, in a qualitative study of UK public health practitioners’ views of evaluation on practitioner commented, “*If I were to begin evaluation tomorrow and I did a search on evaluation I’d probably… come up with about 500 hits - but actually they aren’t all equal and some are more appropriate than others and understanding which is the best one to use would be difficult for me. So some advice about quality and/or types of tools or particular types of evaluation would be helpful*”.

### The present study

We aimed to review this literature in order to assess the extent of available guides to evaluation relevant to public health interventions and to identify the content of such guidance. We also planned to provide a catalogue of high-quality, readily-accessible guides that would help practitioners navigate this literature. It was not our intention to synthesis this information, merely describe what is in the documents, and to provide a signpost to inform practitioners where information can be found. We had two specific aims.

### Specific aims

1. To identify accessible high-quality, evaluation guidance, available to public health researchers and practitioners
2. To summarise and categorise the content of a subset of accessible, quality guides to evaluation.

## Methods

### Search strategy

Evaluation guidance documents were identified between January and March 2015 using five strategies: (1) searching electronic databases (2) hand searching of identified guides and journals (3) searching internet resources (4) citation searching and (5) contacting key authors and professionals in the field.

EMBASE, MEDLINE, MEDLINE-in-process, Health management information consortium (HMIC), Social Policy and Practice (SSP), Web of Science, and PsycINFO were searched using the search strategy presented in Additional file 1. Reference lists of identified papers, and key journals were also scrutinised.

It was anticipated that a substantial proportion of guidance documents would not take the form of academic papers and so would not be identified using a traditional literature search. To identify as many non-academic documents as possible, we searched the four main internet search engines (Google, BING, Yahoo, WebCrawler) using a modification of the search strategy (Additional file 1). The first 30 pages that were retrieved from each database using each term were screened. We then searched a series of health and evaluation websites (Additional file 1) using the term “evaluation.” Websites were identified through discussions with academics and public health professionals. Finally, key authors and experts in the field were asked to suggest documents/ websites/ policies that we missed.

The search was conducted by two authors (author one and three) with guidance and support from author 2. We included websites, books, journal articles, policy recommendations, educational resources, tools and frameworks that provide support to public health practitioners undertaking evaluations of public health interventions.

We excluded all documents and articles in which the aim was to evaluate a specific intervention (as opposed to offering advice on how to undertake evaluations). Papers reporting on the development of questionnaires or assessment scales for specific interventions were also excluded.

### Inclusion criteria

We included all documents, websites, books, journal articles, policy recommendations, educational resources, tools and frameworks that provide support to public health practitioners reviewing or undertaking evaluations of public health interventions. Documents may be:

- Resources supporting the conduct of evaluations or an aspect of evaluation (including monitoring an intervention, collaboration, implementation and dissemination);
- Principles that practitioners should follow when conducting evaluations (including economic and process evaluations)
- Resources that help practitioners decide how and when to evaluate interventions
- Standards of good or best practice;
- Recourses supporting identification of outcome indicators
- Resources to help practitioners assess quality of evidence / principles of effectiveness
- Resources to help practitioners identify useful interventions
- Resources to help support practitioners make informed decisions about whether an intervention is likely to be effective in their practice

Documents and articles in which the aim is to evaluate a specific intervention (as opposed to informing others how to evaluate) were excluded. Development of questionnaires or assessment scales for specific interventions were also excluded.

### Selecting a subset of evaluation guidance documents

Our search identified 402 guidance documents that can be used to support the evaluation of public health programs. This list included books, reports, webpages, and academic articles. These documents had a range of aims including: (i) elucidating the principles that practitioners should follow when conducting evaluations, (ii) specifying standards of good evaluation practice, (iii) offering advice on how and when to undertake evaluations, (iv) offering instruction on particular evaluation approach e.g., economic or process evaluations, (v) providing online support and advice to practitioners, including tools to support evaluation.

From this list of 402 documents, we selected a subset to review and catalogue in detail. Through discussion the authors agreed four selection criteria. First, that documents were free and readily available to public health practitioners. Second, given the changing nature of public health practice, we focused on documents written in or after the year 2000. Third, to provide a quality indicator we selected documents sourced or created by national or international organisations. Fourth, and finally, we assessed the relevance of each document to public health.

This resulted in a reduced list of 98 documents produced by 25 organisations. We then reviewed the documents produced be each of these organisations and selected the most comprehensive or recent evaluation guidance, resulting in 25 guides. A further 23 guides were added because the authors agreed that documents provided by the US Centers for Disease Control and Prevention, the UK Medical Research Council, the UK National Health Service, and Public Health England were complementary to the initial selection of 25 and judged to be equally valuable to practitioners. So, for these four organisations, more than one guide to evaluation was retained for detailed examination. A flow diagram of this process is presented in Additional file 2: Fig. S1. The final list of these 48 evaluation guidance documents is provided in Additional file 3.

### Summarising the content of selected guides to evaluation

Each of the 48 guides were read by two authors. A content template was developed through discussion and short one-page summaries of each of the 48 guides were produced independently by each reviewer. Each summary provided information on the target audience of the guide, its main aim, a short overview of the guide, and strengths and limitations. Links to the resource and associated resources were also included. The two reviews of each guide were then combined, retaining content from each review and resolving any discrepancies through discussion. The final (integrated) review was then sent to the original author of the document for verification. Authors were asked if they (i) considered the summary to be an accurate and good reflection of their document (ii) if anything was missing and (iii) if they thought this would be useful to practitioners.

### Content categorisation of evaluation guidance documents

Once summaries had been completed, two authors independently coded the content of the 48 guides. Initially, a selection of 5 documents were read by each reviewer and their content listed. Content lists were developed based on what was in documents, rather than according to a pre-existing checklist. Merging of these two content lists through discussion resulted in a set of 44 content categories. The two authors then jointly coded a further 5 documents, and discussed and refined the list accordingly. The final list contained 37 content categories generated by the initial two-stage coding of 10 guides; which was then used to categorise the content of the remaining 38 guides.

## Results

### Overview of 98 guides to evaluation

While we identified 402 guides to evaluation only 98 were relevant to public health, free and readily available and produced since 2000 by a national or international organisation. These 98 varied in terms of purpose, topic or condition, and audience. The large majority were general overviews of evaluation focusing on principles of evaluation and how to assess evidence to support evaluation. A variety of evaluations were considered including, trials, naturally-occurring experiments, process evaluations and economic evaluations and various advice and instructions were provided on how to plan prepare for and conduct such evaluations. The guides were mainly generic but some focused specifically on evaluating international development, obesity, asthma, sexual health, mental health and physical activity. One focused on children and families, one on healthy eating and one on drugs and one on violence on women and girls. However, even condition-specific guides provided instruction and support that relevant to evaluations of other types of interventions. For example, some guides, focused on evaluating interventions relevant to particular health problem (asthma, smoking and obesity) to illustrate more general lessons. Documents ranged from targeting those with no knowledge of evaluation, to sophisticated guides for those experienced in evaluation practice with target audiences including novices, evaluation experts, program managers, health care professionals, government officials and academics/researchers.

### Response from authors

Of the 48 documents we reviewed, copies of our review were sent to the lead authors of each guide. If we were unable to find contact details for the main author, or there was no named author, we contacted the chair of a research group or a general enquiries email. In some cases contacts passed us on to someone else. We sent reminders up to three times, in some cases trying alternative addresses. Fourteen authors did not respond. Two organisations (representing a total of seven documents) sent standard replies stating that they did not respond to such requests. Twenty seven authors replied to state that they were happy with the summary or to suggest minor changes.

### Content categorisation

Merging categories identified by two researchers independently coding the content of two sets of five guides resulted in a list of 37 content categories that were used to describe the content of 48 selected guides. These categories were grouped into 1) Background to evaluation 2) Pre-evaluation preparatory work 3) The Evaluation process 4) Evaluation approaches and 5) Additional support. Tables 1, 2, 3, 4 and 5 list content categories within each of these five groupings. The tables also lists each of the 48 documents that contained content corresponding to each of the 37 categories. Thus these tables can be used by readers to find guides (among the 48) with particular content. These tables can be used in conjunction with the 48 brief summaries of these guides which are provided in Additional file 4, and the Tables summarising the catagorisation in Additional file 5. Each of the 48 guides was been given a brief title that is listed below and used both in Tables 1, 2, 3, 4 and 5 and in Additional file 4.

**Table 1:** Background to evaluation

Content category	Guidance
Overview of evaluation	APCRC
	Capacity for health
	DFID Evaluation guide
	EMCDDA Evaluation Resource Kit
	Food standards agency
	First Nations Evaluation
	Health Scotland Mental Health Improvement
	JRF Community Evaluation
	Magenta Book
	NSF Project Evaluation
	PHE1 Introduction
	PHE3 Dietary interventions
	PHE4 Physical Activity
	PHE5 Weight management interventions
	UNW
	USDHH2 Evaluation
	WK Kellogg
	WHO2
Assessing the evidence	AHRQ
	APCRC
	ECDPC Assessing evidence
	EMCDDA Evaluation Resource Kit
	The Green Book
	Health Scotland Mental Health Improvement
	Magenta Book
	MRC1 Framework
Evidence based medicine	ECDPC Assessing evidence
	EMCDDA Evaluation Resource Kit
	Health Scotland Mental Health Improvement
	MRC1 Framework
Evaluability	Better Evaluation
	Evaluability Assessment
	NHS Scotland
Common Challenges	Health Scotland Mental Health Improvement
	UNDP
	UNEG
	UNW
	USDHH2 Evaluation
Policy and Evaluation	AHRQ
	DFID Evaluation Guide
	The Green Book
	Magenta Book
Using theory in Evaluation	APCRC
	LEAP
	MRC1 Framework
	MRC2 Process evaluation
	NSF Project Evaluation
	UKES
	UNW
	USDHH2 Evaluation
	The World Bank

**Table 2:** Pre-evaluation preparatory work

Content category	Guidance
Developing a protocol	APCRC
	Better Evaluation
	MRC2 Process Evaluation
Budgeting	Better Evaluation
	CDC3 Evaluation Guide
	Charities Evaluation Service
	Evaluation support Scotland
	Magenta Book
	The green book
	MRC1 Framework
	MRC2 Process evaluation
	MRC3 natural experiments
	NSF Project evaluation
	LEAP
	USDHH1 Cost Effectiveness
	USDHH2 Evaluation
	WK Kellogg
	WHO2
Contracting and communications	Better Evaluation
	CDC2 Evaluation planning
	CDC3 Evaluation Guide
	CDC4 Implementing Evaluations
	DFID evaluation guide
	The green book
	Magenta Book
	LEAP
	UNEG
	UNDP
	USDHH2 Evaluation
	WK Kellogg
	WHO1
Pilot testing	CDC4 Implementing evaluations
	The Green Book
	Magenta Book
	MRC1 Framework
	MRC2 Process evaluation
Ethics	Better Evaluation
	ECDPC Assessing evidence
	Food Standards Agency
	Magenta Book
	PHE1 Introduction
	PHE3 Dietary interventions
	PHE4 Physical Activity
	PHE5 Weight management interventions
	UNEG
	WHO1
Needs assessment	Better Evaluation
	Capacity for health
	First Nations Evaluation
	LEAP
	PHE1 Introduction
	PHE3 Dietary interventions
	PHE4 Physical Activity
	PHE5 Weight management interventions
	UNDP
	UNW
	USDHH2 Evaluation
	UNAIDS
	WHO2 Evaluation
Evaluation planning	APCRC
	Better Evaluation
	Capacity for health
	CDC2 evaluation plan
	CDC3 Evaluation Guide
	CDC4 Implementing Evaluation
	Charities Evaluation Service
	DFID Evaluation Guide
	First Nations Evaluation
	Magenta book
	MRC2 Process Evaluation
	NSF Project Evaluation
	LEAP
	PHE1 Introduction
	PHE3 Dietary interventions
	PHE4 Physical Activity
	PHE5 Weight management interventions
	UNDP
	UNW
	USDHH2 Evaluation
	WHO2 Evaluation
Logic modelling	Better Evaluation
	Capacity for health
	CDC2 Evaluation Plan
	Charities Evaluation Service
	Evaluation support Scotland
	First Nations Evaluation
	Magenta Book
	MRC1 Framework
	MRC2 Process Evaluation
	NSF Project Evaluation
	NHS Scotland
	PHE1 Introduction
	PHE3 Dietary interventions
	PHE4 Physical Activity
	PHE5 Weight management interventions
	UNW
	USDHH2 Evaluation
	WK Kellogg
	The World Bank
	WHO2 Evaluation
Stakeholder involvement	APCRC
	Better Evaluation
	CDC1 Framework
	CDC2 Evaluation Plan
	CDC3 Evaluation Guide
	CDC4 Implementing Evaluation
	ECDPC Assessing evidence
	DFID Evaluation Guide
	The Green Book
	LEAP
	Magenta Book
	MRC2 Process Evaluation
	NSF Project Evaluation
	UNDP
	UNAIDS
	WK Kellogg
	WHO2

**Table 3:** Evaluation Processes

Content category	Guidance
Overview of evaluation process	APCRC
	CDC1 Evaluation Framework
	CDC2 Evaluation plan
	CDC3 Evaluation guide
	CDC4 implementing evaluation
	CDC5 Process evaluation
	CDC6 Evaluation Resources
	Magenta Book
	DFID Evaluation guide
	First Nations Evaluation
	NSF Project Evaluation
	PHE1 Introduction
	PHE3 Dietary interventions
	PHE4 Physical Activity
	PHE5 Weight management interventions
	UNDP
	USDHH2 Evaluation
	WK Kellogg
Defining questions	APCRC
	Better Evaluation CDC2 Evaluation plan
	CDC3 Evaluation guide
	CDC4 Implementing Evaluations
	Evaluation Support Scotland
	First Nations Evaluation
	LEAP
	MRC1 Framework
	NSF Project Evaluation
	PHE1 Introduction
	PHE3 Dietary interventions
	PHE4 Physical Activity
	PHE5 Weight management interventions
	UNDP
	USDHH1 Cost effectiveness
	USDHH2 Evaluation
	WK Kellogg
	WHO2
Choosing outcomes	Better Evaluation
	CDC2 Evaluation plan CDC3 Evaluation guide
	CDC4 Implementing evaluation
	Charities Evaluation Service
	Evaluation Support Scotland
	First Nations Evaluation
	LEAP
	MRC1 Framework
	MRC2 Process evaluation
	MRC3 Natural experiments
	NSF Project Evaluation
	NHS Scotland
	PHE1 Introduction
	PHE3 Dietary interventions
	PHE4 Physical Activity
	PHE5 Weight management interventions
	UNDP
	UNW
	USDHH1 Cost effectiveness
	USDHH2 Evaluation
	Wellbeing Evaluation Tools
	The World Bank
	WHO2
Describing the intervention	Better Evaluation
	CDC1 Framework
	CDC2 Evaluation plan
	CDC3 evaluation guide
	First Nations Evaluation
	LEAP
	MRC1 Framework
	MRC2 Process evaluation
	MRC3 natural experiments
	NSF Project Evaluation
	PHE1 Introduction
	PHE3 Dietary interventions
	PHE4 Physical Activity
	PHE5 Weight management interventions
	UNDP
	UNW
	USDHH1 Cost effectiveness
	USDHH2 Evaluation
	WHO2
Research Design and methods	APCRC
	Better Evaluation
	CDC1 Evaluation Framework
	CDC2 Evaluation plan
	CDC3 Evaluation guide
	CDC4 implementing evaluations
	Charities Evaluation Service
	DFID Evaluation Guide
	Evaluation Support Scotland
	Food Standards Agency
	LEAP
	Magenta Book
	MRC1 Framework
	MRC2 Process Evaluation
	MRC3 Natural Experiments
	NSF Project Evaluation
	Treasury board Canada
	UNEG
	UNDP
	UNW
	USDHH1 Cost effectiveness
	USDHH2 Evaluation
	Wellbeing evaluation tools
	The World Bank
	WK Kellogg
	WHO2
Collecting data	Better Evaluation
	Capacity for Health
	CDC1 Framework
	CDC2 Evaluation plan
	CDC3 Evaluation guide
	CDC4 Implementing evaluations
	Charities Evaluation Service
	DFID Evaluation guide
	Evaluation Support Scotland
	Food Standards Agency
	First Nations Evaluation
	LEAP
	Magenta Book
	MRC3 Natural Experiments
	NSF Project Evaluation
	PHE1 Introduction
	PHE3 Dietary interventions
	PHE4 Physical Activity
	PHE5 Weight management interventions
	Treasury Board Canada
	UNDP
	UNW
	USDHH1 Cost Effectiveness
	USDHH2 Evaluation
	The World Bank
	WK Kellogg
	WHO2
Managing, analysing and interpreting data	Better Evaluation
	Capacity for Health
	CDC1 Framework
	CDC3 Evaluation plan
	DFID Evaluation guide
	Evaluation Support Scotland
	Food Standards Agency
	MRC1 Framework
	MRC2 Process Evaluation
	MRC3 natural experiments
	NSF Project Evaluation
	PHE1 Introduction
	PHE3 Dietary interventions
	PHE4 Physical Activity
	PHE5 Weight management interventions
	Treasury Board Canada
	UNDP
	UNW
	USDHH1 Cost Effectiveness
	USDHH2 Evaluation
	The World Bank
	WK Kellogg
	WHO2
Learning and reporting	Better Evaluation
	Capacity for Health
	CDC1 Framework
	CDC2 evaluation plan
	CDC3 evaluation guide
	CDC4 implementing evaluation
	DFID Evaluation guide
	Evaluation Support Scotland
	Food Standards Agency
	JRF Community evaluation
	Magenta book
	MRC1 Framework
	NSF Project Evaluation
	PHE1 Introduction
	PHE3 Dietary interventions
	PHE4 Physical Activity
	PHE5 Weight management interventions
	UNDP
	UNW
	USDHH1 Cost effectiveness
	USDHH2 Evaluation
	WK Kellogg
	WHO2

**Table 4:** Types of evaluation

Overview of types of evaluation	APCRC
	Better Evaluation
	NSF Project Evaluation
	NHS Scotland
	WK Kelloggs
Process evaluation	Better Evaluation
	CDC5 Process evaluation
	DFID evaluation guide
	Food Standards Agency
	Magenta book
	MRC1 Framework
	MRC2 process evaluation
	NHS Scotland
	PHE1 Introduction
	PHE3 Dietary interventions
	PHE4 Physical Activity
	PHE5 Weight management interventions
	UNAIDS
	WK Kelloggs
Outcome evaluation	DFID evaluation guide
	LEAP
	Magenta book
	MRC1 framework
	MRC2 process evaluation
	NHS Scotland
	PHE1 Introduction
	PHE3 Dietary interventions
	PHE4 Physical Activity
	PHE5 Weight management interventions
	UNDP
	UNW
	UNAIDS
	The World Bank
	WK Kelloggs
Economic evaluation	CDC2 evaluation plan
	DFID evaluation guide
	The Green book
	Magenta book
	MRC1 Framework
	USDHH1 Cost effectiveness
	The World Bank
	WHO1
Natural experiments	MRC3 Natural experiments
Community projects	First Nations Evaluation
	LEAP
Fidelity	MRC1 Framework
	MRC2 Process Evaluation
	PHE1 Introduction
	PHE3 Dietary interventions
	PHE4 Physical Activity
	PHE5 Weight management interventions
	UNAIDS
	WK Kelloggs

**Table 5:** Additional support

Organisations offering support	Better Evaluation
	CDC6 Evaluation resources
	Charities Evaluation Service
	LEAP
	UKES
Recommendations	NIHR
	UNEG
	UNDP
	UNICEF
Tools and toolkits	APCRC
	Better Evaluation
	CDC2 evaluation plan
	CDC3 evaluation guide
	CDC4 implementing evaluation
	DFID Evaluation Guide
	ECDPC Assessing Evidence
	EMCDDA Evaluation Resource Kit
	Evaluation Support Scotland
	Food Standards Agency
	Health Scotland Mental Health Improvement
	JRF Community Evaluation
	NHS Scotland
	PHE1 Introduction
	PHE3 Dietary interventions
	PHE4 Physical Activity
	PHE5 Weight management interventions
	Treasury Board Canada
	USDHH2 Evaluation
	Well-being Evaluation Tools
	WHO2
Links	Better Evaluation
	Capacity for Health
	CDC1 framework
	CDC2 Evaluation plans
	CDC6 Evaluation resources
	EES
	Evaluation Support Scotland
	LEAP
	The World Bank
Quality assurance	APCRC
	Capacity for Health
	CDC1 Framework
	CDC2 Evaluation Plan
	CDC3 Evaluation guide
	UNDP
Hiring and evaluator	CDC3 Evaluation guide
	Evaluation Support Scotland
	Food Standards Agency
	NSF Project Evaluation
	USDHH2 Evaluation
	WK Kellogg
Training	APCRC
	Better Evaluation
	CDC3 Evaluation Guide
	CDC4 Implementing evaluation
	Charities Evaluation Service
	European Evaluation Society
	Evaluation Support Scotland
	LEAP
	PHE2 Resources
	UKES
	UNEG

#### Background to evaluation

Seven content categories were grouped as “background to evaluation” (see Table 1). These were: Evaluation overview; assessing the evidence; evidence based practice; evaluability; common evaluation challenges; policy and evaluation; and using theory in evaluation.

Eighteen guides provided an overview of evaluation. This included explanation of the nature of evaluation and how it differs from other types of research; why evaluation is needed and what it can tell us; and the benefits of conducting evaluation. These documents usually targeted practitioners who were new to evaluation.

Eight guides informed readers how to identify and assess the quality and relevance of existing research and evaluations. This frequently included links to quality assessment scales such as CONSORT and the Equator network. Four of these eight guides also discuss evidence based practice (EBP); what EBP is, why it is important, and how to conduct evaluations within an evidence based framework. The importance of choosing “best available” methods, even if they are not optimum, are highlighted.

Theory is a critical part of intervention development. Theory driven evaluation aims to examine hypothesised causal processes. Nine guides discuss the use of theory in program development and evaluation and two UK Medical Research Council documents (MRC1 Framework and MRC2 Process evaluation- see below) were found to be particularly useful in this regard.

Challenges inherent in evaluation of public health initiatives are numerous. It is not always possible or practical to conduct a high-quality evaluation, and five documents present sections on methodological and practical challenges and ways of overcoming such challenges. Just one document (Evaluability Assessment) focused on evaluability; which is defined as “*the extent to which an activity or project can be evaluated in a reliable and credible fashion.” T*he authors review the literature on evaluability, and provide an overview of what it is, its purpose, what it includes, and how it can be completed.

Four guides (including the UK Government Magenta book and The Green book) focus on the evaluation of policy. These books are intended to be used by policy makers working in or with the UK government in order to support evidence for policy making.

#### Pre-evaluation work

Nine content categories were used to identify information on the steps prior to launching an evaluation (see Table 2). This included: completing a needs assessment; developing a logic model; planning the evaluation; developing a protocol; budgeting; developing contracts and establishing communications; pilot testing; obtaining ethical approval; and involving stakeholders.

Thirteen guides provide information and resources to support needs assessments; including how to collect data about a population or community to inform the intervention, how to identify issues and problems, how to assess whether or not issues and problems are shared by the target population, and how to assess populations or communities at the start of an intervention or program. A needs assessment may feed into the development of a logic model. These are diagrammatic representations of the program, describing delivery mechanisms, intervention components, mechanisms of impact, and intended outcomes. Logic models were discussed in 20 guides. A logic model is part of the evaluation plan; a written document specifying the direction the evaluation should take based on priorities, resources, time, and skills needed to complete the evaluation. These guides recommend that all stakeholders should be involved in the development of such a plan to ensure that the process is clear, and to establish consensus on the purpose and procedures of the evaluation. Twenty one guides discuss the process of developing an evaluation plan; including one document by the CDC which focuses exclusively on planning (CDC2 Evaluation Plan). Only three documents specifically discussed the processes involved in the development of an evaluation protocol (Better Evaluation, MRC2 Process Evaluation and APCRC). Of particular note is the UK NHS document (APCRC) in which a protocol template is provided.

Once developed, guides recommend that evaluation methods, materials, and procedures should be piloted testing for feasibility. It is important to know, for example, if it is possible to recruit participants, if the data collection tools are suitable, if the outcomes measured are appropriate. Five guides discuss pilot testing. Evaluation can be costly and when designing an evaluation, the questions asked and methods chosen must reflect the funds available. Fifteen guides provide information and support on how to budget for an evaluation. A range of ethical issues need also be considered when planning an evaluation of public health initiatives. For example, in some cases, it may not be ethical to withhold an intervention from a group of people. In such cases, randomised controlled trials would not be suitable. There are also ethical issues surrounding informed consent and data collection, and issues surrounding health inequalities. Ten guides discuss such ethical issues.

Seventeen guides discuss the importance of stakeholder involvement, or strategies for involving stakeholders in the evaluation process. Many of these documents also provide advice about facilitating healthy communications between stakeholders, or developing evaluation contracts so that each party has a clearly specified role.

#### Evaluation processes and procedures

Eight content categories relate to the processes and procedures of completing an evaluation (see Table 3). Eighteen guides include an overview of the processes involved in evaluation. This frequently took the form of a checklist of activities involved in evaluation. In some instances, the checklist was structured in terms of essential and desirable features. Other evaluation process content categories include defining an evaluation question; specifying outcomes; describing the intervention; choosing research design and methods; collecting data; managing, analysing and interpreting data; and learning and reporting.

A total of 19 guides provide information on developing a research question. Such documents highlight the need to choose a question that can be answered within the confines of the time, resources, and skill sets that are available; and note the importance of designing a question which is important and useful to all key stakeholders, and can feasibly be answered. Defining the research question is intricately linked to understanding the program, and understanding the outcome. Specifically, how to describe the goal of the program, any activities, and what is and is not part of the program, and how to choose, define, and develop outcomes and outcome measures. Nineteen guides include a section describing how programs should be described, and 24 guides discuss factors including what makes a good indicator, and suggest a number of considerations when selecting indicators. In some cases outcome indicators or measures are suggested, or objective or validated measures provided.

Twenty six guides discuss different research designs and methodological approaches that may be used when evaluating a program. This includes describing qualitative and quantitative methods, as well as discussing particular trial designs (e.g., randomised controlled trials). The majority of these documents discussed data collection approaches (i.e., observations, surveys, focus groups, interviews, existing records etc), and how to manage, analyse and interpret data. They also discuss the importance of methodological rigor, cost effectiveness and validity, reliability and credibility. However, the level of detail provided was limited. Often, guides include a brief overview of types of analyses, or the importance of matching the type of analyses to the study design and research question. Statistical advice was not provided – although links to statistics books were frequently included. Twenty two documents discuss how to learn from and disseminate findings once data has been analysed.

#### Evaluation approaches

Seven content categories were used to identify guidance on evaluation approaches (see Table 4). Five documents provide a brief overview of different approaches (i.e., process evaluation, outcome evaluation, economic evaluation). These documents briefly compare and contrast these forms of evaluation, but do not provide any real detail. Other documents either focus exclusively or in part on specific approaches including; process evaluation; outcome evaluation; economic evaluation; natural experiments; community projects; and fidelity.

Fourteen guides discuss process evaluations, and especially, the UK Medical Research Council (UK MRC) document, dedicated entirely to the conduct of process evaluation (MRC2 Process Evaluation). Fifteen guides discuss outcome evaluations in terms of what they are and how they differ to other approaches, and eight guides include a discussion of economic evaluation. This includes two documents by Gov.UK and the World Health Organisation (The Green Book and WHO2) which focus exclusively on economic evaluation. Other documents provide a definition and explanation of economic evaluations, and discuss the importance of considering cost effectiveness of programs. A separate UK MRC document (MRC3) provides detailed consideration of evaluating natural experiments, and two documents were developed specifically to aid the evaluation of community projects. These two documents note the challenges associated with community projects, and provide suggestions for overcoming such problems. Eight guides focus on fidelity, specify the importance of assessing fidelity, and provide suggestions regarding how it may be achieved.

#### Additional support

Six content categories were used to identify guidance on supporting the conduct of evaluations (see Table 5). For example, one of the reviewed guides was a website (UKES) offering support and forum for communication for all involved in evaluation. Four guides provide recommendations for achieving high quality evaluations. For example, the National Institute for Health Care and Excellence (NICE) lists a number of recommendations for evaluation. Twenty one guides include tools or tool kits to support the evaluation process. Frequently, tools were included as an appendix, and include tools such as checklists, templates, outcome indicators and surveys. Nine guides provide links to other resources in which further information is detailed. Six documents include information and support to ensure evaluations are of the highest quality. For example, the Centres for Disease Control and Prevention (CDC) framework (CDC1) include a series of standards that evaluations should adhere to. Six documents discussed factors to consider when hiring an external evaluator to complete the evaluation. Finally, 11 guides provide either links to training courses, or online training in evaluation.

## Discussion

We have presented findings from a systematic and comprehensive search of documents providing guidance on evaluation of public health initiatives. We identified 402 guides on a range of topics created for a variety of different types of users with different initial expertise. In order to render this literature more accessible we have suggested a series of five criteria that reduced our list to 98 guides. Then by selecting non-overlapping guides from national and international organisations to just 48. We have provided a brief summary of each of these guides and categorised the content of each across 37 categories. We believe that this will make the evaluation guidance literature much more accessible for public health practitioners and commissioners.

Despite the abundance of evaluation guidance, many practitioners claim that they do not use guidance documents and do not find them useful [7]. This may be in part due to an inability to easily understand the purpose, content and target audience of available guides. For example, a simple guide may not be of much use to an experienced evaluator, but could be ideal for a novice. Whilst generic guides may be used to support evaluators in any situation, they may also be lacking the necessary detail to support specific activities such as choosing outcomes. The complexity of public health evaluation makes it impossible to develop a guide that suits all needs. Our project was to begin to map out what guidance is available for whom and to provide a guide to a limited range of easily-accessible, quality-assured guidance to evaluation.

Interviews with practitioners revealed the need for quality assured, practical guidance that relates to the real-world settings in which they operate [7]. Our catalogue provides the first step in supporting practitioners conduct high quality evaluations. The next step would be to examine the utility of the guide to guidance, and of the use of the guides included. Obtaining feedback on the use of the guide to guidance will allow us to identify areas of evaluation that existing guidelines do not cover, or are not useful in their current form. Additional guidance or training resources could then be developed; however, this would require significant input from those who will be using the information.

### Strengths and limitations

We have provided researchers and practitioners with a tool to identify and use relevant evaluation guidance documents. We will have undoubtedly missed some guidance in this wide-ranging and desperate literature. However, every attempt was been made to be as inclusive and transparent as possible and we are confident that the sample selected is representative of the literature as a whole. Moreover the list of 48 reviewed and categorised guides are recent, accessible and high-quality. Our categorisation of the content of these guides will allow readers to identify guides that provide relevant, high quality information.

A second limitation of our review is the reliance on content coding to identify the 37 content categories. The categorization could have been strengthened by using evaluation resources that outline all the key aspects of evaluation practice. However, we included a large and disparate body of evaluation literature, and we were unable to identify an existing template that was inclusive enough to accommodate the depth of information presented in this literature. Using content coding, we could be confident that all aspects of evaluation included in the guidance documents were adequately reflected in the resultant framework.

To promote accuracy, the content of the guides, and the associated reviews were checked and agreed by two authors and the authors of the original guidance documents whenever possible.

Evaluation methods, tools and approaches are continuously developing and progressing. Consequently, our review will need to be updated on a regular basis. This would be relatively easy given the systematic and transparent search strategies.

## Conclusion

A wide range of guidance on evaluation of public health initiatives is available. However, time and knowledge constraints may mean that busy practitioners find it challenging to review the range of available guidance and access the most, up-to-date, relevant and useful guidance. This review presents links to and reviews of 48 quality guides to evaluation as well as categorising their content. This facilitates quick and each access to multiple selected sources of specific guidance.

## Additional files

Additional file 1: Search strategy for identifying guidance documents for the evaluation of public health interventions. (DOCX 24 kb)

Additional file 2: Figure S1. Flow diagram of guidance selection (DOCX 24 kb)

Additional file 3: References to 48 Guides to Evaluation, that were Summarised and Content Categorized together with Short Titles Used for Each. (DOCX 20 kb)

Additional file 4: Page summaries of 48 guidance documents. (DOCX 173 kb)

Additional file 5: Table S1. Overarching table listing each of the 48 guides and the key pieces of content contained within them. Covering: Background to evaluation and pre-evaluation preparatory work. **Table S2.** Overarching table listing each of the 48 guides and the key pieces of content contained within them. Covering: evaluation processes, types of evaluation and additional support. (DOCX 24 kb)

## Abbreviations

CDC: Centres for disease control and prevention
EBM: Evidence based medicine
MRC: Medical research council
NICE: National institute for health and care excellence
WHO: World Health Organisation
