# Supplementary material for: A review of Grey and academic literature of evaluation guidance relevant to public health interventions
Source: BMC Health Serv Res. 2017 Sep 12;17:643. doi: 10.1186/s12913-017-2588-2 (PMC5596848; doi:10.1186/s12913-017-2588-2)
Supplement: Supplementary file 1 — Search strategy for identifying guidance documents for the evaluation of public health interventions. (DOCX 24 kb) [file 12913_2017_2588_MOESM1_ESM.docx]

**Search strategy for identifying guidance documents for the evaluation of public health interventions**

Guidance documents will be identified using a number of strategies: (1) searching electronic databases (2) hand searching of existing documents and journals (3) searching internet resources (4) citation searching and (5) contacting key authors and professionals in the field.

1. **Electronic database search**

EMBASE, MEDLINE, MEDLINE-in-process, Health management information consortium (HMIC), Social Policy and Practice (SSP), Web of Science, and PsycINFO will be searched using the following search strategy. Search terms will be adapted for each database.

| 1. Guid$  2. Tool$  3. Manual  4. Framework  5. Model$  6. Document  7. Report  8. Resource$  9. Handbook  10. Workbook  11. Standards  12. Training  13. Educat$  14. /or 1-11  15. Evidence  16. Evaluat$  17. Effect$  18. Outcome$  19. Process  20. Impact assessment  21. Indicator  22. Sustainability  23. Efficac*  24. Program development  25. Program evaluation  26. Public health practice  27. /or 13-26  28. Public health  29. Practitioner  30. Professional  31. Manager  32. Evaluator  33. Assessor  34. /or 28-33  35. /and 14,27,34 |
| --- |

**^Table 1: Search strategy for electronic databases^**

1. **Hand searching reference lists and key journals**

Reference lists of included papers will be studied. The following journals will also be examined.

| 1. Public Health 2. BMC Public Health 3. International journal of public health 4. Annual review of public health 5. Journal of public health policy 6. Evaluation 7. Evaluation Review 8. Evaluation and Program Planning 9. Preventing Chronic Disease 10. Canadian Journal of Public Health |
| --- |

**^Table 2: Relevant journals^**

1. **Internet resources**

We will also conduct a search of search engines (Google, BING, Yahoo, WebCrawler) using a modification of the search strategy presented in table 1. We will screen the first 30 pages retrieved with each search. In addition, the websites presented in table 3 will be searched using the term “evaluation.”

| 1. Agency for Health Care Research and Quality via http://www.ahrq.gov 2. Australian Clinical Practice Guidelines Portal via http://www.clinicalguidelines.gov.au/ 3. Australian department of health via http://www.health.gov.au/ 4. Campbell Collaboration via http://www.campbellcollaboration.org/ 5. Centres for Disease Control and Prevention (CDC) via http://www.cdc.gov/ 6. Department of Health (DoH) via https://www.gov.uk/government/organisations/department-of-health 7. European Centre of Disease Prevention and Control via http://www.ecdc.europa.eu 8. Faculty of Public Health via http://www.fph.org.uk/ 9. Guidelines & Audit Implementation Network via http://www.gain-ni.org/ 10. Health Protection Scotland via http://www.hps.scot.nhs.uk/ 11. Health & Social Care Information Centre via http://www.hscic.gov.uk/ 12. Institute of Medicine (IoM) via http://iom.edu/ 13. McMaster University Health Evidence via http://www.healthevidence.org/ 14. MEASURE via https://training.measureevaluation.org/ 15. Medical Research Council (MRC) via http://www.mrc.ac.uk/index.htm 16. NICE Evidence http://www.evidence.nhs.uk/ 17. National Collaborating Centre for Methods and Tools via http://www.nccmt.ca/ 18. National Obesity Observatory (NOO) via http://www.noo.org.uk/ 19. NHS England/Scotland/Wales/Northern Ireland 20. National Guideline Clearinghouse via http://www.guideline.gov/ 21. National Institutes of Health (NIH) http://nih.gov/ 22. National institute for Health Care Excellence (NICE) via http://www.nice.org.uk/ 23. National institute of Health Research (NIHR) via http://www.crn.nihr.ac.uk/ 24. NIH US National Library of Medicine via https://www.nlm.nih.gov/ 25. Parliamentary committees 26. Public Health Agency of Canada via http://www.phac-aspc.gc.ca/index-eng.php 27. Public Health research consortium via http://phrc.lshtm.ac.uk/ 28. Public Health England via https://www.gov.uk/government/organisations/public-health-england 29. The World Bank via http://www.worldbank.org/ 30. Turning Research Into Practice via http://www.tripdatabase.com/ 31. UNAIDS via http://www.unaids.org/en/ 32. US Department of Health and Human Services via https://www.hhs.gov/ 33. WK Kellogg Foundation via http://www.wkkf.org/ 34. World Health Organisation (WHO) via http://www.who.int/en/ |
| --- |

**^Table 3: Specific websites to be searched^**

1. **Citation searching**

Highly specific and relevant articles retrieved using the previous strategies will be identified and articles citing these papers will be obtained.

1. **Contact key figures**

Key authors and experts in the field will be asked to suggest documents/websites/policies we may have missed. Government departments (e.g. public health departments, public health observatories etc) will be contacted directly and asked for any relevant documentation not freely available on the web.

**Inclusion criteria**

Include all documents, websites, books, journal articles, policy recommendations, educational resources, tools and frameworks that provide support to public health practitioners reviewing or undertaking evaluations of public health interventions. Documents may be:

- Resources supporting the conduct of evaluations or an aspect of evaluation (including monitoring an intervention, collaboration, implementation and dissemination);
- Principles that practitioners should follow when conducting evaluations (including economic and process evaluations)
- Resources that help practitioners decide how and when to evaluate interventions
- Standards of good or best practice;
- Recourses supporting identification of outcome indicators
- Resources to help practitioners assess quality of evidence / principles of effectiveness
- Resources to help practitioners identify useful interventions
- Resources to help support practitioners make informed decisions about whether an intervention is likely to be effective in their practice

Documents and articles in which the aim is to evaluate a specific intervention (as opposed to informing others how to evaluate) will be excluded. Development of questionnaires or assessment scales for specific interventions will also be excluded.
