# Supplementary material for: A review of Grey and academic literature of evaluation guidance relevant to public health interventions
Source: BMC Health Serv Res. 2017 Sep 12;17:643. doi: 10.1186/s12913-017-2588-2 (PMC5596848; doi:10.1186/s12913-017-2588-2)
Supplement: Supplementary file 2 — Flow diagram of guidance selection (DOCX 24 kb) [file 12913_2017_2588_MOESM2_ESM.docx]

Articles identified through search (n = 402)

Articles excluded (n= 304)

- Not freely available
- Not post 2000
- Not sourced or created by a national or international organisation
- Not relevant to public health

Title and abstracts screened (n = 402)

Documents reviewed in full (98)

Documents included in the final guide

**Figure 1: Flow diagram of guidance selection**
