# Supplementary material for: A review of Grey and academic literature of evaluation guidance relevant to public health interventions
Source: BMC Health Serv Res. 2017 Sep 12;17:643. doi: 10.1186/s12913-017-2588-2 (PMC5596848; doi:10.1186/s12913-017-2588-2)
Supplement: Supplementary file 3 — References to 48 Guides to Evaluation, that were Summarised and Content Categorized together with Short Titles Used for Each. (DOCX 20 kb) [file 12913_2017_2588_MOESM3_ESM.docx]

**References to 48 Guides to Evaluation, that were Summarised and Content Categorized together with Short Titles Used for Each.**

1. AHRQ. Shekelle PG, Maglione MA, Luoto J, Johnsen B, Perry TR. (2013) Global Health Evidence Evaluation Framework. Research White Paper (Prepared by the Southern California Evidence-based Practice Center under Contract No. 290-2007-10062-I). AHRQ Publication No. 13-EHC008-EF. Rockville, MD: Agency for Healthcare Research and Quality. Accessed December 2015, [www.effectivehealthcare.ahrq.gov](http://www.effectivehealthcare.ahrq.gov).
2. APCRC. Avon Primary Care Research Collaboration (n.d.) Avon Primary Care Research Collaborative Website. Retrieved December 2015, from <http://www.apcrc.nhs.uk/about/>
3. Better Evaluation. Better evaluation. (n.d.). *Better Evaluation; sharing information to improve evaluation.* Retrieved 2^nd^ November, 2015, from <http://betterevaluation.org/>
4. Capacity for Health. Capacity4Health (n.d.). Resource Library. *Capacity for Health.* Retrieved 2^nd^ November, 2015, from <http://www.capacity4health.org/resources/>?
5. CDC1 Evaluation framework. Centres for Disease Control and Prevention (n.d.) A Framework for Program Evaluation. *Program Performance and Evaluation Office (PPEO) – Program Evaluation.* Retrieved 2^nd^ November, 2015, from <http://www.cdc.gov/eval/framework/>
6. CDC2 Evaluation plan. Centers for Disease Control and Prevention, National Center for Chronic Disease Prevention and Health Promotion, Office on Smoking and Health; Division of Nutrition, Physical Activity, and Obesity, (2011). *Developing an Effective Evaluation Plan*. Atlanta, Georgia: Retrieved 2^nd^ November, 2015, from <http://www.cdc.gov/obesity/downloads/CDC-Evaluation-Workbook-508.pdf>
7. CDC3 Evaluation guide. Centers for Disease Control and Prevention, National Center for Environmental Health, Division of Environmental Hazards and Health Effects, Air Pollution and Respiratory Health Branch. (2010) *Learning and Growing through Evaluation: State Asthma Program Evaluation Guide: Module 1*. Atlanta, GA: Retrieved 2^nd^ November, from <http://www.cdc.gov/asthma/program_eval/Evaluation_Guide_COMBINED_FINAL_BOOKMARKED.pdf>
8. CDC4 Implementing evaluation. Centers for Disease Control and Prevention, National Center for Environmental Health, Division of Environmental Hazards and Health Effects, Air Pollution and Respiratory Health Branch. (2010) Learning and Growing through Evaluation: Implementing Evaluations: Module 2. Atlanta, GA: Retrieved 2nd November, from <http://www.cdc.gov/asthma/program_eval/LG-Mod2_DraftFinal_Allsections_Wordaym.pdf>
9. CDC5 Process evaluation. Centers for Disease Control and Prevention, National Center for Chronic Disease Prevention and Health Promotion, Office on Smoking and Health, (2008). *Introduction to Process Evaluation in Tobacco Use Prevention and Control.* Atlanta, GA: U.S. Department of Health and Human Services, Retrieved 2^nd^ December 2015, from <http://www.cdc.gov/tobacco/publications/index.htm>.
10. CDC6 Evaluation resources. Centers for Disease Control and Prevention (n.d.) Other Evaluation Resources. *Program Performance and Evaluation Office (PPEO) – Program evaluation.* Retrieved on 2^nd^ December 2015, from <http://www.cdc.gov/eval/resources/index.htm>
11. Charities Evaluation Service. Charities Evaluation Services (n.d.) *Charities Evaluation Services.* Retrieved 2^nd^ December, from <http://www.ces-vol.org.uk/tools-and-resources/terms-and-definitions/>
12. DFID evaluation guide. Department for International Development (2005) Guidance on Evaluation and Review for DFID staff. Retrieved December 2015, from <https://www.gov.uk/government/uploads/system/uploads/attachment_data/file/67851/guidance-evaluation.pdf>
13. ECDPC Assessing evidence. European Centre for Disease Prevention and Control (2011). *Evidence-based methodologies for public health – How to assess the best available evidence when time is limited and there is lack of sound evidence.* Stockholm: ECDC. Retrieved from <http://ecdc.europa.eu/en/publications/Publications/1109_TER_evidence_based_methods_for_public_health.pdf>
14. European Evaluation Society. European Evaluation Society (n.d.) *European Evaluation Society.* Retrieved 2^nd^ December 2015, from <http://www.europeanevaluation.org/>
15. EMCDDA Evaluation resource kit. European Monitoring Centre for Drugs and Drug Addiction (EMCDDA) (2010) Prevention and Evaluation Resources Kit (PERK) Luxembourg: Publications Office of the European Union. Retrieved on 2^nd^ December, from <http://www.emcdda.europa.eu/attachements.cfm/att_105843_EN_Manual4PERK.pdf>
16. Evaluation Support Scotland. Evaluation Support Scotland (n.d.) *Evaluation Support Scotland.* Retrieved 2^nd^ December 2015, from <http://www.evaluationsupportscotland.org.uk/>
17. Evaluability Assessment. Davis, R., (2013) Planning Evaluability Assessments: A Synthesis of the Literature with Recommendations. Department for International Development (DFID). Retrieved December, 2015, from <https://www.gov.uk/government/uploads/system/uploads/attachment_data/file/248656/wp40-planning-eval-assessments.pdf>
18. Food standards agency. Etienne, J (2015) Introduction to evaluation for Local Authorities. Food Standards Agency. Retrieved on 2^nd^ December, 2015, from <http://www.food.gov.uk/sites/default/files/Guidance%20-%20Introduction%20to%20evaluation%20for%20local%20authorities.pdf>
19. First Nations evaluation. Health Canada (n.d.) A Guide for First Nations on Evaluating Health Programs. Retrieved December 2015, from <http://www.hc-sc.gc.ca/fniah-spnia/pubs/finance/_agree-accord/guide_eval_prog/index-eng.php>
20. The Green Book. HM Treasury, (2011) The Green Book: Appraisal and Evaluation in Central Government. London, TSO. Retrieved December 2013, from <https://www.gov.uk/government/uploads/system/uploads/attachment_data/file/220541/green_book_complete.pdf>
21. Health Scotland Mental Health Improvement. National Health Service (NHS) Health Scotland (2005) Mental Health Improvement: Evidence and Practice – Guide 1: Evidence based practice. Health Scotland, Edinburgh. Retrieved December 2015, from <http://www.healthscotland.com/documents/457.aspx>
22. JRF Community Evaluation. Taylor, M., Purdue, D., Wilson, M., Wilde, P (2005) Evaluating community projects: A practical guide. Joseph Rowntree Foundation. Retrieved December 2015, from <https://www.jrf.org.uk/report/evaluating-community-projects-practical-guide>
23. LEAP. National Health Service (NHS) Health Scotland (2003) LEAP for Health; Learning, evaluation and planning. Health Scotland, Edinburgh. Retrieved December 2015, from <http://www.healthscotland.com/uploads/documents/308-LEAP_for_health.pdf>
24. Magenta Book. HM Treasury (2011). The Magenta Book Guidance for Evaluation. Retrieved December 2015, from <https://www.gov.uk/government/uploads/system/uploads/attachment_data/file/220542/magenta_book_combined.pdf>
25. MRC1 framework. Craig, P., Dieppe, P., Macintyre, S., Michie, S., Nazareth, I., Petticrew, M (2008) Developing and evaluating complex interventions: new guidance. UK Medical Research Council Guidance (MRC) Retrieved December 2015, from <http://www.mrc.ac.uk/documents/pdf/complex-interventions-guidance/>
26. MRC2 Process evaluation. Moore, G., Audrey, S., Barker, M., Bond, L., Bonell, C., Hardeman, W., Moore, L., O’Cathain, A., Tinati, T., Wight, D., Baird, J (2015) *Process evaluation of complex interventions.* UK Medical Research Council (MRC) guidance. Retrieved December 2015, from <http://decipher.uk.net/wp-content/uploads/2014/11/MRC-PHSRN-Process-evaluation-guidance.pdf>
27. MRC3 natural experiments. Craig, C., Cooper, C., Gunnell, D., Haw, S., Lawson, K., Macintyre, S., Oglivie, D., Petticrew, M., Reeves, B., Sutton, M., Thompson, S (2010) *Using natural experiments to evaluate population health interventions: guidance for producers and users of evidence.* UK Medical Research Council (MRC) guidance. Retrieved December 2015, from <http://www.behaviourworksaustralia.org/wp-content/uploads/2012/10/NaturalExperimentsGuidance_MRC-guidance.pdf>
28. NSF Project Evaluation. Westat, J (2002) *The 2002 User Friendly Handbook for Project Evaluation.* The National Science Foundation, Directorate for Education and Human Resources, Division of Research, Evaluation and Communication. Retrieved December 2015, from <http://www.nsf.gov/pubs/2002/nsf02057/nsf02057.pdf>
29. NHS Scotland. National Health Service (NHS) Scotland (n.d.) Evaluation. Accessed December 2015, <http://www.healthscotland.com/scotlands-health/evaluation/index.aspx>
30. NIHR. National Institute for Health and Care Excellence (2010) Behaviour change: evaluation. Accessed December 2015, <http://pathways.nice.org.uk/pathways/behaviour-change/behaviour-change-evaluation>
31. PHE1 introduction. Cavill, N., Roberts, K., Ells, L (2015) Evaluation of weight management, physical activity, and dietary interventions: an introductory guide. Oxford: Public Health England. Accessed December 2015, <http://www.noo.org.uk/securefiles/151202_1535/EvaluationIntroductory.pdf>
32. PHE2 Resources. Public Health England (n.d.) Collection of Resources on Evaluation (CoRE). Public health England. Accessed December 2015, <http://www.noo.org.uk/core>
33. PHE3 Dietary interventions. Roberts, K., Cavill, N., Rutter, H., (2012) Standard Evaluation Framework for dietary interventions. Public Health England. Accessed December 2015, <http://www.noo.org.uk/uploads/doc/vid_16724_SEF_Diet.pdf>
34. PHE4 Physical Activity Interventions. Roberts, K., Cavill, N., Rutter, H., (2012) Standard Evaluation Framework for physical activity interventions. Public Health England. Accessed December 2015, <http://www.noo.org.uk/uploads/doc/vid_16722_SEF_PA.pdf>
35. PHE5 Weight Management Interventions. Roberts, K., Cavill, N., Rutter, H., (2012) Standard Evaluation Framework for weight management interventions. Public Health England. Accessed December 2015, <http://www.noo.org.uk/uploads/doc721_2_noo_SEF%20FINAL300309.pdf>
36. Treasury Board Canada. Treasury Board of Canada, Secretariat. Treasury Board of Canada, Secretariat (n.d.) Program Evaluation Methods: Measurement and Attribution of Program Results: Third edition. Treasury Board of Canada. Accessed December 2015, <http://www.tbs-sct.gc.ca/cee/pubs/meth/pem-mep-eng.pdf>
37. UKES. United Kingdom Evaluation Society (UKES) (n.d.) UK Evaluation Society. Accessed December 2015, <http://www.evaluation.org.uk/>
38. UNEG. United Nations Evaluation Group (2005) Norms for Evaluation in the UN System. United Nations Evaluation Group. Accessed December 2015, <http://www.evaluation.org.uk/>
39. UNDP. United Nations Development Programme (2009) Handbook on Planning, Monitoring, and Evaluating for Development Results. United Nations. Accessed December 2015, <http://web.undp.org/evaluation/handbook/preface.html>
40. UNW. United Nations Women (n.d) (2015) Programming Essentials, Monitoring and Evaluation. United Nations Women. Accessed December 2015, <http://www.endvawnow.org/en/modules/view/14-programming-essentials-monitoring-evaluation.html%20-%2014>
41. USDHH1 Cost effectiveness. Honeycutt, A., Clayton, L., Khavjou, O., Finkelstein, E., Prabhu, M., Blitstein, J., Evans, W., Renaud, J., (2006) Guide to Analyzing the cost-effectiveness of community public health prevention approaches. U.S. Department of Health and Human Services, Office of the Assistant Secretary for Planning and Evaluation. Retrieved December 2015, from <http://aspe.hhs.gov/sites/default/files/pdf/74686/report.pdf>
42. USDHH2 Evaluation. Administration for children and families, office of planning, research and evaluation (2010) The Program Manager’s guide to evaluation (second edition). US. Department of Health and Human Services. Retrieved December 2015, from <http://www.acf.hhs.gov/sites/default/files/opre/program_managers_guide_to_eval2010.pdf>
43. UNAIDS. Joint United Nations Programme on HIV/AIDS (UNAIDS) (2008) A framework for monitoring and evaluating HIV prevention programs for most at risk populations. UNAIDS. Accessed December 2015, from <http://www.unaids.org/sites/default/files/en/media/unaids/contentassets/documents/document/2010/17_Framework_ME_Prevention_Prog_MARP_E.pdf>
44. Well-Being Evaluation Tools . Abdallah, S., Steuer, N., Marks, N., Page, N (2008) Well-Being Evaluation Tools: A Research and Development Project for the Big Lottery Fund. Retrieved 2nd November, 2015, from [www.biglotteryfund.org.uk/wellbeing_evaluation_tools.pdf](http://www.biglotteryfund.org.uk/wellbeing_evaluation_tools.pdf)
45. The World bank. The World Bank (2004) Monitoring and Evaluation (M&E): Some tools, methods and Approaches. Washington, The World Bank. Accessed December 2015, <http://lnweb90.worldbank.org/oed/oeddoclib.nsf/24cc3bb1f94ae11c85256808006a0046/a5efbb5d776b67d285256b1e0079c9a3/$FILE/MandE_tools_methods_approaches.pdf>
46. W.K.Kellogg. W.K. Kellogg Foundation (2004) Evaluation Handbook. W.K.Kellogg Foundation. Accessed December 2015, from <http://www.wkkf.org/resource-directory/resource/2010/w-k-kellogg-foundation-evaluation-handbook>
47. WHO1. Tan-Torres Edejer, T., Baltussen, R., Adam, T., Hutubessy, R., Acharya, A., Evans, D., Murray, C (2003) WHO guide to cost-effectiveness analysis. WHO. Accessed December 2015, from <http://www.who.int/choice/publications/p_2003_generalised_cea.pdf>
48. WHO2. World Health Organisation (2013) WHO Evaluation practice handbook. Switzerland, WHO. Accessed December 2015, from <http://apps.who.int/iris/bitstream/10665/96311/1/9789241548687_eng.pdf>
