# Supplementary material for: A review of Grey and academic literature of evaluation guidance relevant to public health interventions
Source: BMC Health Serv Res. 2017 Sep 12;17:643. doi: 10.1186/s12913-017-2588-2 (PMC5596848; doi:10.1186/s12913-017-2588-2)
Supplement: Supplementary file 4 — Page summaries of 48 guidance documents. (DOCX 173 kb) [file 12913_2017_2588_MOESM4_ESM.docx]

# **Page summaries of 48 guidance documents**

| Title | Better Evaluation |
| --- | --- |
| Short title | Better Evaluation |
| Type of document/ accessibility | Webpage |
| Length | Not applicable |
| Lead author / organisation | Better Evaluation |
| Date | Current. Updated on ongoing basis |
| Source | <http://betterevaluation.org/> |
| Purpose and utility of guidance | Support people throughout an evaluation process |
| Primary target audience | Those who are new to evaluation although it could be usefully used by anyone who wants to plan an evaluation and by teachers |
| Contextual information | Better evaluation is an international collaboration to improve evaluation practice and theory by sharing and generating information about options and approaches. |
| Summary / overview | This is a a comprehensive tool and includes:Evaluation options – engaging stakeholders, recruiting staff, getting funding and so on.Discussion of different evaluation approaches – appreciative enquiry, case studies, democratic evaluation, horizontal evaluationLinks to webinairs, workshops, toolkits and events with further supportCase studies from different part of the worldLinks to new evaluation materialIt provides detailed support on everything needed to support evaluation and assumes no previous research or evaluation knowledge including such topics as determining causal relationships and combining qualitative and quantitative date |
| Strengths | This is an excellent clear and comprehensive guide which takes the reader step by step through the areas needed to carry out an evaluation from engaging stakeholders and considering ethics to developing capacity and meta evaluations.Provides a tool which allows people to carry out an evaluation by making them consider the different stages and requirementsCan be used by someone without evaluation experienceAvailable in many languagesInternational collaborationAllows experts to add new content and develop aspects of the site |
| Limitations | Does not touch on economic evaluation, possibly as it is aimed at those who are new to evaluation and it might be difficult to include this. However it would have been useful to have referred to this. |
| Link to the resources | http://betterevaluation.org/ |
| Links to related resources |  |
| Additional comments | It allows people to join and become part of an evaluation communityIt is updated by members regularly and new material is added and therefore worth monitoring. |

| Title | Well being evaluation tools – a how to handbook: Developed for use as part of the Big Lottery Fund National Well-being Evaluation |
| --- | --- |
| Short title | Well-being evaluation tools |
| Type of document/ accessibility | Online Pdf |
| Length | 18 |
| Lead author / organisation | Big Lottery Fund (England) |
| Date | January 2009 |
| Source | [**http://www.biglotteryfund.org.uk/-/media/Files/Research%20Documents/Wellbeing%20in%20England/**](http://www.biglotteryfund.org.uk/-/media/Files/Research%20Documents/Wellbeing%20in%20England/BigLotteryHowtoHandbookFinal.pdf) |
| Purpose and utility of guidance | The handbook is intended for use as part of the Big Lottery wellbeing programme and the two partners involved in its changing spaces programme.  It is a practical guide to measure the outcomes of these well-being projects, and provides guidance on the evaluation tools which were developed to be used for recipients of the lottery grants. |
| Primary target audience | Portfolio leads, award partners, project managers and project workers who are funded by the Big Lottery measure the outcomes of their projects |
| Contextual information | The Big Lottery The Well-being Programme is a £160 million BIG Programme supporting  projects across the country working on three themes:   - Healthy eating - Physical activity - Mental well-being   The programme is structured into 17 portfolios, each holding a selection  of projects addressing at least one of the above themes. Portfolios have  received funding for 3-5 years with most commencing activities in early  2008 |
| Summary / overview | This is a handbook for recipients of the Big Lottery fund’s wellbeing fund designed to support them to evaluate the impact of their funded work. As there are a range of different types of organisations and projects it provides guidance on when a consistent approach is required across all  projects/portfolios involved in the national evaluation and prompts users to think about how they might want to use the tools for their own specific needs.  It describes a range of evaluation tools- all questionnaires including a ‘core’ questionnaire which all projects should use, and are designed to measure distance travelled, who should use the evaluation tools and sample sizes required, how to select the right tools for your project (using a clear flow chart) and to access these tools and fit them with your evaluation plan, how to use the evaluation tools, when to use them and who can give more support with this, how to access data and discusses Ethical approval, consent and confidentiality. Finally it provides further information and support.  It includes:  1. Introduction  2. The evaluation tools – these are tools which are specific to evaluation of Big Lottery projects.  3. Who should use the evaluation tools?  4. Selecting the right tools for your project including a useful flow chart for choosing the right tools  5. Using the evaluation tools  6. Accessing and making use of the data  7. Ethical approval, consent and confidentiality -  8. Further information and support – including online tools and contacts  **S** |
| Strengths | - Clear and easy to use - Written specifically for the needs of its audience. - Useful flow chart for choosing the right tools - Emphasis on effective use of resources and on the recipients. - Sources of further support offered by the Big Lottery National Evaluation Team Including a web page with tools and support from the rapid response team and specific contact details. - Gives contact details for economic evaluation support - Alongside local evaluations there is a national evaluation focusing on the economics of these interventions. - Can be converted to other file formats - Provides links to related resource for registered users. - Outcomes focused |
| Limitations | - Very specific to recipients of Big Lottery Funding and not a great deal of use for other programmes. - Quite rigid methods - No mention of stakeholder engagement - Does not discuss whether outcomes are cost effective or could be better achieved by another measure - The tools it includes for evaluation are all questionnaires containing closed questions (designed for consistency but may not reflect the real work of the projects) - Does not explore what to do about people who drop out of the project |
| Link to the resources | [**http://www.biglotteryfund.org.uk/-/media/Files/Research%20Documents/Wellbeing%20in%20England/**](http://www.biglotteryfund.org.uk/-/media/Files/Research%20Documents/Wellbeing%20in%20England/BigLotteryHowtoHandbookFinal.pdf) |
| Links to related resources | www.cles.org.uk/wellbeing. |
| Additional comments | There is also a linked national evaluation which will use different research methods. |

| Title | Capacity for Health (C4H) Online Resource Library |
| --- | --- |
| Short title | Capacity for Health |
| Type of document/ accessibility | webpage |
| Length | Not applicable |
| Lead author / organisation | Capacity for Health |
| Date | Current |
| Source | Online |
| Purpose and utility of guidance | This is a resource library funded by the Centres for Disease Control and Prevention to support capacity building in HIV projects |
| Primary target audience | Community based organisations working in HIV prevention (unstated but focused on USA) |
| Contextual information | C4H are an organisation which provide free support to Health Departments across the US and affiliated territories. There focus is on AIDS and HIV prevention and they tailor the support that they offer to specific circumstances |
| Summary / overview | The library contains a range of resources to support capacity building including literature, reports and webinairs. It divides these resources into three categories: organizational infrastructure and program sustainability (running an organisation), evidence based intervention and public health strategies (strengthening an organisations’ abilities to implement effective intervention) and monitoring and evaluation (measuring process and outcome and understanding strengths and areas for improvement). All of these areas have links to resources in these topics.  It also provides links to support service, the C4H programme website and index of further tools.  Evaluation specific topics include:   - Evaluation Overview - Types of Evaluation - Evaluation Planning - Needs Assessment - Logic Models - Data Collection Methods - Data Management and Analysis - Sharing Findings - Evaluation Capacity - Participatory Evaluation - Quality Assurance |
| Strengths | - Clear and easy to access, search and read - Resources that it links to are free - Specifies a clear purpose - Provides a link to a wide range of resources |
| Limitations | - The title does not make the purpose of the webpage clear. In particular that the resources focus on HIV work in the US. - Uncertain how these resources were chosen. Was this systematic? Was their quality control. - Not specifically evaluation focused although evaluation is included - Best used by someone with evaluation experience and some idea what they are looking for as someone else could get lost amongst the different resources. - Not clear how this relates to other work in HIV and in evaluation in the US.   Capacity for Health seems to be a programme specifically for those working within Asian and Pacific Islanders community in America but this is not evident on the main web page. |
| Link to the resources | http://library.capacity4health.org/ |
| Links to related resources | Includes a large number of links to other resources |
| Additional comments |  |

| Title | A framework for program evaluation |
| --- | --- |
| Short title | CDC1 Framework |
| Type of document/ accessibility | Website |
| Length | Not Applicable |
| Lead author / organisation | Centres for Disease Control and Prevention (CDC) |
| Date | 1999 |
| Source | Centre for Disease Control and Prevention |
| Purpose and utility of guidance | Use this if you want to evaluate the effects of public health actions |
| Primary target audience | Public health professionals |
| Contextual information | In May 1997, the CDC Director and executive staff decided that there was a need to produce a basic, organisational framework for evaluation in public health, a need to combine evaluation with program management, and a need for evaluation studies that demonstrate the relationship between program activities and prevention effectiveness. CDC convened an Evaluation Working Group, charged with developing a framework that summarises and organizes the basic elements of program evaluation. |
| Summary / overview | The framework is described as a practical, non-prescriptive tool, designed to summarise and organise essential elements of program evaluation. The framework comprises six steps in program evaluation practice and four standards for effective program evaluation. Adhering to the steps and standards of this framework will allow an understanding of each program’s context and will improve how program evaluations are conceived and conducted. Furthermore, the framework encourages an approach to evaluation that is integrated with routine program operations. The emphasis is on practical, ongoing evaluation strategies that involve all program stakeholders, not just evaluation experts.  Specifically, the main purpose of the framework is to:   - Summarise and organise the essential elements of program evaluation - Provide a common frame of reference for conducting effective program evaluations - Clarify steps in program evaluation - Review standards for effective program evaluation; - Address misconceptions about the purposes and methods of program evaluation   These six connected steps and 4 standards provide a systematic way to approach and answer the questions below: What will be evaluated? (That is, what is the program and in what context does it exist?)  - What aspects of the program will be considered when judging program performance? - What standards (i.e., type or level of performance) must be reached for the program to be considered successful? - What evidence will be used to indicate how the program has performed? - What conclusions regarding program performance are justified by comparing the available evidence to the selected standards? - How will the lessons learned from the inquiry be used to improve public health effectiveness?   The Steps:   - Engage stakeholders - Describe the program - Focus the evaluation design - Gather credible evidence - Justify conclusions - Ensure use and share lessons learned   The Standards:   - Utility - Feasibility - Propriety - Accuracy |
| Strengths | - The framework summarizes and organizes essential elements of program evaluation - It guides users in selecting strategies that are useful, feasible, ethical and accurate - Provides guidance on conducting practical evaluation, within the confines of resources, time, and political context - The website provides links to numerous links and information related to the framework - The framework emphasises considerable stakeholder involvement |
| Limitations | - The focus is on practical evaluation, rather than high quality - Assumes basic knowledge of evaluation - The framework itself may be of little use without all the supporting documents. - The volume of information available may be overwhelming |
| Link to the resources | <http://www.cdc.gov/eval/framework/> |
| Links to related resources | 1. <ftp://ftp.cdc.gov/pub/Publications/mmwr/rr/rr4811.pdf> 2. <http://www.cdc.gov/eval/guide/index.htm> 3. <http://www.cdc.gov/asthma/program_eval/Evaluation_Guide_COMBINED_FINAL_BOOKMARKED.pdf> 4. <http://www.cdc.gov/tb/programs/evaluation/TBEvaluationHandbook_tagged.pdf> 5. <http://www.cdc.gov/tobacco/tobacco_control_programs/surveillance_evaluation/evaluation_manual/pdfs/evaluation.pdf> 6. <http://www.cdc.gov/healthyyouth/evaluation/index.htm> 7. <http://www.cdc.gov/dhdsp/docs/evaluation_framework.pdf> 8. <http://www.cdc.gov/eval/guide/execsummary/index.htm> 9. <http://www.cdc.gov/eval/resources/index.htm> |
| Additional comments |  |

| Title | Developing an Effective Evaluation Plan: Setting the course for effective program evaluation |
| --- | --- |
| Short title | CDC2 Evaluation plan |
| Type of document/ accessibility | Online pdf |
| Length | 115 |
| Lead author / organisation | National Center for Chronic Disease Prevention and Health Promotion. Centers for Disease Control and Prevention’s (CDC’s) Office on Smoking and Health (OSH) and Division of Nutrition, Physical Activity, and Obesity (DNPAO). |
| Date | 2011 |
| Source | Online |
| Purpose and utility of guidance | Part of a series of workbooks which are intended to offer guidance and facilitate capacity building on a wide range of evaluation topics. They can be adapted to meet specific program’s evaluation needs.  It is not a complete ‘how to’ guide but should be used alongside other evaluation resources and some of these are listed. |
| Primary target audience | Program managers and evaluators working in any public health program. |
| Contextual information | CDC are the US public health agency responsible for the prevention of chronic disease |
| Summary / overview | This workbook applies the CDC Framework for Program Evaluation in Public Health which sets out a six-step process for the decisions and activities involved in conducting an evaluation.  Part I of this workbook defines and describes how to write an effective evaluation plan going through the six steps:  The Process of Participatory Evaluation Planning – (1) engage stakeholders,(2) define the purpose of the evaluation (3) Describe the program- shared understanding, narrative description, logic model, stages of development, focus the evaluation and develop the questions, (4) Budget and Resources (5) Planning gathering evidence, methods, credible evidence, measuring, data sources and methods, roles and Responsibilities (6) Planning for Conclusions- Planning for Dissemination and Sharing of Lessons Learned - communication and dissemination Plans and ensuring use.  Part II of this workbook includes exercises, worksheets, tools, and a Resource Section to facilitate program staff and evaluation stakeholder workgroup (ESW) thinking through the concepts presented in Part I of this workbook.  There are exercises and tools for each of the steps which allows the user to put all of the steps into practice in their own project by choosing the worksheets and tools which are relevant to them. |
| Strengths | - Clearly written and comprehensive - Clear about audience and aim - Takes readers step by step through the evaluation process and provides tools at each stage. - Includes budgeting - Includes planning for change - Refers to further resources |
| Limitations | - Could usefully have a discussion of policy especially given it is a government agency - Does not mention economic effectiveness of programme. - Would be improved by more worked examples |
| Link to the resources | http://www.cdc.gov/obesity/downloads/CDC-Evaluation-Workbook-508.pdf |
| Links to related resources | The CDC framework for evaluation  http://www.cdc.gov/eval/framework/index.htm |
| Additional comments | Might be useful to consider all the CDC workbooks together that were accessed in this search |

| Title | Implementing Evaluations: Learning and Growing Through Evaluation. State Asthma Program Evaluation (module 1) |
| --- | --- |
| Short title | CDC3 Evaluation guide |
| Type of document/ accessibility | Online pdf |
| Length | 149 |
| Lead author / organisation | CDC National Center for Chronic Disease Prevention and Health Promotion. |
| Date | April 2010 |
| Source | Web |
| Purpose and utility of guidance | This is part of a series of evaluation documents. The first module in this series focuses on planning for evaluation. The second module builds on this to support implementing planning. Module 3 applies these tools to the evaluation of state asthma program partnerships. |
| Primary target audience | This guide is intended to be used by state and territorial public health departments (SHDs) that are receiving CDC funding for state asthma programs. |
| Contextual information | The CDC is the nation’s health protection agency. The goal of the CDC is to protect America from health, safety and security threats – both foreign and the US.  This series of guides was developed for the National Asthma Control Program, and relates to the CDC framework – and applies this framework to evaluation of asthma programs. |
| Summary / overview | The guide begins with an overview of the CDC framework for evaluation of public health programs.  Chapter two provides detail on how to develop a strategic evaluation plan (i.e. the overview of multiple evaluations that will occur over a specific time period). This includes: Step A. Establishing an Evaluation Planning Team; Step B. Describing the Program; Step C. Prioritizing Program Activities for Evaluation (e.g., cost, sustainability etc); Step D. Considering Evaluation Design Elements; Step E. Developing a Cross-Evaluation Strategy; Step F. Promoting Use through Communication (i.e., communicate the findings to improve program development); and Step G. Writing and Revising Your Strategic Evaluation Plan  Chapter three provides detail on how to develop an Individual Evaluation Plan (as included in the larger, more comprehensive strategic evaluation plan). This includes: Step 1. Engage Stakeholders; Step 2. Describe What is Being Evaluated; Step 3. Focus the Evaluation Design; Step 4. Gather Credible Evidence; Step 5. Justify Conclusions; and Step 6. Ensure Use of Evaluation Findings and Share Lessons Learned. |
| Strengths | - Good clear overview of the use of the framework applied to an evaluation of an asthma program - Informs readers how each section relates to the CDC framework - Provides vignettes to aid understanding - The appendices contain a number of useful toolkits to support planning evaluations |
| Limitations | - Does not discuss evaluation theory - Examples largely relate to asthma - Lack of information on economics of evaluation |
| Link to the resources | <http://www.cdc.gov/asthma/program_eval/Evaluation_Guide_COMBINED_FINAL_BOOKMARKED.pdf> |
| Links to related resources | <http://www.cdc.gov/asthma/program_eval/LGMod2_DraftFinal_Allsections_Wordaym.pdf>  <http://www.cdc.gov/asthma/program_eval/module_3.pdf> |
| Additional comments | Part of a series of modules and should be read in that context. |

| Title | Implementing Evaluations: Learning and Growing Through Evaluation (Module 2) |
| --- | --- |
| Short title | CDC4 Implementing evaluations |
| Type of document/ accessibility | Online pdf |
| Length | 135 |
| Lead author / organisation | National Centre for Chronic Disease Prevention and Health Promotion. |
| Date | August 2011 |
| Source | Web |
| Purpose and utility of guidance | This is part of a series of evaluation documents. The first module in this series focuses on planning for evaluation. This module builds on that to support implementing planning. Module 3 applies these tools to the evaluation of state asthma program partnerships. |
| Primary target audience | Anyone involved in implementing evaluations |
| Contextual information | CDC are the US public health agency responsible for the prevention of chronic disease |
| Summary / overview | The previous module discussed evaluation planning. Moving on from this, the second module discusses implementing evaluations. The first section of this module (2) identifies methods for moving from planning to implementation by using information from evaluation planning to inform evaluation implementation. It also describes the difference between various planning and implementation teams as well as appropriate team members. The second chapter takes trainees though different stages of an evaluation, the stakeholders, developing an evaluation process, training in data collection, monitoring and ongoing communication and developing an action plan, linking back to the original plan and successfully implementing change.  The document covers:   - Strategies for successful implementation- working with stakeholders, developing a process for managing the evaluation, Pilot Testing, Training for data collection, getting a team together, monitoring progress and promoting ongoing communication, Interim report and dissemination, developing an action, lessons learned, and linking back to Strategic Evaluation   Appendices are included on:   - Meeting Evaluation Challenges, Evaluation Anxiety, Common Evaluation Designs, Budgeting for Evaluation, Evaluation Management Toolkit, Gathering Credible Evidence, Training and Supervising Data Collectors, Effective Communication and Reporting, Developing an Action Plan |
| Strengths | - Builds on planning for evaluation to take readers step by step onto linking this to implanting evaluation. - Could be used on its own to implement evaluation - Emphasises engaging the right people at the start - Stresses that an evaluation plan is an implementation plan and focuses on action as a result of the evaluation - Relates evaluation to general project management drawing out similarities. - The appendices contain a number of useful toolkits to support implementation |
| Limitations | - Would be useful if it had stated where it was in the series of modules although it does seem that it is designed to be accessed through the main page which provides context. - This is for evaluating asthma programmes and it would be helpful if this was made clear in the title. Perhaps if it were read within the context of the other modules it would be clearer. - There are aspects of evaluation missing but they may well be part of other training modules. - There is no mention of cost effectiveness or other economic considerations. - Although the plan does consider stakeholders it focuses more on the mechanic of keeping them involved rather than the ethos and the need for them to help to define the goals. |
| Link to the resources | <http://www.cdc.gov/asthma/program_eval/LGMod2_DraftFinal_Allsections_Wordaym.pdf> |
| Links to related resources | <http://www.cdc.gov/asthma/program_eval/Evaluation_Guide_COMBINED_FINAL_BOOKMARKED.pdf>  <http://www.cdc.gov/asthma/program_eval/module_3.pdf> |
| Additional comments | Part of a series of modules and should be read in that context |

| Title | Introduction to process evaluation in tobacco use prevention and control |
| --- | --- |
| Short title | CDC5 Process evaluations |
| Type of document/ accessibility | Online pdf |
| Length | 71 pages |
| Lead author / organisation | Center for Disease Control and Prevention, National Center for Chronic Disease Prevention and Health Promotion, Office on Smoking and Health |
| Date | February 2008 |
| Source | web |
| Purpose and utility of guidance | Intended to provide process evaluation technical assistance. It defines process evaluation and describes the rationale, benefits, key data collection components, and program evaluation management procedures. It also discusses how process evaluation links with outcome evaluation and fits within an overall approach to evaluating comprehensive tobacco control programs.  This manual complements other DC initiatives which concentrate on outcome evaluation. |
| Primary target audience | Office of Smoking and Health staff, grantees and partners |
| Contextual information | CDC are the US public health agency responsible for the prevention of chronic diseases |
| Summary / overview | This manual:  -Provides a framework for understanding the links between inputs, activities, and outputs and for assessing how these relate to outcomes; and  -Can assist state and federal program managers and evaluation staff with the design and implementation of process evaluations for evidence of progress in tobacco control.  It is not a cookbook but instead gives general principles which can be adapted to be used in different circumstances.  1 Introduction to Process Evaluation in Tobacco Use Prevention and Control   - The Planning/Program Evaluation/Program Improvement Cycle - Distinguishing Process Evaluation from Outcome Evaluation – outcome evaluation is about the results of an intervention and process about what is done to achieve these results.   2. Purposes and Benefits of Process Evaluation   - Definition of Process Evaluation and the scope of Tobacco Control Activities Included that is what level of information needs to be collected to answer the question. - Purposes of process evaluation and Monitoring, Improvement, Effective Program Model and. Accountability. - Users (those affected by the programme, those who manage and staff it and those who have power over it) and Uses of Process Evaluation Information (how it is helpful to understand outcomes)   3.Information Elements Central to Process Evaluation   - Indicators of Inputs/Activities/Outputs - Comparing Process Information to Performance Criteria   4. Managing Process Evaluation – 10-Step Process   - Divided into 4 stages -Groundwork, formalisation (such as developing the proposal and tools), Implementation, Utilization,   5. Protecting participants-ethical considerations  6. Methods  7. Process Evaluation—Beyond a Single Study – building capacity and incorporating evaluation in an ongoing basis.  8. Conclusion- using process evaluation in conjunction with outcome evaluation you have a better way to focus your time and resources,  Appendices which include framework and standards for evaluation |
| Strengths | - Clear step by step approach supported by examples and appendices for further information - Emphasises that it is not a cook book method but a guide - Useful to focus on process evaluation - Includes a range of case examples which show the use of process evaluation in practice - Discusses the protection of participants |
| Limitations | - Focus only on process evaluation - Focus on tobacco- but could be used in other areas. - No economic considerations |
| Link to the resources | <http://www.cdc.gov/tobacco/tobacco_control_programs>  /surveillance_evaluation/process_evaluation/pdfs/tobaccousemanual_updated04182008.pdf |
| Links to related resources |  |
| Additional comments | Read in conjunction with other CDC work on evaluation and in particular that on outcome evaluation which this is designed to complement.  Those mentioned are: Key Outcome Indicators for Evaluating Comprehensive Tobacco Control Programs and Introduction to Program Evaluation for Comprehensive Tobacco Control Programs. |

| Title | [Program Performance and Evaluation Office (PPEO)](http://www.cdc.gov/program/) - [Program Evaluation](http://www.cdc.gov/eval/index.htm) |
| --- | --- |
| Short title | CDC6 Evaluation Resources |
| Type of document/ accessibility | Online web page |
| Length | Not applicable |
| Lead author / organisation | Centre for Disease Control and Prevention (CDC) |
| Date | Regularly updated |
| Source | Online |
| Purpose and utility of guidance | To support people conduct program evaluation of the CDC’s priorities |
| Primary target audience | Those working in the field of evaluating programs that aim to prevent diseases. This includes evaluators, researchers and policy makers whether they are new or experienced. It is aimed at a US audience but could be usefully used by others. |
| Contextual information | The role of the CDC is to protect American citizens from health, safety and security threats both within and outside the US. In order to do this it conducts research and provides health information to protect people from health threats as well as responding to new threats, |
| Summary / overview | This part of CDC’s work focuses on program evaluation to improve public health and by making evaluation a routine part of work to improve program effectiveness.   - It sets out a clear framework for program evaluation which is in line with other such evaluation frameworks and takes readers very clearly through each step of the framework describing what needs done at that step and how to do it. It links to documents which provide more details on these steps (such as engaging stakeholders, describing the program, focusing the evaluation and gathering evidence all the way through to writing reports and disseminating). - It provides a good overview of standards and how they are used in evaluation. - It outlines learning from evaluation and using results in practice and guidance on recruiting an evaluation team. - It provides a range of documents which presents the evaluation framework in different ways for different audiences. - It provides links to a range of self-study guides for program evaluation - Finally it provides a links to a range of evaluation resources. - The resources which are referred to are detailed and would allow a reader to get an excellent overview and understanding of evaluation. |
| Strengths | - Excellent resource that could be used by new and experienced evaluators. - The information that it does provide is useful and more comprehensive than similar guides. - The layout of the information means that a reader can get an overview of evaluation and then focus on areas were they wish to know more or where there are gaps in their knowledge. - Even those with significant experience could use this usefully - Gives more information on standards than many similar guides. - Clear and comprehensive - Easy to navigate - Provides links to Evaluation guides which describe aspects of evaluation in depth - Particularly useful information on who to include in an evaluation team. - Has clear contact details for further information - Has a search feature |
| Limitations | - US focused - Would be useful if it had set out who the target audience are more clearly - Nothing on economic evidence or comparing programs |
| Link to the resources | <http://www.cdc.gov/eval/index.htm> |
| Links to related resources |  |
| Additional comments | There is a number of papers and web pages about evaluation from CDC and it might be worth considering them together.  Provides links to a range of self-study guides. |

| Title | NCVO Charities Evaluation Services (CES) |
| --- | --- |
| Short title | Charities Evaluation Service |
| Type of document/ accessibility | Web page |
| Length | Not applicable |
| Lead author / organisation | Charities Evaluation Services |
| Date | Current |
| Source | Web |
| Purpose and utility of guidance | This webpage explains what CES is, what services the organisation offers and how to access their consultancy and training services. It is also a resource on self-evaluation for voluntary sector organisations and their funders.  Finally, it links to information on PQASSO, the quality assurance system that CES developed specifically for the voluntary sector. The PQASSO pages are now housed on the NCVO website (see below).  Since this has been reviewed the NCVO and PQASSO team have been restructured |
| Primary target audience | People working in the charitable and voluntary sector, and their funders, who want to improve their evaluation, with a focus on outcomes and impact. |
| Contextual information | CES was established in 1990. It works with voluntary organisations to support and strengthen their ability to carry out evaluation with the aim of making charities more effective. In November 2014 CES merged with NCVO (the National Council for Voluntary Organisations). |
| Summary / overview | This is a webpage that describes what CES does and how a charity can get can further support in evaluation. It also provides links to sources of support for evaluation.  It states:  How CES can help  What training and events they offer and the consultancy service they provide  How they work with funders and with charities  The site provides links to useful tools which support different stages of evaluation including planning, assessing baseline and progress towards target, developing outcomes and indicators, data collection tools and related publications and research. It also lists evaluation tools available within the sector.    The site also provides links to reports, research and examples of evaluations in practice |
| Strengths | - Clear and easy to use site - Provides evaluation support for those working in the voluntary sector and their funders - Has links to consultancy, tools and training. - Has a black and white accessible version although given it is for charities I would have expected other types of access too |
| Limitations | - Little mention of economic evaluation or showing cost effectiveness - Does not go into a great deal of detail on evaluation though the links provide more depth and this is explored further in tools and resources |
| Link to the resources | http://www.ces-vol.org.uk |
| Links to related resources |  |
| Additional comments | CES merged with NCVO in November 2014 |

| Title | Evidence Based Methodologies for Public Health: How to assess the best available evidence when time is limited and there is lack of sound evidence. |
| --- | --- |
| Short title | ECDPC Assessing evidence |
| Type of document/ accessibility | Online Pdf |
| Length | 67 |
| Lead author / organisation | European Centre for Disease Prevention and Control |
| Date | September 2011 |
| Source | Online |
| Purpose and utility of guidance | To support those who give advice on evidence for public health in communicable diseases where evidence is uncertain, the situation is complex or there is short notice to help address the problem that the epidemiology of communicable disease can only be studied during an outbreak and therefore time is short, patient care is required at the same time, there is a need to prevent the disease being spread and the situation is changing on an ongoing basis. |
| Primary target audience | Public health professionals and policy makers who evaluate evidence in order to give advice in public health for communicable diseases |
| Contextual information | The European Centre for Disease Prevention and Control (ECDC) was established to enhance the capacity of the European Community to protect human health through the prevention and control of human diseases – to identify, assess and communicate current and emerging threats by communicable diseases. |
| Summary / overview | This report focuses on how to use methods from evidence based medicine in general to support evidence based public health medicine specifically in the field of communicable disease.  It describes in detail the tenents of evidence based medicine and the specific challenges associated with using this in the field of public health (where there is less evidence on the outcomes of interventions in general and where it is more difficult to use systematic reviews and randomised control trials). It discusses the usefulness of using grading tools for grading evidence in the field of public health.  In accordance with the aims, the document is presented in four main sections: (i) How to give evidence based guidance with evidence is scarce and time is limited (ii) the usefulness of using evidence based tools for grading evidence in the field of communicable diseases (iii) assessing the quality of guidelines for preventing and controlling communicable diseases and (iv) using consensus methods for decision making.  With regard to giving guidance when resources are scarce, the document presents a five-stage framework for rapid risk assessments. This includes a preparatory phase and further stages of risk detection/verification, assessment of the risk, development of the advice and implementation and evaluation. Practical tools and templates for each stage are also presented, and the importance of being prepared and having tools at hand when an outbreak occurs is underlined.  The usefulness of evidence-based methods and grading tools are explored in the next section. A variety of methods for reporting, assessing and grading evidence are identified and the applicability of these tools in a public health setting is discussed. Many tools required to produce evidence-based advice already exist, but there is a need to further develop instruments and checklists for some of the study designs relevant to public health.  For assessing the quality of guidelines in relation to infection diseases, an evaluation of the AGREE II tool is presented. The importance of the different domains and items is discussed and some additional criteria for communicable diseases guidelines are proposed.  In the final section, the document suggests that consensus methods can be used both to evaluate the evidence and to improve the balance of subjective interpretation of evidence from systematic reviews and also to develop best available expert judgements in settings with lack of evidence. Consensus methods can be applied by members of a guidance development group and as a method to facilitate implementation in hearing processes among stakeholders. The importance of transparency in public health decision-making, the role of experts and, finally, how to apply different consensus methods under different timelines are discussed. |
| Strengths | Good guide to using evidence assessment methods for public health |
| Limitations | Not about evaluation but about assessing evidence for public health |
| Link to the resources | http://ecdc.europa.eu/en/publications/Publications/1109_TER_evidence_based_methods_for_public_health.pdf |
| Links to related resources |  |
| Additional comments |  |

| Title | European Evaluation Society Website |
| --- | --- |
| Short title | European Evaluation Society |
| Type of document/ accessibility | Webpage |
| Length | Not relevant |
| Lead author / organisation | European Evaluation Society |
| Date | Ongoing updates |
| Source | web |
| Purpose and utility of guidance | This website aims to bring academics, policy makers and practitioners in different geographical and topic areas together to encourage knowledge exchange, good practice dissemination, professional co-operation and bridge building. The society has four strategic aims:  ***1*** *Working with and supporting the development of evaluation societies within and outside Europe.*  ***2:*** [*Thematic working groups (TWGs)*](http://europeanevaluation.org/community/thematic-working-groups-twgs)*.*  ***3****: Capacity development and professionalization.*  *4: Improving the EES financial situation* |
| Primary target audience | Academics, policy makers and practitioners working in evaluation in Europe and beyond |
| Contextual information | The European Evaluation Society promotes the theory, practice and use of high quality evaluation in Europe and beyond.  It does this by bringing together academics, policy makers and practitioners, by setting up events and by connecting people to encourage knowledge exchange, good practice dissemination, professional co-operation and bridge building. |
| Summary / overview | This web page:   - Explains what the European Evaluation Society is and what its aims are - Describes its partners and their work - Provides information on conference and training events and the material at these events - Provides information on vacancies and tenders in evaluation - Has an interactive blog and discussions - Provides links to other resources including glossaries, standards, journals, evaluation tools and libraries on evaluation throughout the world |
| Strengths | - European wide - Single website for jobs, training, conferences, problem solving etc. - A forum for users to discuss problems - Website clear and easy to navigate |
| Limitations | - Some web links broken - Is it available in other languages? - Aimed at experienced evaluators - Does not explain what evaluation is – but aimed at a professional audience working in evaluation |
| Link to the resources | http://www.europeanevaluation.org/ |
| Links to related resources |  |
| Additional comments |  |

| Title | Prevention and Evaluation Resources Kit (PERK): A manual for prevention professionals |
| --- | --- |
| Short title | EMCDDA Evaluation Resource Kit |
| Type of document/ accessibility | Online pdf |
| Length | 100 pages |
| Lead author / organisation | European Monitoring Centre for Drugs and Addiction |
| Date | 2010 |
| Source | online |
| Purpose and utility of guidance | This resource kit compiles basic, evidence-based prevention principles, planning rules and evaluation tips and documents and references for further support. |
| Primary target audience | Professionals working to prevent substance misuse in the EU.  -prevention policy planners, for example, by providing information on which strategies are effective or on how to determine whether a project (proposal) is sound and well designed.  -prevention professionals and project developers, through the provision of background literature, theories, references and evaluation tools. |
|  | The European Monitoring Centre for Drugs and Drug Addiction (EMCDDA) is an EU decentralised agency based in Lisbon. It is the central source of comprehensive information on drugs and drug addiction in Europe. It collects, analyses and disseminates factual, objective, reliable and comparable information on drugs and drug addiction in order to provide an evidence-based picture of the drug  phenomenon at the European level. |
| Summary / overview | - PERK believes that evaluation should be considered at the planning and prevention stage. - Planning- step by step through the development of an intervention and the knowledge base in prevention. Ideas can be added or revised as the project progresses depending on resources and setting. - A compilation of materials, sources and instruments to support the setting up and evaluating of prevention interventions. These were based on training sessions throughout Europe to find out what prevention professionals want. This was models, theories and evaluation principles: not only about elements that work, but also about what is popular but does not work. - Emphasis on evidence base and theoretical underpinning rather than opinion and perspective. - Project leaders guided first to carry out a needs assessment then to use a logic model to development prevention interventions so that the objectives, hypothesis, content and indicators and logically build on each other, relevant and address the problem. - Each step in the evaluation begins with a description of the theory, then the process and then a real life example. - Contains a resource kit with materials that have already been used successfully. - It uses real life examples to illustrate theory. |
| Strengths | - Evidence based source for the EU. - Useful and clear step by step approach - Makes use of existing material which has been used in practice in the EU and US. - A related website which provides further resources and can be adapted as other material becomes available. - Illustrates theory with real examples. - Mentions cost effectiveness and efficiency |
| Limitations | - Not clear what ‘utopia example’ means - Not enough discussion of cost effectiveness - Specifically substance abuse prevention – though could be usefully used with other projects |
| Link to the resources | [http://www.emcdda.europa.eu/attachements.cfm/att_105843_EN_Manual4PERK.pdf#](http://www.emcdda.europa.eu/attachements.cfm/att_105843_EN_Manual4PERK.pdf) |
| Links to related resources |  |
| Additional comments |  |

| Title | Evaluation Support Scotland (ESS) |
| --- | --- |
| Short title | Evaluation support Scotland |
| Type of document/ accessibility | Website |
| Length | NA |
| Lead author / organisation | Evaluation Support Scotland |
| Date | 2005 – ongoing |
| Source | Evaluation Support Scotland |
| Purpose and utility of guidance | This resource is for third sector organisations and funders. Evaluation Support Scotland (ESS) works with third sector organisations and funders so that they can measure and report on their impact and use learning to improve policy and practice. |
| Primary target audience | Those funding or conducting self-evaluation within third sector organisations. |
| Contextual information | ESS supports third sector organisations and funders to be better at measuring their impact, reporting on the difference they make, to deliver better services. |
| Summary / overview | ESS website has free resources, tools, thematic guides, case studies and reports to help third sector organisations and funders to support them to evaluate and learn from the evidence.  The website includes a series of downloadable ESS Support Guides on a number of evaluation topics including:  • Clarifying aims, outcomes, and activities  • Developing a logic model  • Developing and using indicators  • Using interviews and questionnaires  • Visual approaches  • Using technology  • Storing information  • Analysing information  • Writing case studies  • Report writing  • Using qualitative data  • Using what you learn from evaluation  • Getting the best from an external evaluation  • How to budget self-evaluation  A number of practical tools are also provided. This includes templates, route maps, and workbooks to support different stages of evaluation.  Guides organised by themes include; guidance and tools for evaluating arts and sport, children and young people, community and learning development, environment, equalities, health and social care, homelessness, international development, partnership, quality, social enterprise, substance misuse, volunteering, and criminal justice.  Case studies of evaluations (and advantages and disadvantages) are presented. Reports of such evaluations are also available.  The final section on the website provides information on external consultants, as well as a link to a database of external consultants.  An online training module ‘Getting the best from external evaluation’ is available.  Evaluation skills workshops are delivered across Scotland and thematic learning programmes. Resources produced from these programmes are available on the website. |
| Strengths | - Provides ~~a~~ good information and resources about how to self-evaluate for practitioners working in the Third Sector and funders. - Tools, such as flow charts, templates, and route maps are included - Comprehensive in scope of topics covered - Guidance documents linked by theme are available - Case studies of good practice are included to demonstrate evaluation in practice |
| Limitations | - Very simple consideration of the basic processes involved in evaluation - No consideration of quality of evidence although that is considered in another document - Some (but not all) resources are limited to evaluation of third sector projects |
| Link to the resources | http://www.evaluationsupportscotland.org.uk/ |
| Links to related resources |  |
| Additional comments | A Stitch in Time? supports the third sector to collect and present evidence about its contribution to [**Reshaping Care for Older People**](http://www.evaluationsupportscotland.org.uk/media/uploads/reshapingcareprogrammefinal4march.pdf) (RCOP).   This three year programme ran to March 2015 and focused on third sector organisations working with older people and carers in Lothian. |

| Title | Introduction to evaluation for Local Authorities : A brief guide to evaluating food enforcement projects and innovative approaches |
| --- | --- |
| Short title | Food Standards Agency |
| Type of document/ accessibility | Online pdf |
| Length | 22 pages |
| Lead author / organisation | Food Standards Agency |
| Date | March 2015 |
| Source | Online |
| Purpose and utility of guidance | This guide is designed to help Local Authorities (LAs) conduct evaluations related to food standards. It introduces the principles of good evaluation, explains some key concepts, and outlines some things to bear in mind when planning to self-evaluate at LA level. The reader will also find pointers to where to look if in need of more detailed information. |
| Primary target audience | Local Authorities who are conducing evaluations related to food law |
| Contextual information | The Food Standards Agency are a UK government agency which are responsible for monitoring food safety and hygiene across the UK |
| Summary / overview | - Why evaluate? - Evaluation step by step - What is evaluation? – process, impact and the difference between evaluation and monitoring. - Ethics - Principles of evaluation – causality, control, intended and unintended consequences, evaluation design and generalizability large sample approach, small sample approach interpreting, presenting and communicating results - Further resources |
| Strengths | - Clear and concise - Good basic overview of evaluation with examples of specific projects in food safety - Good emphasis on showing causality and intended and unintended consequences - Useful references with links - Discusses ethics - Gives research contact at the FSA |
| Limitations | - Date of publishing could be clearer - Title does not make clear the subject of the document - Tone is very formal and dated - Could be more specific about intended target audience and their expected skills. - It is not detailed enough for beginners but too basic for those with experience. Its stated readership is Local Authorities but LAs will have evaluation experience and researchers and evaluators to offer support. - No mention of economic analyses though it does refer to the Magenta book. - Might be useful to place into the context of other government evaluation work particularly of Local Authorities – refers to the Magenta book and Green book but does not provide enough detail of these and they are both challenging reads for people with little evaluation experience. - Some of the approaches a bit dated and not necessarily accurate for example states that hiring an external person means that the evaluation is more objective which is not always the case and does not state the advantages of internal evaluation - Similarly states that a consultant is expensive and cheaper to get a research student or someone from a university. This is not objective advice. Many consultants are from universities and a full cost analyses may make external contractors cheaper, for example if they are more experienced or faster. This advice does not fit with research procurement processes in government - Should refer them to the research and evaluation support which most likely exists within the LAs and the experience that government has of commissioning research. |
| Link to the resource | <http://www.food.gov.uk/sites/default/files/Guidance%20-%20Introduction%20to%20evaluation%20for%20local%20authorities.pdf> |
| Links to related resources |  |
| Additional comments | Would be useful to have outlined the food standards agency’s responsibilities, though perhaps unnecessary for its intended audience.  Emphasis is on research rather than evaluation skills. |

| Title | Planning Evaluability Assessments: A synthesis of the literature with recommendations |
| --- | --- |
| Short title | Evaluability Assessment |
| Type of document/ accessibility | Online Pdf |
| Length | 58 |
| Lead author / organisation | Department For International Development commissioned Dr Rick Davis |
| Date | October 2013 |
| Source | Online |
| Purpose and utility of guidance | This is a synthesis of the literature in evaluability in projects in developing countries from 1979 to 2013. Evaluability is defined as “The extent to which an activity or project can be evaluated in a reliable and credible fashion.”  The purpose of this synthesis paper is to produce a practical report that summarises the literature on Evaluability Assessments and gives recommendations based on this. |
| Primary target audience | The primary audiences for the report are global evaluation advisers and development practitioners involved in commissioning and carrying out evaluations and Evaluability Assessments. |
| Contextual information | DFID is the UK Government’s Department for International Development. It works in developing countries to alleviate world poverty. The DFID produced this document to summarise the existing literature on evaluability assessments. It was intended to be a practical guide highlighting the main issues to be considered when conducting or commissioning an evaluability assessment. The document synthesizes 133 documents, and makes 18 recommendations about the use of Evaluability assessments. |
| Summary / overview | This is a synthesis of literature in evaluability (defined generally as the whether it Is possible to evaluate, and whether the information is available to do such an evaluation). In DFID these might be desk based projects taking up to 5 days or country based taking up to 2 weeks and should improve any subsequent evaluation.   - This sets out definitions of evaluability which vary widely. - Makes eighteen recommendations about how evaluability assessments should be used based on the synthesised literature. - States that an Evaluability Assessment should examine evaluability: (a) in principle, given the nature of the project design, and (b) in practice, given data availability to carry out an evaluation and the systems able to provide it. - It should also examine the likely usefulness of an evaluation. - The assessment should then affect the design of the evaluation and / or the design of the project. - An evaluability assessment should not be confused with an evaluation. - Many problems are related to weak project design and this can be addressed by engaging stakeholders at the beginning and evaluability assessments can support project designs. - An assessment can take place before a project is approved, to design the monitoring and evaluation framework, to decide whether the evaluation should take place, or to inform the specific design of an evaluation that has now been planned for. - Evaluability Assessments should be locally commissioned as these provide the most local support and ideally should be carried out by an independent third party. - Evaluability Assessments can offer good value for money, if they are able to influence the timing and design of subsequent evaluations. - Outputs of an Evaluability Assessment should include both assessments and recommendations. - The relatively low costs of Evaluability Assessments means that they only need to make modest improvements to an evaluation before their costs can be recovered. - The biggest risk of failure facing an Evaluability Assessment is likely to be excessive breadth of ambition. It should also be recognised that Evaluability Assessment may be seen as challenging, if there are already some doubts about a project design. |
| Strengths | - Involves several international aid agencies. - Detailed literature review. - Sets out recommendations at each stage. - Considers cost - Annexes which set out checklists, literature review methods etc. - Intended for a wider audience beyond DFID |
| Limitations | - Focused on international development (although could be used usefully by others). - Papers included since 1979 which suggests that many of these are likely to be out of synch with current developments in evaluation although the majority of papers were published in the later years. - Evaluability rather than evaluation |
| Link to the resources | <https://www.gov.uk/government/uploads/system/uploads/attachment_data/>  file/248656/wp40-planning-eval-assessments.pdf |
| Links to related resources |  |
| Additional comments | This is about evaluability – i.e. whether it is possible to evaluate a programme feasibly and whether the information to evaluate is there, practically rather than evaluation itself but it is still relevant to evaluation. |

| Title | The Magenta Book – Guidance for Evaluation |
| --- | --- |
| Short title | Magenta Book |
| Type of document/ accessibility | Online PDF, also available in paper form |
| Length | 141 |
| Lead author / organisation | HM Treasury |
| Date | 2011 |
| Source | Gov.UK |
| Purpose and utility of guidance | Use this document if you are reviewing or assessing a policy or project within government |
| Primary target audience | Analysts and policy makers at all levels of government, both central and local. The new guidance recognises evaluation’s place at the heart of policy development, and emphasises that the ability to obtain good evaluation evidence rests as much on the design and implementation of the policy as it does on the design of the evaluation. This gives policy makers much more of the responsibility for securing good evidence than was previously the case. |
| Contextual information | The Government is committed to improving central and local government efficiency and effectiveness, and in times of constrained public finances it is even more important to ensure that public funds are spent on activities that provide the greatest possible economic and social return. This requires that policy is based on reliable and robust evidence, and high quality evaluation is vital to this.HM Treasury’s Green and Magenta Books together provide detailed guidelines, for policy makers and analysts, on how policies and projects should be assessed and reviewed. The two sets of guidance are complementary: the Green Book emphasising the economic principles that should be applied to both appraisal and evaluation, and the Magenta Book providing in-depth guidance on how evaluation should be designed and undertaken. The Magenta Book is the recommended central government guidance on evaluation that sets out best practice for departments to follow.  It is intended to explain:   - The important issues and questions to consider in how evaluations should be designed and managed; - The wide range of evaluation options available; - Why evaluation improves policy making; - How evaluation results and evidence should be interpreted and presented; and, - Why thinking about evaluation before and during the policy design phase can help to improve the quality of evaluation results without needing to hinder the policy process. |
| Summary / overview | Part A is designed for policy makers. It sets out what evaluation is, and what the benefits of good evaluation are. It explains in simple terms the requirements for good evaluation, and some simple steps that policy makers can take to make a good evaluation of their intervention more feasible. It also discusses some of the issues around the interpretation and presentation of evaluation results, especially as they relate to the quality of the evaluation evidence.  Part B is aimed at analysts and interested policy makers and is therefore more technical. It discusses in greater detail the key steps to follow when planning and undertaking an evaluation and how to answer evaluation research questions using different evaluation research designs. It also discusses approaches to the interpretation and assimilation of evaluation evidence.  Includes:   - Policy evaluation –what it is, what benefits it can bring, what affects how a policy should be evaluated and where evaluation fits in the policy cycle. - Choosing the right kind of evaluation for a policy, measuring the process and the impact, assessing whether the benefits justified the costs and considering economic evaluation. - Building impact evaluation into policy design and the role of comparison groups in identifying the impact of a policy - Designing an evaluation, the stages, governance, quality control, level of resources and timing - The evaluation framework - Theory-based evaluation - Reviewing the existing evidence - Systematic review - Rapid evidence assessment - Meta-evaluation and meta-analysis - Making sense of existing and new evidence - Data collection- tools and ethical issues. - Process evaluation - action research and case studies - Evaluating implementation and delivery - Research methods - Empirical impact evaluation - Drawing together and reporting evaluation evidence - How evaluation evidence may be used - Drawing together the evaluation evidence, setting the evaluation results in a broader context - Future decisions and roll-out - Reporting and disseminating findings |
| Strengths | - Provides a good generic overview of what evaluation is and how to do it in relation to policy development - Regularly updated - Relatively simple to understand – does not assume much knowledge of evaluation - Discusses how things should be changed and developed as a result of an evaluation rather than ending at the dissemination stage. - Brings different aspects of evaluation needed for government projects into one area. - Focuses largely on how to conduct an evaluation to ensure monetary worth of policy |
| Limitations | - The book is intended for use by policy makers, thus its relevance to other public health practitioners is limited - Presents a simplistic overview of evaluations. A lot of the complexities associated with evaluating complex interventions are not mentioned or skimmed over. - Focuses largely on conducting evaluation to ensure monetary worth – rather than on identifying processes - Sees process evaluation (largely) as an assessment of fidelity and acceptability. |
| Link to the resources | <https://www.gov.uk/government/uploads/system/uploads/attachment_data/file/220542/magenta_book_combined.pdf> |
| Links to related resources | <https://www.gov.uk/government/uploads/system/uploads/attachment_data/file/220541/green_book_complete.pdf> |
| Additional comments | Complements the Green Book which focuses on economic appraisal |

| Title | Guidance on Evaluation and review for DFID (Department for International Development) staff |
| --- | --- |
| Short title | DFID Evaluation guide |
| Type of document/ accessibility | Online PDF |
| Length | 89 |
| Lead author / organisation | Gov.UK |
| Date | 2005 |
| Source | Gov.UK |
| Purpose and utility of guidance | To support DFID staff with designing, managing, reporting on, or responding to an evaluation. |
| Primary target audience | DFID staff |
| Contextual information | The department for International Development leads the UK’s work to end extreme poverty. The department is responsible for  •honouring the UK’s international commitments and taking action to achieve the Millennium Development Goals  •making British aid more effective by improving transparency, openness and value for money  •targeting British international development policy on economic growth and wealth creation  •improving the coherence and performance of British international development policy in fragile and conflict-affected countries  •improving the lives of girls and women through better education and a greater choice on family planning  •preventing violence against girls and women in the developing world  •helping to prevent climate change and encouraging adaptation and low-carbon growth in developing countries.  Agreement on a common agenda for development in the form of the UN Millennium Development Goals (MDGs) has encouraged the different stakeholders in the international development community to come together to support the main drivers of change in a more coherent way. These drivers of change are global and regional as much as national and involve work with many different stakeholders. Evaluating this level of development activity requires increasingly complex, multi-stakeholder, thematic evaluations and covers areas  such as:  ● conflict reduction and prevention  ● fair international trade  ● environment agreements  ● gender, human rights and democracy  ● a new international financial architecture  ● more effective working arrangements amongst the UN family of institutions and other global institutions  ● a stronger national and international civil society  ● country ownership and leadership of poverty reduction processes.  ● attempts to harmonise activities amongst donors |
| Summary / overview | Chapters 1-3 provide detail about evaluation in international development context. This includes the need for evaluation of DFID projects, what evaluation is, why they are conducted, types of evaluation (formative/summative/self-evaluation/participatory evaluation/process evaluation/program evaluation/sector/country etc).  Chapter 4 is for those about to plan or commission an evaluation, and provides a step by step guide to the evaluation process including; what kind of evaluation is needed, how can the evaluation improve development effectiveness, what do DFID’s corporate systems require; who needs to be involved; how should the evaluation be planned and evaluated; how can the likelihood of risks be reduced? Some of the trickier issues are discussed at the end of chapter 4. Chapter 5 discusses terms of reference, and chapter 6 discusses evaluation teams. Chapter 7 is about reporting (who, when, why, and how). Chapter 8 discusses how to use evaluation and share lessons – ensuring that findings and lessons are targeted to potential users. Finally, Chapter 9 describes different possible stakeholders and their roles (primary beneficiaries, evaluators, other users, implementers, commissions); highlighting the need to ensure that all roles are clear from the onset.  A list of definitions and standards are presented as an appendix. Standard international criteria on what should be covered in an evaluation includes: Relevance, Effectiveness, Efficiency, Impact, and Sustainability. It is suggested that these five measures are applied to every evaluation.  A glossary of standard evaluation terms are also provided. |
| Strengths | - Helpful overview of evaluation, and how to plan evaluations within a very specific context. - Useful for those who want some background to evaluation, those who are new to evaluation but are going to be commissioning or doing one and for those who are more experienced in evaluation. - Set within the International development policy context - Global perspective - Specific to those working in evaluation in international development rather than another general evaluation guide, - It also has a guide to standard evaluation definitions and terms - Set within country programmes and evaluations (countries were DFID working) - Some mention of economic effectiveness - Emphasis on results approach - Includes costing information and how this works within government procurement - Discusses ‘soft’ skills in evaluation – for example the pressure of being evaluated, the difficulty of travelling for the evaluator and what to do if sensitive issues emerge. - Sets out the skills needed by the evaluation team - Includes checklists at the end of each chapter for the evaluator to check his or her progress |
| Limitations | - The document is very targeted to evaluation of DFID strategies, which may limit its use for workers outside this department. - Whilst the document provides good detail on planning an evaluation, the document provides no information on how to complete the evaluation; and assumes an external evaluator will do this. |
| Link to the resources | <https://www.gov.uk/government/uploads/system/uploads/attachment_data/file/67851/guidance-evaluation.pdf> |
| Links to related resources |  |
| Additional comments | This guidance is probably too specific to DFID to be of use to anyone working outside this area.  There is a new updated version coming out in the near future although most of this is still relevant. |

| Title | The Green book: appraisal and evaluation in central government |
| --- | --- |
| Short title | The Green Book |
| Type of document/ accessibility | Online pdf |
| Length | 114 |
| Lead author / organisation | HM Treasury |
| Date | July 2011 updated (original 2003) |
| Source | https://www.gov.uk/government/publications/the-green-book-appraisal-and-evaluation-in-central-governent |
| Purpose and utility of guidance | To support public funds being spent on activities that give the greatest benefits. To appraise and evaluate past and present activities and to encourage a more long tern analytically robust approach to this field. |
| Primary target audience | All appraisers and evaluators: particularly useful for those working for or within government and focusing on an economic approach. Specifically anyone required to conduct a basic appraisal or evaluation of a policy, project or programme; and those seeking to expand their knowledge in this area. |
| Contextual information | HM Treasury is the financial and economic department of the UK government. It is responsible for allocating budgets, managing all government spending and planning future spend. |
| Summary / overview | - The purpose of the Green Book is to ensure that before a policy, programme or project is adopted two questions are answered: Are there better ways to achieve this objective? Are there better uses for these resources? - This guidance is designed to promote efficient policy development and resource allocation across Government by informing decision-making, and improving the alignment of department policies with government priorities. - It emphasises the need to take account of the wider social costs and benefits of proposals, and the need to ensure the proper use of public resources as well as considering equalities through identifying other possible approaches which may achieve similar results, where possible attributing monetary values to all impacts of any proposed policy, project and performing an assessment of the costs and benefits for relevant options. - It aims to make the appraisal process throughout government more consistent and transparent and is a guide for all departments and agencies and for any project of any size. - It describes how the economic, financial, social and environmental assessments of a policy, programme or project should be combined. - It contains information on how to conduct advanced appraisal and what the analytic foundation is and emphasises that appraisal should be planned for at the beginning of a new policy. - As well as supporting the assessment and evaluation of policy it should also be used to support decisions on use or disposal of existing assets and new or replacement of capital resources as well as major procurement decisions. - It contains a technical annex with more details of specific technical issues. |
| Strengths | - A thorough and comprehensive guide to support government spending to maximise benefit. - It considers the wider economic and social costs including such issues as social inequalities and discounting. - Extremely useful for those working for and with governments. |
| Limitations | - It does not include such information as engaging stakeholders and supporting change, nor does it aim to. - Although it claims it is for all assessors and evaluators and for all levels of programs it would need to be adapted if used outside government and in smaller programs and it may often be the case that the necessary information does not exist. - It is most useful for those who already have economic evaluation and general evaluation experience - it is unlikely that the guide could be used without such support. |
| Link to the resources | <https://www.gov.uk/government/uploads/system/uploads/attachment_data/file/220541/green_book_complete.pdf> |
| Links to related resources | https://www.gov.uk/government/publications/the-magenta-book |
| Additional comments | Best read in conjunction with magenta book. |

| Title | A Guide for First Nations on Evaluating Health Programs |
| --- | --- |
| Short title | First Nations Evaluation |
| Type of document/ accessibility | Online and pdf |
| Length | 27 |
| Lead author / organisation | Health Funding Arrangements Division, Program Policy Transfer Secretariat and Planning Directorate, First Nations and Inuit Health Branch FNIHB), Health Canada |
| Date | Archived on July 25^th^ 2013 |
| Source | Online |
| Purpose and utility of guidance | A basic evaluation guide for First Nations communities that are taking control of their health programs under the under the Department's Health Transfer Initiative. |
| Primary target audience | Those working in first nation community programs |
| Contextual information | Health Canada is the Federal department responsible for helping Canadians maintain and improve their health, while respecting individual choices and circumstances. It has a Department Transfer Initiative which allows first nation communities to take control of their own community health. This guide is provided within that context and aims to be a basic evaluation guide to help them to start evaluating their programs. |
| Summary / overview | - This guide gives basic background on program evaluation, defining what it is and why communities have to evaluate their programmes, It then links evaluation and programme management showing how evaluation can assess whether a programme is achieving what it has set out to achieve and how you can use lessons learned in one setting to another setting, - It outlines a community needs assessment and health plan and outlines the need to collect the correct data. - It summarises how to plan for program evaluation linking the overall program goal to the objectives, the activity, the indicator and to the data. - It describes how to prepare an evaluation plan, what to do to carry out an evaluation and what should happen after an evaluation. (dissemination, report to stakeholder and planning for change). - The appendix has a clear diagram of a logic model. |
| Strengths | - Clear and easy to read. - Uses relevant examples throughout to illustrate point. - Clear illustrative diagrams. - Gives quite detailed advice on choosing the evaluation questions, indicators and so on with relevant examples. - Could be used with someone with no evaluation experience. - Takes reader through the steps explaining in a simple way how to carry out an evaluation, what kind of questions you could ask, indicators and what data to collect to do this. |
| Limitations | - Basic - No discussion of cost or cost effective evaluation - No discussion of planning for change although it does mention that evaluation is an ongoing process and that change should occur if necessary as part of the results. - Aimed only at those working in community evaluation |
| Link to the resources | http://www.hc-sc.gc.ca/fniah-spnia/pubs/finance/_agree-accord/guide_eval_prog/index-eng.php |
| Links to related resources | There are other documents accessible from this web page which describe the health transfer initiative but these are not specifically related to evaluation. |
| Additional comments | States that it is aimed at First Canadians or Inuits but could be used by anyone working in community evaluation and in other countries. |

| Title | Evaluating community projects: A practical guide |
| --- | --- |
| Short title | JRF Community Evaluations |
| Type of document/ accessibility | Pdf online |
| Length | 12 pages |
| Lead author / organisation | Joseph Rowntree Foundation  Marilyn Taylor, Derrick Purdue, Mandy Wilson and Pete Wilde |
| Date | 2003 |
| Source | Online |
| Purpose and utility of guidance | To support community organisations to carry out evaluation |
| Primary target audience | Community and voluntary organisations which were part of the JRF neighbourhood programme in England and Wales but also for community projects in general |
| Contextual information | The JRF are an independent development and social research charity, supporting a programme of research and development projects in housing, social care and social policy |
| Summary / overview | These guidelines were initially developed as part of the JRF Neighbourhood Programme. This programme is made up of 20 community or voluntary organisations all wanting to exercise a more strategic influence in their neighbourhood. The guidelines were originally written to help these organisations evaluate their work. They provide step-by-step advice on how to evaluate a community project which will be of interest to a wider audience.  How to evaluate: a step-by-step approach  Step 1: Review the situation  Step 2: Gather evidence for the evaluation  Step 3: Analyse the evidence  Step 4: Make use of what you have found out  Step 5: Share your findings with others |
| Strengths | - Useful for community projects - Clear - Annex on explaining findings to funders - Emphasis on using the results |
| Limitations | - Now 12 years old - Basic guide - Lack of economic effectiveness information - Many similar guides |
| Link to the resources | http://www.jrf.org.uk/sites/files/jrf/1859354157.pdf |
| Links to related resources |  |
| Additional comments |  |

| Title | Developing and evaluating complex interventions: new guidance |
| --- | --- |
| Short title | MRC1 Framework |
| Type of document/ accessibility | Online Pdf |
| Length | 39 |
| Lead author / organisation | Medical Research Council (MRC) |
| Date | 2008 |
| Source | Online |
| Purpose and utility of guidance | Intended to help researchers choose and implement appropriate methods for evaluating complex interventions. |
| Primary target audience | Producers and users of research (researchers, research funders, journal editors, etc.) |
| Contextual information | The MRC supports research across the entire spectrum of medical sciences, in universities and hospitals, in the UK and Africa. The guidance was produced by researchers in the MRC Population Health Sciences Research Network. |
| Summary / overview | Guidance on the development, evaluation and implementation of complex interventions to improve health. Updates a previous MRC framework, extending it to non-experimental methods, and complex interventions outside the health service.  Questions for each stage:   - Developing an intervention - Clear about what you are trying to do, outcome aiming for, and how to bring about change? A coherent theoretical basis which is used to develop the intervention? Can you describe the intervention fully, sothat it can be implemented properly for the purposes of your evaluation, and replicated by others? Doesthe existing evidence suggest that it is likely to be effective orcost effective? Can it be implemented in a research setting, and is it likely to be widely implementable if theresults are favourable? If not then needs developed further. - Piloting and feasibility- Have you done enough piloting and feasibility work to be confident that theintervention can be delivered as intended? Can you make safe assumptions about effect sizes and variability,and rates of recruitment and retention in the main evaluation study? - Evaluating the intervention-What design are you going to use, and why? Is an experimental designpreferable and if so, is it feasible? Suggests alternative designs that might be better under different circumstances.Have you set upprocedures for monitoring delivery of the intervention, and overseeing the conduct of the evaluation and considered Process evaluation, economic evaluation. - Reporting- reported appropriatelyupdated yoursystematic review? Need both a detailed account of the intervention, as well as a standard report of the evaluation methods and findings, to enable replication studies, or wider scale implementation. - Implementation: Are your results accessible to decision-makers presented persuasively. Recommendations detailed and explicit? - Ongoing monitoring should be undertaken to detect adverse events or long term outcomes that could not be observed directly in the original evaluation, or to assess whether the effects observed in the study are replicated in routine |
| Strengths | - Excellent questions that researchers should ask themselves at the start of an evaluation - Good clear case studies of complex evaluations - Focus on complex interventions which reflect real world situations - Good for experienced researchers as provides technical support rather than an evaluation overview - Researchers often involved in evaluation but might not have evaluation skills however other evaluation guides too general for them. - Includes a case study on economic effectiveness - Wide consultation |
| Limitations | - Mainly aimed at researchers - Does not discuss evaluation theory - No qualitative description and a qualitative case study - Would be useful if the questions were presented as a separate document and laid out more clearly. |
| Link to the resources | http://www.mrc.ac.uk/documents/pdf/complex-interventions-guidance/ |
| Links to related resources | http://www.ncbi.nlm.nih.gov/pmc/articles/PMC2769032/  Related academic article; free full text available at [www.bmj.com/content/337/bmj.a1655](http://www.bmj.com/content/337/bmj.a1655) |
| Additional comments | Update of 2000 Framework |

| Title | Process Evaluation of Complex Interventions |
| --- | --- |
| Short title | MRC2 Process Evaluations |
| Type of document/ accessibility | Online Pdf |
| Length | 134 pages |
| Lead author / organisation | MRC Population Health Science Research Network |
| Date | No date? But follows a 2010 workshop |
| Source | online |
| Purpose and utility of guidance | This document provides researchers, practitioners, funders, journal editors and policy-makers with guidance in planning, designing, conducting and appraising process evaluations of complex interventions. Focuses on health but relevant to other domains |
| Primary target audience | Primarily researchers but also practitioners, funders, journal editors and policy-makers |
| Contextual information | MRC Population Health Science Research Network (PHSRN) was established by UK Medical Research Council in 2005 to focus on methodological knowledge transfer in population health sciences. |
| Summary / overview | Provides an overview of process evaluations divided into two sections (A) process evaluation theory and (B) process evaluation practice. Written from the perspective of researchers who have experience of process evaluation of complex health interventions.  It states why it is necessary, what it is, and how it should be done:  Why? Allows policy-makers, practitioners and researchers to identify which aspects of an interventions are effective at a fine grained level so that you can improve the aspects that are not effective. If an intervention is effective in one context, what does a policy-maker need to know that it will be effective in another and if it will produce the same outcomes? What were the mechanisms through which it was achieved and how can this vary by population and setting rather than looking at a straight forward outcome  If not is the failure due to the intervention itself or poor implementation. What information do systematic reviewers need to be confident that they are comparing interventions which were delivered in the same way; understand why the same intervention has different effects in different contexts?  What? Process evaluations aim to provide the more detailed understanding needed to inform policy and practice by examining implementation, the mechanisms of impact and the Context.  How? Process evaluations may be conducted within feasibility testing phases, alongside evaluations of effectiveness, or alongside post-evaluation scale-up. When designing and conducting a process evaluation, evaluators should describe the intervention and clarify causal assumption, identify key uncertainties and potential questions, agree scientific and policy priority, identify previous process evaluation, Select a combination of quantitative and qualitative methods and consider collecting this at multiple time points to capture change.  The guide includes information on:   - Analysing- Details on what is necessary when analysing process data - Reporting - How it should be reported, in line with exiting reporting guidelines, publishing journal articles and show how the findings can be widened into other areas. - How and when to share information with policy makers and practitioners - The Relationship between evaluation team - Resources and staffing - Defining the intervention and clarifying key assumptions - What do we know already? What will this study add? - Core aims and research questions - Selecting appropriate methods - Analyses of different methods - Integration of process evaluation and outcomes findings - Reporting findings of a process evaluation both to wider audience and academic journals. |
| Strengths | - Detailed description of process evaluation - Includes case studies - Theory and practice dealt with separately and users can find area most interested in. - Directs readers to sections which are most relevant for them - Focuses on a deeper understanding interventions and a more fine grained account of how they have an effect. - At a more complex level and suitable for experienced researchers– many evaluation guides are basic and aimed at beginners. - Good discussion of relationships which other guides do not go into. |
| Limitations | - No date - Not sure funders etc. would read such a complex and lengthy document (although there are subsequent and more simple guides from this) - Might be useful to draw out lessons for non- health audience and publish in relevant journals if not done so. |
| Link to the resources | http://decipher.uk.net/wp-content/uploads/2014/11/MRC-PHSRN-Process-evaluation-guidance.pdf |
| Links to related resources |  |
| Additional comments | Usefully read alongside other MRC evaluation documents |

| Title | Using natural experiments to evaluate population health interventions: guidance for producers and users of evidence |
| --- | --- |
| Short title | MRC3 Natural Experiments |
| Type of document/ accessibility | Online Pdf |
| Length | 29 |
| Lead author / organisation | Medical Research Council |
| Date | Not stated |
| Source | online |
| Purpose and utility of guidance | This guide aims to give general guidance on the range of approaches available for natural experiments in population health and the circumstances in which they are likely to be useful and to bring the methodological literature together which is currently dispersed across disciplines. |
| Primary target audience | Producers, users, funders and publishers of evidence |
| Contextual information | The MRC supports research across the entire spectrum of medical sciences in the UK and the MRC unit in Africa. This guide was produced by those working in population health. |
| Summary / overview | The guide describes the evaluation of interventions that were not under the control of the researchers (such as new policies) and methods of nonetheless drawing conclusions about their impact. The guide provides a detailed overview of what natural experiments are, a series of examples in public health (Section 2), a review of ways of improving the use of natural experiments and how this can be useful for policy makers (Section 3). Section 4 describes guidelines for improving design, analysis an reporting. It states that:   - Natural experiments are those which are not under control of researchers. They might occur naturally or be a result of interventions or policies. These can be useful for researchers as they can use variation in exposure to analyse impact. - In order to do this the variation in exposure and outcomes must be analysed using methods that attempt to make causal inferences, for example the effects of clean air legislation, indoor smoking bans or the effects of economic downturn. - The results of these analyses could lead to changes in policy or support policies in similar areas. - The case for a natural experimental study is strongest when (i) there is scientific uncertainty about the size or nature of the effects of the intervention or (ii) when for practical, political or ethical reasons, the intervention cannot be introduced as a true experiment. For example when comparing groups who have had different levels of exposure to something. - A ‘value of information’ analysis can help to make a convincing case for a natural experimental study, and economic evaluation may make the results more useful for decision-makers. - Randomised controlled trials and natural experimental studies are both subject to similar threats to validity. However it is more difficult to minimise bias in natural studies. Natural experiments can be used to study more subtle effects, so long as a suitable source of variation in exposure can be found, but the design and analysis become more challenging. - Care is required to minimise bias and different methods are outlined to help with this. - The plausibility of causal inferences should be tested. - There are guidelines which can be followed to support the use of natural experiments for example STROBE or TREND. These state that the approach should be clearly identified, the assignment, process and intervention described and the methods clearly stated. Bias should be reduced using both qualitative and quantitative methods. Wherever possible, the results should be compared with those of other evaluations of similar interventions, paying attention to any associations between effect sizes and variations in evaluation methods and intervention design, content and context. - Natural experimental approaches have taught us a great deal, for example the replacement of coal gas with natural gas and the effect of the smoking ban. - However planned experiments should not be discounted and often Randomised Control Trials will be the only way to test effects validly. - Priorities for the future are to build up experience of promising but lesser used methods, and to improve by, for example, including good routine data from population surveys and administrative data |
| Strengths | - Few of the other evaluation guides addressed natural experiments explicitly - Reflects real world of evaluation - Written by experts in the field. |
| Limitations | - Date of publication not clearly stated - UK focused - States that it is for policy makers and so on but it is written very much in the style of an academic report for a research audience. - Would be useful to have a plain English version |
| Link to the resources | http://www.behaviourworksaustralia.org/wp-content/uploads/2012/10/NaturalExperimentsGuidance_MRC-guidance.pdf |
| Links to related resources |  |
| Additional comments | Read in conjunction with other MRC evaluation guides from the same group |

| Title | The 2002 User-Friendly Handbook for Project Evaluation |
| --- | --- |
| Short title | NSF Project Evaluation |
| Type of document/ accessibility | Online Pdf |
| Length | 86 |
| Lead author / organisation | Directorate for Education and Human Resources: Division of Research,  Evaluation and Communication National Science Foundation |
| Date | 2002 |
| Source | Online |
| Purpose and utility of guidance | This handbook is aimed at those without evaluation knowledge or skills and aims to blend technical knowledge with common sense. |
| Primary target audience | Specifically written for National Science Foundation (NSF) and its stakeholders to provide managers with a basic guide for the evaluation of NSF’s educational programs. |
| Contextual information | The National Science Foundation (NSF) is an independent federal agency in the US which aims to "to promote the progress of science; to advance the national health, prosperity, and welfare; to secure the national defence." It funds a quarter of university science research in the US. |
| Summary / overview | This is a basic handbook of evaluation for those who have no experience in this area. In section one, it describes what evaluation is, types of evaluation and how evaluation differs from other data collection; in section two it goes on to describe the different steps of evaluation from developing a model and engaging stakeholders, onto developing measurable questions and objectives, choosing a design and methodological approach and choosing who will be included in the evaluation. In section three it goes on the describe issues with data collection including time, cost, rigour and mixed methods and concludes in section 4 by emphasising the importance of cultural issues in order to ensure that the evaluation is suitable for different groups and that the findings are used by these groups. |
| Strengths | - Clearly written - Good basic level introduction to research and evaluation, defining such terms as sampling and quantitative and qualitative research - Discusses the cost and time of using different approaches in evaluation - Discusses making evaluation culturally responsive and how this will help to ensure that the results are used. - Gives further sources of support - Has an appendix on how to find an evaluator |
| Limitations | - Could give more detail on planning for change - Evaluation theory has moved since 2002 - Could be clearer about the country in which it is published. - No consideration of cost effectiveness as part of evaluation of a programme - Combines basic research skills and basic evaluation skills but this is at such a basic level that someone with these skills would not be able to carry out research or evaluation alone. - Generally more focused on research skills thane evaluation skills - Not all evaluation require sampling people and doing interviews and questionnaires. Data can be available from other sources and systems can be evaluated without research participants. - Describes dissemination as the final stage and does not discuss implementation of change. |
| Link to the resources | http://www.nsf.gov/pubs/2002/nsf02057/nsf02057.pdf |
| Links to related resources |  |
| Additional comments | This handbook assumes no evaluation or research knowledge and much of it is concerned with giving its readers knowledge of basic research terms and skills. |

| Title | Evaluation Toolkit |
| --- | --- |
| Short title | APCRC |
| Type of document/ accessibility | Website |
| Length | Not Applicable |
| Lead author / organisation | National Health Service (NHS), Avon Primary Care Research Collaborative (APCRC) |
| Date | 2003-2015 |
| Source | NHS APCRC |
| Purpose and utility of guidance | To facilitate the use and creation of evidence by building in evaluation into the commissioning cycle. APRC support those working in commissioning and provide support, advice, guidance and training to commissioners, programme and project managers, and members of health integration teams who are planning to undertake an evaluation. This support is at all stages of the evaluation from design, delivery, dissemination and implementation of findings. They also work with researchers in evaluation research |
| Primary target audience | Specifically for commissioners in the Avon county – although some tools have wider applicability. |
| Contextual information | The Avon Primary Care Research Collaborative is responsible for primary care related research across the Bristol, North Somerset and South Gloucestershire area. This includes supporting service evaluation. The overall aims are to (i) build our portfolio of NHS-relevant research through close working with colleagues from local universities and (ii) to ensure that high quality evaluation is routinely considered as part of every commissioning cycle. |
| Summary / overview | The website starts with an overview of what evaluation is and why it is needed. A number of services are offered by the APCRC, and these are also listed.  A tab termed “evaluation methodology” provides an overview of the difference between qualitative and quantitative approaches, and the difference between monitoring and evaluation. Links are provided to PDF documents that discuss different designs, data collection, costing, commissioning an external evaluation, and writing and disseminating findings.  Training course in the local area/ at local institutes are listed.  The site presents a 10 page document entitled “Evaluation for commissioners – Tool kit.” This document includes:   - Top tips - Key questions - Evaluation process flow chart - Scoring grid to assess the level of evaluation required - Checklist for planning service evaluation - Decision tree for evaluation methods - Quality assurance criteria grid - Template for an evaluation protocol   Patient and public involvement is explained and encouraged. Links to organisations such as involve are provided.  The final tab is links to other organisations. This includes the UK evaluation society, charities evaluation services, and research methods knowledge base. |
| Strengths | - Clear description of the basic processes involved in evaluation - Useful and practical resources to support evaluation (i.e., checklists, flowcharts etc) - Provides support and links to academic establishments to support evaluation for those within the local area. - Provides advice on how to commission external evaluators - Provides bespoke evaluation training - Provides support in patient and public involvement - Gives links to other places where you can get evaluation support - It provides a tool to help people decide if their project is evaluation, audit or research. |
| Limitations | - Advice may be too simple to enable practitioners to conduct a comprehensive evaluation without additional support - Some services may only be available to people in the Avon area |
| Link to the resources | http://www.apcrc.nhs.uk/evaluation/toolkit.htm |
| Links to related resources |  |
| Additional comments |  |

| Title | LEAP for health. Learning, Evaluation and Planning |
| --- | --- |
| Short title | LEAP |
| Type of document/ accessibility | Online Pdf |
| Length | 86 |
| Lead author / organisation | NHS Health Scotland |
| Date | 2003 |
| Source | Online |
| Purpose and utility of guidance | A resource for those involved in promoting health and well-being in community settings, whether in community projects, primary care, clinical practice, health promotion or public health.  4 |
| Primary target audience | Those involved in promoting health and well-being in communities |
| Contextual information | NHS Health Scotland is a national Health Board working with public, private and third sectors to reduce health inequalities and improve health.  The LEAP approach is based on the assumption that agencies and workers should plan and evaluate their own work in partnership with one another and with those they aim to assist. All such people are referred to in this resource as stakeholders.  It emphasises self-evaluation, and is based on the assumption that evaluation should be an integral part of community health promotion; that providers and receivers should be involved; that the main aim should be for continual improvement; and lessons learned should inform future work.  The document criticises the traditional approach to evaluation as not focusing on local needs, emphasising money above all else, and giving too little attention to processes of change. The LEAP approach suggests that the approach to evaluation should be characterised by more attention being paid to the process of change, and to the outcomes that improve the quality of life. This is the approach to evaluation that they have adopted. It was developed and tested with community participants |
| Summary / overview | - The purpose of this resource is to: describe and explain the key components of the LEAP framework, set out criteria for planning and evaluating processes and tasks, discuss some of the issues involved in using the framework, provide information on methods and techniques for using the framework and consider how evaluation can inform planning, management /supervision, review and development. - Has a strong emphasis on the theoretical basis of evaluation and how this relates to practice - Its main aims are to enable those who use it to clarify what is involved in community health and well-being, and help stakeholders to plan their project, and evaluate and improve it focusing on the difference it had made to individuals and communities. - Sets out what community health and wellbeing is and states that this work focuses on enabling people to improve and maintain their health through community actions. - Highlights the policy context though does not refer to specific policy. - Focuses on the importance of improving outcomes and doing this by involving people, using evidence and learning from experience. - Outlines different approaches to evaluation and how to plan this into programmes at whatever level required. - Emphasises community health and well-being by involving people, building networks and strengthening communities and sets out what barriers there might be to participation. - Describes the five stage of leap: what needs to change, how will we know, how will we go about it, how will we know we did it, did we do it and was it useful, how do we review and improve. - Supports reader to identify indicators and to focus on outcomes that show improvement. - It provides action planning tables, worksheets and worked examples and practice notes as well as reading and resources. |
| Strengths | - Step by step guide which is clear and easy to read - Simple and clear approach to determining what a project is doing and if it is working - Focuses on community health - Discusses the asset based approach of community improvement - LEAP emphasises the importance of processes as well as outcomes - The importance of stakeholder involvement is strongly emphasised - The focus is on community improvement – rather than on financial outputs. - Aims to provide evidence on how a community intervention has had a positive effect. - Provides worked examples - References further reading and resources. - Resource developed and tested with community participants |
| Limitations | - NHS Scotland focused though could be used by others in community health - No mention of economic evaluation or cost effectiveness - No mention of comparing projects. - Limited to evaluation of community projects - The document provides a very clear definition of community health and wellbeing – the document may be of limited use for those working outside that definition - Could provide more details on relevant policies. |
| Link to the resources | <http://www.healthscotland.com/uploads/documents/308-LEAP_for_health.pdf>  [**http://www.planandevaluate.com/**](http://www.planandevaluate.com/) |
| Links to related resources | http://www.scdc.org.uk/what/LEAP |
| Additional comments |  |

| Title | Mental health improvement: Evidence and practice |
| --- | --- |
| Short title | Health Scotland Mental Health Improvement |
| Type of document/ accessibility | Online Pdf |
| Length | 32 |
| Lead author / organisation | NHS Health Scotland |
| Date | 1^st^ March 2005 |
| Source | online |
| Purpose and utility of guidance | This is a series of four guides which aim to encourage, support and improve standards in the evaluation of mental health improvement initiative:  Evidence based practice  Measuring success  Getting results  Making an impact. |
| Primary target audience | It is aimed specifically at those working in the mental health field who are doing evaluations to support them to use evidence to inform the design and delivery of interventions. |
| Contextual information | Health Scotland are the health improvement agency for Scotland and compiled this on behalf of the National Programme for Improving Mental Health and Well-being |
| Summary / overview | - The first of the four guides provides information on how existing literature can be used to inform the design and delivery of interventions. It considers issues around evidence, and how evidence can be used to inform intervention development. - The second guide discusses how to develop indicators to measure progress. This includes how to develop indicators that are robust, valid and reliable, and also provides links to existing indicators. - The third guide, getting results, is about planning and implementing an evaluation. It provides an overview of the key stages involved in planning, implementing an evaluation. It includes involving stakeholders, agreeing objectives, choosing methods, collecting data, and implementation issues. Links to further sources of information are also provided. - The final guide is making an impact. This provides details on how to analyse and interpret data, communicate findings, and add to the evidence base. |
| Strengths | - A good overview of the area - Illustrates theory with useful examples - Clear and easy to read - Provides links to resources - Provides a glossary of terms - Available in different formats |
| Limitations | - Possibly too simple to use alone - Specifically for mental health - Specifically for the Scottish context – although could be used in other contexts - It is likely that this field has developed in the ten years since this was published. - For those with some knowledge of evaluation and mental health |
| Link to the resources | <http://www.healthscotland.com/uploads/documents/2467-Guide1Evidencebased.pdf>  <http://www.healthscotland.com/uploads/documents/458-Guide2Measuringsuccess.pdf>  <http://www.healthscotland.com/uploads/documents/459-Guide3Gettingresults.pdf>  <http://www.healthscotland.com/uploads/documents/460-Guide4Makinganimpact.pdf> |
| Links to related resources |  |
| Additional comments |  |

| Title | NHS Scotland: Evaluation |
| --- | --- |
| Short title | NHS Scotland |
| Type of document/ accessibility | Website |
| Length | NA |
| Lead author / organisation | NHS Scotland |
| Date | Current |
| Source | NHS Scotland |
| Purpose and utility of guidance | To support NHS workers with support in outcome planning and evaluation processes |
| Primary target audience | Local partners in the NHS |
| Contextual information | The team evaluate Scotland's policies and programs intended to improve health and reduce health inequalities to generate a better understanding of how they work, who they reach, and what effects they have. |
| Summary / overview | The website provides links to the Outcomes Framework Website and further resources.  The outcomes Framework provides interactive resources to help link activities to outcomes for a number of areas (reducing alcohol related harm, tobacco control, mental health improvement, health and work, healthy weight, and national parenting strategy). Frameworks include a series of tools: Outcomes triangle, Logic models, and results chains. |
| Strengths | - Practical resources are provided to support evaluators identify and evaluate links between what is done and what is achieved. - Useful for those who are interested in evaluating a program on reducing alcohol related harm, tobacco control, mental health improvement, health and work, healthy weight, and national parenting strategy. - Simple to understand - Interactive - A glossary is provided |
| Limitations | - Focus is on processes rather than evaluation as a whole - Only useful to those areas covered |
| Link to the resources | <http://www.healthscotland.com/scotlands-health/evaluation/planning/hi-performancemanagement-nhs.aspx> |
| Links to related resources |  |
| Additional comments |  |

| Title | Behaviour change: the principles for effective interventions |
| --- | --- |
| Short title | NIHR |
| Lead author / organisation | National Institute for Health and Care Excellence |
| Date | October 2007 |
| Source | National Institute for Health and Care Excellence |
| Purpose and utility of guidance | This guidance provides a set of generic principles that can be used as the basis for planning, delivering and evaluating public health activities aimed at changing health-related behaviours. |
| Primary target audience | The guidance is for NHS and non-NHS professionals and others who have a direct or indirect role in, and responsibility for, helping people change their health-related knowledge, attitudes and behaviour. This includes national policy makers in health and related sectors (including those with a responsibility for planning or commissioning media, marketing or other campaigns), and commissioners, providers and practitioners in the NHS, local government, the community and voluntary sectors. It is also relevant for the research community (including those who oversee research funding), social and behavioural scientists, and health economists working in the area of health-related knowledge, attitude and behaviour change. |
| Contextual information | The National Institute for Health and Care Excellence (NICE) are a UK government agency which provides national guidance and advice to improve health and social care.  The guide was developed to fill a void in that, prior to this guide, there no strategic approach to behaviour change across government, the NHS or other sectors, and many different models, methods and theories were used in an uncoordinated way.  This guidance provides a systematic, coherent and evidence-based approach, considering generic principles for changing people's health-related knowledge, attitudes and behaviour, at individual, community and population levels. |
| Summary / overview | This document is the Institute's formal guidance on generic principles that should be used as the basis of initiatives to support attitude and behaviour change.  It is divided in different sections describing recommended actions for the 1) Planning, 2) Delivery, 3) Evaluation, and 4) Implementation of interventions to change behaviour. It further includes 5) Recommendations for research.  It includes:   - Background on health inequalities and changing behaviour. - Overview of what is required in planning an intervention for behaviour change and this is also required for evaluation. - Emphasises the need to work with the community to ensure an intervention will work in that setting and to avoid stigmatising behaviour.   Focusing on the small part of the document which is specifically concerned with evaluation, the guide states that:   - Time and resources should be set aside for evaluation. - The size and nature of the intervention, its aims and objectives and the underlying theory of change used should determine the form of evaluation - Distinguishes between monitoring and evaluation - States that complex interventions can be evaluated if a staged approach is used - Emphasises the need for formal outcome and process evaluation despite how challenging this can be. - An effective evaluation is based on clearly defined outcome measures – at individual, community and population levels, as appropriate. - Qualitative research looking at the experience, meaning and value of changes to individuals may also be appropriate. Methods and outcome measures are identified during the planning phase. - Needs to have a theory. |
| Strengths | - Rather than focussing on a specific type of public health intervention, it focusses on how to change health-related behaviours of people using individual-, community- or population-level interventions. - Specific highlighted set of key theories is provided on which the recommendations are based, with abundant references for people who want to explore these. - Very clear outline in which every chapter, in a bullet point-type approach described the target audience and recommended actions for different type of interventions |
| Limitations | - It’s a relatively short document, which is an advantage, but at the same time describes generic recommendations only. - Little of this is focused on evaluation - Difficult for one document to meet the needs of such a wide audience - Is not a stand-alone guide, but needs to be supplemented with more specific/detailed reading. |
| Link to the resources | <http://www.nice.org.uk/guidance/ph6/resources/guidance-behaviour-change-the-principles-for-effective-interventions-pdf> |
| Links to related resources | Weiss CH (1995) Nothing as practical as good theory: exploring theory-based evaluation for comprehensive community initiatives for children and families. In Connell JP, Kubisch A, Schorr LB et al. editors. New approaches to evaluating community initiatives: concepts, methods and context. Washington DC: Aspen Institute.   - <http://www.euro.who.int/en/media-centre/events/events/2008/06/who-european-ministerial-conference-on-health-systems/documentation/background-documents/seventh-futures-forum-on-unpopular-decisions-in-public-health> - <http://www.nice.org.uk/about> - <http://www.nice.org.uk/guidance/PH6> |
| Additional comments |  |

| Title | Evaluation of weight management, physical activity and dietary interventions: an introductory guide |
| --- | --- |
| Short title | PHE1 Introduction |
| Type of document/ accessibility | Online Pdf |
| Length | 35 |
| Lead author / organisation | NHS National Observatory on Obesity. Public Health England |
| Date | 2015 |
| Source | Public Health England |
| Purpose and utility of guidance | This guide provides a general introduction to the evaluation of public health programs. It is described as a useful first step for anyone new to the topic or those intending to refresh their knowledge. It is intended to be used alongside the standard evaluation frameworks (below); which detail the information and data that should be collected. |
| Primary target audience | Primarily for practitioners interested in evaluation of physical activity, weight management and dietary programs. |
| Contextual information | Public Health England aims to improve the health and wellbeing of the nation, and reduce health inequalities.  This document follows the publication of three standard evaluation frameworks for weight management interventions. It is intended to provide an overview of evaluation which compliments the finer detail provided in the frameworks. |
| Summary / overview | This short document provides an overview of evaluation – including:   1. What is evaluation and why is it important 2. Evaluation questions 3. An overview of process and outcome evaluations 4. Where to seek help 5. A step by step guide to evaluation – the evaluation cycle. This outlines the 6 main phases of evaluation:  - Planning – including stakeholder involvement and budgeting for an evaluation - Clarifying objectives – including the development of logic models - Selecting indicators – including process, short, medium and long term indicators - choosing methods and data collection – including formative, process and outcome evaluation. Data collection methods including qualitative and quantitative data, and ethics are discussed. - Analysing data - Reflecting and sharing – including dissemination approaches  1. An evaluation checklist 2. Further reading |
| Strengths | - Provides an excellent introduction to evaluation - Simple and clear to use - Covers all main elements involved in Evaluation - Practical and relevant to real world settings - Provides links to checklists and standard frameworks for dietary interventions - Provides links to further reading |
| Limitations | - Intended to be used by those working in obesity |
| Link to the resources | http://www.noo.org.uk/securefiles/150803_1319//EvaluationIntroductory.pdf |
| Links to related resources |  |
| Additional comments |  |

| Title | Collection of Resources on Evaluation - CoRE |
| --- | --- |
| Short title | PHE2 Resources |
| Type of document/ accessibility | Webpage |
| Length | Not applicable |
| Lead author / organisation | National Obesity Observatory (now part of Public Health England) |
| Date | Ongoing |
| Source | Online |
| Purpose and utility of guidance | To provide information and resources to support practitioners with an interest in the evaluation of interventions related to obesity, overweight, underweight and their determinants |
| Primary target audience | Policy makers and practitioners involved in obesity and related issues. |
| Contextual information | The aim of the National Obesity Observatory are to signpost and report on obesity and related surveillance data  The aim of these series of guides is to ensure results of interventions are comparable across settings, populations and types of intervention. This means that public health commissioning can be carried out more effectively, which is important in a time of restricted public finances.  The current version of CoRE covers: NOO’s Standard Evaluation Frameworks (SEF); evaluation data collection tool (including details of local interventions); other evaluation guidance; reports from evaluation of nationally-initiated schemes; and evaluation websites.  A link is provided to the Obesity Learning Centre for more resources on evaluation.  The National Obesity Observatory (NOO) is now part of Public Health England (PHE), an executive agency of the Department of Health. The Public Health England Obesity website [www.noo.org](http://www.noo.org/) provides a single point of contact for wide-ranging authoritative information on data, evaluation, evidence and research related to weight status and its determinants. PHE have developed three standard evaluation frameworks for weight management interventions, physical activity and dietary interventions.  PHE also have a collection of resources on evaluation (CoRE) including three Standard Evaluation Frameworks (SEF); evaluation data collection tool (including details of local interventions); other evaluation guidance; reports from evaluation of nationally-initiated schemes; and evaluation websites. This document builds on the ‘Standard Evaluation Framework (SEF) for weight management interventions’, published in April 2009. It takes the principles described in the original **SEF and applies them to dietary interventions.** |
| Summary / overview | CoRE is divided into 7 sections:   - [Standard Evaluation Frameworks](http://www.noo.org.uk/core/frameworks) - [Evaluation data collection tool](http://www.noo.org.uk/core/eval_collection) - [Database of interventions](http://www.noo.org.uk/sefsearch.php) - [Evaluation guidance](http://www.noo.org.uk/core/eval_guidance) - [Evaluation reports](http://www.noo.org.uk/core/eval_reports) - [Evaluation websites](http://www.noo.org.uk/core/eval_websites) - Evaluation training   The ‘Standard Evaluation Frameworks’ section notes 3 SEFs: Weight Management Interventions (2009); PA Interventions (2012); dietary interventions (2012). The aim of the SEF is to support high quality, consistent evaluation of weight management, diet and PA interventions for the evidence base. The SEFs provide introductory guidance on the principles of evaluation, list/describe ‘essential’ and ‘desirable’ criteria, provides information on collecting data and identifies the target audience. A link is given to a poster presented at the Public Health England Conference 2013.  The ‘Evaluation data collection tool’ section describes what the tool is for; why it should be used; type of intervention to be included; what happens to information provided; what sort of tool it is; Details on accessing and using the tool. There is also a link to obesity case study examples.  The ‘[Database of interventions](http://www.noo.org.uk/sefsearch.php)’ section is the first national database for weight management, diet and PA interventions, providing information on programmes that have been submitted via the NOO evaluation [data collection tool](http://www.noo.org.uk/core/eval_collection) since 2011. It has 4 dropdown menus: Geography; Intervention setting; Age group; Included Interventions. Links are provided to obesity case study examples; and information on obesity interventions in Ireland and Northern Ireland.  ‘The Evaluation Guidance’ section provides information/tools for planning the evaluation of interventions that, either directly or indirectly, prevent or reduce obesity. Nine are listed, with online links: ‘Framework of outcome measures recommended for use in the evaluation of childhood obesity treatment interventions: the CoOR framework’; ‘National Obesity Observatory, 2012. Standard Evaluation Framework for dietary interventions;’ ‘National Obesity Observatory, 2012. Standard Evaluation Framework for physical activity interventions’; ‘National Obesity Observatory; 2009. Standard Evaluation Framework for weight management interventions’; ‘National Obesity Observatory 2011. Evaluation data collection tool’; ‘National Heart Forum; 2008. Tool D14 Monitoring and Evaluation in ‘A Framework in ‘Healthy Weight Healthy Lives: A Toolkit for Developing Local Strategies’; ‘Medical Research Council; 2008 Developing and evaluating complex interventions: new guidance; ’Department of Health; 2005. Research governance framework for health and social care: Second edition’; ’Centers for Disease Control and Prevention, 1999. Framework for program evaluation in public health’.  Next, ‘Evaluation Reports’ has links to reports on the findings from the evaluation of nationally led programmes that influence obesity or its determinants grouped under headings:   - Behaviour change - Physical activity - Healthy eating - Healthy children and families - Health inequalities/healthy communities   Links are also given to other evaluation reports, case studies and intervention, to be found on PHE CoRE pages.  The ‘Evaluation websites’ section contains information on the evaluation of public health programmes from: NHS Scotland; US Centre for Disease Control (CDC) Evaluation Working Group Resources; National Institute for Health and Clinical Excellence (NICE);  UK Clinical Research Collaboration (CRC) Public Health Centres of Excellence; Social and Public Health Sciences Unit: Evaluating the Health Effects of Social Interventions. Links to webpages are provided.   - The ‘Evaluation Training’ section identifies 3 training sessions in understanding evaluation in public health, on weight management, PA, and dietary interventions (2014). Links are provided for more information |
| Strengths | - Clear, well laid out - Has a section where readers can search for interventions by target age group, geographical area, type of intervention - Links to frameworks data collection tool, guidance, reports, websites training, further information |
| Limitations | - Would be useful in mentioned obesity in title - Specific to obesity - UK focused |
| Link to the resources | <http://www.noo.org.uk/core> |
| Links to related resources | http://www.obesitylearningcentre-nhf.org.uk/  https://twitter.com/PHE_obesity  http://www.noo.org.uk/slide_sets  http://www.obesitylearningcentre.org.uk  http://www.noo.org.uk/NOO_pub/KU  http://www.noo.org.uk/signup.php |
| Additional comments | This site aims to collect resources together for the reader to search rather than providing these resources. |

| Title | Standard Evaluation Framework for dietary interventions |
| --- | --- |
| Short title | PHE3 Dietary interventions |
| Type of document/ accessibility | Online Pdf |
| Length | 40 |
| Lead author / organisation | NHS National observatory on obesity |
| Date | September 2012 |
| Source | Online |
| Purpose and utility of guidance | The SEF for dietary interventions aims to describe and explain the information that should be collected in any evaluation of an intervention that aims to improve dietary intake or associated behaviour |
| Primary target audience | The target audiences for this document are:  • commissioners or managers of weight management interventions with a dietary component  • commissioners or managers of dietary interventions  • obesity and diet leads in local authorities  • practitioners running weight management interventions with a dietary component  • evaluators of dietary interventions or weight management interventions with a dietary element. |
| Contextual information | The National Obesity Observatory (NOO) was established to provide a single point of contact for wide-ranging authoritative information on data and evidence related to obesity, overweight, underweight and their determinants. The NOO have developed three standard evaluation frameworks for weight management interventions, physical activity and dietary interventions. The NOO also have a collection of resources on evaluation (CoRE) including three Standard Evaluation Frameworks (SEF); evaluation data collection tool (including details of local interventions); other evaluation guidance; reports from evaluation of nationally-initiated schemes; and evaluation websites. This document builds on the ‘Standard Evaluation Framework (SEF) for weight management interventions’,  published by NOO in April 2009. It takes the principles described in the original SEF and applies them to dietary interventions.  The National Obesity Observatory (NOO) is now part of Public Health England (PHE), an executive agency of the Department of Health. The Public Health England Obesity website [www.noo.org](http://www.noo.org/) provides a single point of contact for wide-ranging authoritative information on data, evaluation, evidence and research related to weight status and its determinants. PHE have developed three standard evaluation frameworks for weight management interventions, physical activity and dietary interventions.  PHE also have a collection of resources on evaluation (CoRE) including three Standard Evaluation Frameworks (SEF); evaluation data collection tool (including details of local interventions); other evaluation guidance; reports from evaluation of nationally-initiated schemes; and evaluation websites. This document builds on the ‘Standard Evaluation Framework (SEF) for weight management interventions’, published in April 2009. It takes the principles described in the original SEF and applies them to dietary interventions. |
| Summary / overview | The aim of the Standard Evaluation Frameworks (SEF) is to support high quality, consistent evaluation of weight management, diet and physical activity interventions in order to increase the evidence base. The guidance document’s ‘Introduction’ (Section 1) identifies the aims of the document, and discusses why a dietary SEF is necessary. Section 2 on ‘Principles of evaluation’ identifies 2 core evaluation questions: what are the objectives? How will they be measured? It then differentiates between primary and secondary outcome measures, illustrating with example projects/objectives, and discusses the utility of ‘logic models’ to identify primary and secondary measurement indicators. Section 3 details selecting and measuring outcomes: identifies factors important when choosing an outcome indicator; identifies four main categories of outcome when dietary intake is the primary outcome: i) intake of a particular food or food group, ii) intake of a particular nutrient, iii) overall energy intake, iv) meeting of dietary recommendations. It then discusses different options for measuring dietary intake. Different methods to measure outcomes are described with pros and cons identified. Potential sources of error and bias are outlined and further resources/toolkits on dietary assessment and measurement are identified. A flow chart illustrates the process to be followed when planning an intervention. Section 4 sets out the SEF and presents criteria necessary to undertake a comprehensive and robust dietary evaluation. Fifty-two essential and desirable criteria are listed. Essential criteria are presented as the minimum recommended data for evaluation purposes. Desirable criteria are additional data that would enhance the evaluation. For example, it is essential to have data on the primary and secondary aims and objectives, intervention timescales, and descriptions of the intervention. Desirable criteria include a rationale for the intervention (including theoretical basis and logic model) and duration of funding. The 52 criteria relate to each stage of the evaluation from title/name of the evaluation to dissemination of the findings. Explanatory notes (section 5) are accessed by clicking on the links to the interactive document. |
| Strengths | - Very accessible: interactive, includes visuals e.g. flow charts, 40 pages. - Clear about purpose and target audience - Clearly written and takes reader through each stage with examples - Includes information on collecting the cost of an intervention as well as the resources and skills required - Provides evaluation support to a wide range of professionals involved in dietary interventions. It is intended to increase the number and quality of evaluations of such programs; - may contribute to the development of a core dataset that would increase the comparability of evaluations; - gives a basic overview of evaluation so users have a minimum understanding prior to using the framework; - provides a checklist distinguishing essential and desirable criteria. This allows users to prioritise when resources are scarce; - suited to a range of dietary interventions e.g. individual and group based; - a wide range of stakeholders including practitioners and academics were heavily involved in the development; - includes a helpful glossary of terms; - Includes policy context - Focus on sustainability - Discusses generalisability |
| Limitations | - Checklist approaches may be too simplistic for complex interventions, specific situations may need to adapt essential and desirable criteria; - specific to interventions for dietary intake; - offers a basic overview of evaluation that may be insufficient for users to conduct high quality evaluations with no additional help; - members of the public were not involved in development of the SEF; - bias: does not identify the potential for professional bias e.g. when constructing or asking survey/interview questions. - essential/desirable process criteria does not include ‘participant motivation’ to take part in (opt-in) the intervention (opt-out data is essential); - need for qualitative guidance not identified; - qualitative research definition in the glossary limited. - UK / England focused - It does not provide an introduction to evaluation but refers to where one can be found - It is aimed at interventions that work at individual or group level, not at population level. - Focuses on food guidelines |
| Link to the resources | http://www.noo.org.uk/uploads/doc/vid_16724_SEF_Diet.pdf |
| Links to related resources | www.noo.org.uk/core  [www.noo.org.uk](http://www.noo.org.uk) |
| Additional comments | Read alongside the other Public Health England documents on evaluation particularly the SEF on physical activity |

| Title | Standard Evaluation Framework for physical activity interventions |
| --- | --- |
| Short title | PHE4 Physical Activity Interventions |
| Type of document/ accessibility | Online Pdf |
| Length | 39 pages |
| Lead author / organisation | NHS National Obesity Observatory (NOO) |
| Date | September 2012 |
| Source | Online |
| Purpose and utility of guidance | Builds on the *Standard Evaluation Framework (SEF) for weight management interventions*, published by NOO in April 2009, applying these principles to physical activity interventions. The SEF contains a list of ‘essential’ and ‘desirable’ criteria for data required for a comprehensive and robust evaluation. |
| Primary target audience | The target audiences for this document are:  • commissioners or managers of weight management or obesity prevention interventions with a physical activity element  • commissioners or managers of physical activity interventions  • physical activity or sport and leisure leads in local authorities  • commissioners or managers of active travel projects  • practitioners running physical activity or active travel projects. |
| Contextual information | The National Obesity Observatory (NOO) was established to provide a single point of contact for wide-ranging authoritative information on data and evidence related to obesity, overweight, underweight and their determinants. The NOO have developed three standard evaluation frameworks for: weight management interventions, physical activity interventions; and dietary interventions. The NOO also have a collection of resources on evaluation (CoRE) including three Standard Evaluation Frameworks (SEF); evaluation data collection tool (including details of local interventions); other evaluation guidance; reports from evaluation of nationally-initiated schemes; and evaluation websites. This document builds on the ‘Standard Evaluation Framework (SEF) for weight management interventions,’ published by NOO in April 2009. It takes the principles described in the original SEF and applies them to physical activity interventions.  The National Obesity Observatory (NOO) is now part of Public Health England (PHE), an executive agency of the Department of Health. The Public Health England Obesity website [www.noo.org](http://www.noo.org/) provides a single point of contact for wide-ranging authoritative information on data, evaluation, evidence and research related to weight status and its determinants. PHE have developed three standard evaluation frameworks for weight management interventions, physical activity and dietary interventions.  PHE also have a collection of resources on evaluation (CoRE) including three Standard Evaluation Frameworks (SEF); evaluation data collection tool (including details of local interventions); other evaluation guidance; reports from evaluation of nationally-initiated schemes; and evaluation websites. This document builds on the ‘Standard Evaluation Framework (SEF) for weight management interventions’, published in April 2009. It takes the principles described in the original SEF and applies them to dietary interventions. |
| Summary / overview | The aim of the Standard Evaluation Frameworks (SEF) is to support high quality, consistent evaluation of weight management, diet and physical activity interventions in order to increase the evidence base.  The guidance document’s ‘Introduction’ (Section 1) identifies the aims of the document, defines physical activity and discusses why a SEF in physical activity is necessary. Section 2 on ‘Principles of evaluation’ identifies 2 core evaluation questions: what are the objectives and how will they be measured? It then differentiates between primary and secondary outcome measures, illustrating with example projects/objectives, and discusses the utility of ‘logic models’ to identify primary and secondary measurement indicators. Section 3 details selecting and measuring outcomes; identifies four main ways to classify physical activity: frequency, intensity, time, type (FITT) and discusses different options for measuring physical activity. Potential sources of error and bias are outlined, then existing datasets for evaluation purposes are considered and further resources (tools) on physical activity assessment are identified. A flow chart illustrates the process to be followed when planning an intervention. Section 4 sets out the SEF and presents criteria necessary to undertake a comprehensive and robust evaluation. Fifty-two essential and desirable criteria are listed. Essential criteria are presented as the minimum recommended data for evaluation purposes. Desirable criteria are additional data that would enhance the evaluation. For example, it is essential to have data on the primary and secondary aims and objectives, intervention timescales, and descriptions of the intervention. Desirable criteria include, a rationale for the intervention (including theoretical basis and logic model) and duration of funding. The 52 criteria relate to each stage of the evaluation – from title/name of the intervention to analysis and interpretation. Explanatory notes (Section 5) are accessed by clicking on the links in the interactive document. |
| Strengths | - provides evaluation support to a wide range of professionals involved in planning and evaluating physical activity projects and interventions. It is intended to increase the number and quality of evaluations of such programs; - may also contribute to the development of a core dataset that would increase the comparability of evaluations; - gives a basic overview of evaluation so users have a minimum understanding prior to using the framework; - provides a useful checklist distinguishing essential and desirable criteria. This allows users to prioritise when resources are scarce; - suited to a range of physical activity interventions; including individual and group based interventions; - A wide range of stakeholders including practitioners and academics were heavily involved in the development; - includes a helpful glossary of terms - accessible e.g. interactive, includes visuals such as flow charts and is 39 pages long. - Clear about purpose and target audience - Clearly written and takes reader through each stage with examples - Contains rapid review of physical activity measurements - Includes information on collecting the cost of an intervention as well as the resources and skills required - Includes policy context - Focus on sustainability - Discusses generalisability |
| Limitations | - UK / England focused - It does not provide an introduction to evaluation but refers to where one can be found - Focuses on physical activity programmes. - It is aimed at interventions that work at individual or group level, not at population level, for example those programmes which involve changing the physical environment. - Checklist approaches may be too simplistic for the nuances of complex interventions. Individual situations may require modifications to the essential and desirable criteria. - The framework is specific to interventions for physical activity. - The document offers a very basic overview of evaluation. This may be insufficient for practitioners to conduct high quality evaluations without additional help. - Members of the public were not involved in the development of the framework. - The document does not provide an introduction to the concepts of evaluation. - The section on potential bias refers to ‘respondent’ bias and does not identify the potential for professional bias e.g. when constructing or asking survey/interview questions. - Essential/desirable process criteria does not include ‘participant motivation’ to take part in (opt-in) the intervention (whereas opt-out data is essential); - Does not identify need for qualitative guidance; - Qualitative research definition in the glossary is limited. |
| Link to the resources | <http://www.noo.org.uk/uploads/doc/vid_16722_SEF_PA.pdf> |
| Links to related resources |  |
| Additional comments | Accompanies the dietary guideline.  Should be read alongside other evaluation work from NOO |

| Title | Standard evaluation framework for weight management interventions |
| --- | --- |
| Short title | PHE5 Weight management interventions |
| Type of document/ accessibility | Online PDF |
| Length | 60 pages |
| Lead author / organisation | National Obesity Observatory, Public Health England |
| Date | 2009 |
| Source | Public Health England |
| Purpose and utility of guidance | This document should be used when planning an evaluation of a weight management intervention. |
| Primary target audience | - Local authorities and clinical commissioning groups - Other organisations running weight management interventions - Evaluators |
| Contextual information | The National Obesity Observatory (NOO) was established to provide a single point of contact for wide-ranging authoritative information on data and evidence related to obesity, overweight, underweight and their determinants. The NOO have developed three standard evaluation frameworks for weight management interventions, physical activity and dietary interventions. The NOO also have a collection of resources on evaluation (CoRE); evaluation data collection tool (including details of local interventions); other evaluation guidance; reports from evaluation of nationally-initiated schemes; and evaluation websites.  The National Obesity Observatory (NOO) is now part of Public Health England (PHE), an executive agency of the Department of Health. The Public Health England Obesity website [www.noo.org](http://www.noo.org/) provides a single point of contact for wide-ranging authoritative information on data, evaluation, evidence and research related to weight status and its determinants. PHE have developed three standard evaluation frameworks for weight management interventions, physical activity and dietary interventions.  PHE also have a collection of resources on evaluation (CoRE) including three Standard Evaluation Frameworks (SEF); evaluation data collection tool (including details of local interventions); other evaluation guidance; reports from evaluation of nationally-initiated schemes; and evaluation websites. This document builds on the ‘Standard Evaluation Framework (SEF) for weight management interventions’, published in April 2009. It takes the principles described in the original SEF and applies them to dietary interventions. |
| Summary / overview | The aim of the Standard Evaluation Frameworks (SEF) is to support high quality, consistent evaluation of weight management, diet and physical activity interventions in order to increase the evidence base.  The guidance document starts with a section entitled “An introduction to evaluation.” This section starts with a discussion of why evaluations are necessary, moving on to how to specify research questions, aims and objectives. Definitions of types of evaluations (i.e. process, formative, outcome), and evaluation designs (i.e., experimental, quasi experimental) are discussed. The document then reviews choosing and measuring outcomes (including logic modelling), methods of data collection, analysis and reporting, managing budgets and ethical issues. General principles (dos and don’ts) and a step by step guide are then provided.  The second section of the document is the framework. This includes 58 essential and desirable criteria that should be collected whilst evaluating weight management interventions. Essential criteria are presented as the minimum recommended data for evaluating a weight management intervention. Desirable criteria are additional data that would enhance the evaluation. For example, the framework states that it is essential to have data on factors including the primary and secondary aims and objectives, intervention timescales, and descriptions of the intervention. However, theoretical underpinnings, and training needs are only desirable. The 58 criteria relate to each stage of the evaluation – from title/name of the intervention to analysis and interpretation. Explanatory notes are accessed by clicking on the links in the interactive document. |
| Strengths | - Clear and comprehensive tool which is accompanied by explanatory notes - Signposted for readers with different levels of knowledge - Aims to improve the standard of evaluation in this area. - Aims to improve generalisability of results - PHE will be evaluating the usefulness and impact of the SEF and has asked readers for feedback - Includes a consideration of cost of intervention per outcome which is often ignored - The framework provides evaluation support to those in the field of weight loss. It is intended to increase the number and quality of evaluations of such programs. - The framework may also contribute to the development of a core dataset that would increase the comparability of evaluations. - The framework provides a basic overview of evaluation to ensure users have a minimum understanding prior to using the framework. - The framework provides a useful checklist that distinguishes between criteria that are essential, and those that are only desirable. Thus, enabling practitioners to prioritise when resources are scares. - The SEF can be used with a range of weight management interventions; including individual, group, or community based interventions. - A wide range of stakeholders including practitioners and academics were heavily involved in the development. - The framework is currently undergoing an evaluation to assess it utility. |
| Limitations | - UK focused - Obesity focused - Checklist approaches may be too simplistic for the nuances of complex interventions. Individual situations may require modifications to the essential and desirable criteria. - The framework is specific only to interventions for weight management. - The document offers a very basic overview of evaluation. This may not be sufficient for practitioners to conduct high quality evaluations without additional help. |
| Link to the resources | <http://www.noo.org.uk/core/frameworks/SEF> |
| Links to related resources | <http://www.noo.org.uk/core>  <http://www.noo.org.uk/core/frameworks/SEF_PA>  <http://www.noo.org.uk/core/frameworks/SEF_Diet> |
| Additional comments |  |

| Title | Monitoring and Evaluation: Some Tools, Methods and Approaches |
| --- | --- |
| Short title | The World Bank |
| Type of document/ accessibility | Online PDF |
| Length | 26 pages |
| Lead author / organisation | The World Bank |
| Date | 2004 |
| Source | The World Bank |
| Purpose and utility of guidance | To strengthen awareness of monitoring and evaluation |
| Primary target audience | Government officials, development managers, and civil society. |
| Contextual information | The world bank’s official goal is to reduce poverty by providing financial assistance; and by supporting developing countries through policy advice, research analysis, and technical assistance. |
| Summary / overview | This document is an overview of a sample of tools, methods and approaches. A series of page long summaries presents the purpose and use, advantages and disadvantages, costs, skills, and time required to use them.  Topics include:   - Performance indicators - The logical framework approach - Theory based evaluation - Formal surveys - Rapid appraisal methods - Participatory methods - Public expenditure tracking surveys - Cost-benefit and cost-effectiveness analysis - Impact evaluation   It is noted that this list is not comprehensive, or intended to be. |
| Strengths | - Available in several languages - Provides a good overview of key monitoring and evaluation tools, methods and approaches, along with information about where to find further information - Useful consideration of the purpose and use, advantages and disadvantages, costs, skills, and time required to use the different approaches. - Includes cost effectiveness - Includes expenditure tracking to outcomes - Discusses cost of evaluation   Refers to other World Bank Evaluation sites |
| Limitations | - Does not provide information on how to monitor or evaluate a program - Title is vague - Helpful to explain what the world bank does at the outset - Helpful to state who the target audience are - Should have placed this within the context of other monitoring and evaluation resources within International development – for example DFIDF, WHO, UN. - Why are certain tools chosen and where would the reader get advice on other tools? - Not clear what the rationale is behind this document. - Closer to a glossary than a handbook - Does not give stakeholder engagement the significance it requires. |
| Link to the resources | <http://lnweb90.worldbank.org/oed/oeddoclib.nsf/24cc3bb1f94ae11c85256808006a0046/a5efbb5d776b67d285256b1e0079c9a3/$FILE/MandE_tools_methods_approaches.pdf> |
| Links to related resources |  |
| Additional comments |  |

| Title | Program Evaluation Methods: Measurement and Attribution of Program Results |
| --- | --- |
| Short title | Treasury Board Canada |
| Type of document/ accessibility | Online pdf |
| Length | 152 pages |
| Lead author / organisation | Treasury Board Canada |
| Date |  |
| Source | Treasury Board Canada |
| Purpose and utility of guidance | To help practitioners understand the methodological considerations involved in measuring and assessing program outcomes. It is not meant to take people step by step through evaluation but to help practitioners understand the different methods used and the strengths and weaknesses of each |
| Primary target audience | Practitioners and other interested parties |
| Contextual information | The Treasury Board is a Cabinet committee of the Queen's Privy Council of Canada. The Treasury Board is responsible for accountability and ethics, financial, personnel and administrative management, comptrollership, approving regulations and most Orders-in-Council. |
| Summary / overview | Chapter one is an introduction to evaluation. It discusses why evaluation is important in federal government, describes the phases involved in evaluation process (evaluation assessment/planning; evaluation study; and decision making based on findings and recommendations). It then goes on to discuss evaluation issues (or what evaluation can be used to address) and methods for addressing them. For example, the document suggests that evaluation can be used to assess continued relevance; results, or cost effectiveness. The chapter differentiates between program theory issues (rationale behind programs) and program results (was the program effective). It highlights that both intended and unintended consequences should be considered, and suggests that two major problems need to be considered (i) measurement problems and (ii) attributional problems (can be attribute the results to the program).  Chapter 2 discusses the kinds of conclusions that can be drawn from an evaluation (i.e., were effects “caused” by the program?). It discusses various threats that may arise (measurement issues, attribution issues, feasibility issues, and practical issues) and presents a conceptual framework for developing evaluation strategies. It concludes by suggesting that multiple strategies generate the most credible conclusions.  Chapter 3 discusses evaluation designs. The chapter starts with an overview of the ‘ideal’ design (RCT), quasi-experimental designs, and implicit designs (before and after trials). Advantages and disadvantages of each are presented.  Chapter 4 discusses data collection methods. The chapter describes both qualitative and quantitative approaches, longitudinal and cross sectional approaches, subjective and objective data, and primary and secondary data. It also discusses 6 common data collection methods (literature review, file review, natural observation, surveying, expert opinion, and case studies).  Chapter five provides information on analytical methods. This includes statistical analysis (descriptive and for inference), qualitative analysis, modelling and cost effectiveness. |
| Strengths | - Clearly written and easy to navigate - Describes research methods needed for evaluation. - Goes beyond a superficial or cookbook approach to evaluation Emphasises the need for consulting people with experience in research and evaluation and gives further sources - Describes the strengths and weaknesses of each method included - Each chapter contains a summary and references to further information. - Provides a useful introduction to understanding evaluation – what can be learnt from it, and different strategies and approaches. - Discusses issues surrounding validity and quality of trials - Discusses data analysis issues - Includes cost benefit analyses - Good emphasis and explanation of causality |
| Limitations | - Undated - Many of the references are over thirty years old and may be dated In terms of evaluation theory and practice - Does not describe modern evaluation theory or approaches such as stakeholder engagement, logic model and so on. - Seems dated- for example with the emphasis on the research focused approach. - Little information on qualitative approaches - The focus is more on understanding evaluation, rather than doing evaluation |
| Link to the resources | <http://www.tbs-sct.gc.ca/cee/pubs/meth/pem-mep-eng.pdf> |
| Links to related resources |  |
| Additional comments | Evaluation studies identified in this search seem to take a research perspective if they are older and a project management one of they are newer. |

| Title | UK evaluation society |
| --- | --- |
| Short title | UKES |
| Type of document/ accessibility | Online web page |
| Length | Not applicable |
| Lead author / organisation | UK evaluation society |
| Date | Ongoing updates |
| Source | Web |
| Purpose and utility of guidance | The site is a membership forum for those working in evaluation. It has generated guidelines to help commissioners, practitioners and participants establish good practice in the conduct of evaluation. These guidelines assimilate a diverse set of principles for action in evaluation and are intended for use in any domain, discipline or context. They outline issues for consideration by a range of stakeholders involved in the evaluation process. In this way it is intended that practice is encapsulated from the point of view of evaluators themselves, commissioners, participants and those involved in self-evaluation in organisations. |
| Primary target audience | Commissioners, practitioners and participants working in evaluation |
| Contextual information | This is the web page for members of the UK evaluation society which is for professional evaluators in the UK. This organisation exists to  promote and improve the theory, practice, understanding and utilisation of evaluation and its contribution to public knowledge.  It was founded in 1994 and members include evaluation professionals, practitioners and evaluation commissioners from national and local government, the research community, independent consultancies and the voluntary sector. |
| Summary / overview | - Provides information on the society and what it does as well as links to its publications - Publishes a magazine on evaluation two or three times a year - Background on good practice guidelines - Contain evaluation jobs and tenders - Contains information on news, training and conference - Networks in different parts of the UK - Has a forum for opinions   The society provides a document entitled “guidelines for good practice in evaluation” this is a list of recommendations that evaluators and commissioners should adhere to. It is more of a checklist, and does not provide and information on how to achieve each point. |
| Strengths | - Promotes links between evaluators - Members from different professions and in different fields - Guidelines grounded in practice and aimed to evolve - Links to useful information in evaluation |
| Limitations | - UK focused - Although claims to publish guidelines that are jargon free, the description of these is written in very complex, jargon style – would benefit from being written in plain English - Could be clearer about what it is for - Not really about evaluation per se but a membership body for those working in evaluation |
| Link to the resources | <http://www.evaluation.org.uk/about-us/publications> |
| Links to related resources |  |
| Additional comments |  |

| Title | Norms for Evaluation in the UN system |
| --- | --- |
| Short title | UNEG |
| Type of document/ accessibility | Online pdf |
| Length | 11 pages |
| Lead author / organisation | United Nation Evaluation Group |
| Date | April 2005 |
| Source | Online |
| Purpose and utility of guidance |  |
| Primary target audience | Those working in any aspect of evaluation within the UN system |
| Contextual information | The UN is an intergovernmental organization to promote international co-operation particularly in the area of such as global governance, consensus building, peace and security, justice and international law, non-discrimination and gender equity, sustained socio-economic development, sustainable development, fair trade, humanitarian action and crime prevention.  The United Nations Evaluation Group (UNEG), are a group of professional practitioners, who wished to define norms that contribute to the professionalization of evaluation and provide guidance to evaluation offices in their work |
| Summary / overview | The authors of this document wish to define norms for evaluation in the UN by facilitating system-wide collaboration on evaluation and by ensuring that evaluation which happens within the UN follow agree-upon basic principles in this way evaluation will be strengthened.  It sets out –   - Definition: How evaluation is defined and how it differs from similar activities such as appraisal. - Responsible: Which bodies are responsible for evaluation in the UN and how this is governed - Policy: What each organisation should have in their evaluation policy - Intentionality - Impartiality: A clear intention to use the findings and evaluations carried out at the right time. Topics should be selected purposively - Independence: Lack of bias and appropriate stakeholder involvement. Evaluator should be independent of policy making - Evaluability: Clear intent and sufficient material to contribute towards the evaluation - Quality of Evaluation: Designed correctly and reported on clearly and consistently - Competencies for Evaluation: Those carrying it out should have the correct skills - Transparency and Consultation: Consultation with appropriate people and results open to public - Evaluation Ethic: Integrity sensitivity to gender and race - Follow up to Evaluation: Commitment to follow up on results - Contribution to Knowledge: Building lessons learned and accessible to correct people |
| Strengths | - Having norms for UN evaluation helps consistency and comparison - No information on economic effectiveness - Clear about what it is and how it can be used - Good guide to what should be in place before evaluation begins in any organisation |
| Limitations | - Could be usefully rewritten in plain English as very complex language - Not a guide to evaluation |
| Link to the resources | [**http://www.unicef.org/evaluation/files/UNEG_Norms_2005-FINAL.pdf**](http://www.unicef.org/evaluation/files/UNEG_Norms_2005-FINAL.pdf) |
| Links to related resources |  |
| Additional comments |  |

| Title | Handbook on Planning, Monitoring and Evaluating for Development Results |
| --- | --- |
| Short title | UNDP |
| Type of document/ accessibility | Online html and can be downloaded as pdf |
| Length | 232 pages |
| Lead author / organisation | United Nations Development Programme (UNDP) |
| Date | 2009 (update of 2002 guide) |
| Source | Online |
| Purpose and utility of guidance | To strengthen a culture of evaluation |
| Primary target audience | Those working in UN development programmes or in other development work who will be involved in evaluation but do not necessarily have evaluation experience or knowledge. |
| Contextual information | The UNDP are active in 166 countries around the world. Their remit is to support national capacity development for poverty reduction and the attainment of the Millennium Development Goals |
| Summary / overview | This seeks to address new directions in planning, monitoring and evaluation within the context of the United Nations Development Programme (UNDP) and evaluation within the UN system.  It focuses on enhancing the results based culture of UNDP and improve the quality of planning, monitoring and evaluation. It provides a basic understanding of this work within the UNDP context and knowledge to carry out effective evaluation and monitoring. It includes: stakeholder engagement, planning, the results framework, planning for change, resources and capacity required, monitoring UNDP policy, dealing with data, evaluation policy and different methods of evaluation and how to learn, generate and disseminate knowledge from evaluation.   - Chapter one describes the purpose of planning, monitoring and evaluation, and provides key definitions and principles that are integral to planning, monitoring, and evaluation (including ownership, engagement of stakeholders, focus on results, and focus on development). - Chapter two provides step-by-step guidance on how to undertake planning for results. It suggests 8 key deliverables should be produced from the planning stage (outline of activities and schedule and cost; Stakeholder influence and importance matrix; List of key problems identified; Prioritized list of problems; Cause-effect diagram or problem tree analysis for each prioritized problems; Vision statement for each prioritized problem; Results map for each prioritized problem; Results framework for the programme or project document) - Chapter three is about planning for monitoring and evaluation. This concerns setting up the systems and processes necessary to ensure the intended results are achieved as planned. The chapter provides guidance on the planning and preparations for effective monitoring and evaluation of such development plans. Plans should include: what is to be monitored and evaluated; the activities needed to monitor and evaluate; who is responsible for monitoring and evaluation activities; when monitoring and evaluation activities are planned (timing); how monitoring and evaluation are carried out (methods); what resources are required and where they are committed. - Chapter 4 provides a detailed step by step guide on how to implement monitoring. - Chapter 5 describes why evaluation is important for UNDP and how evaluative information should be used, then briefly presents the UNDP evaluation policy, types of evaluations that are commonly conducted in UNDP, key roles and responsibilities in evaluation and evaluation requirements as stipulated in the evaluation policy. - Chapter 6 describes the steps involved in preparing for and managing an evaluation. Steps include initiating the evaluation process, preparing, managing the process, and using the evaluation. - Chapter 7 is about ensuring quality. - Finally, chapter 8 is about enhancing the use of knowledge from monitoring and evaluation. - The appendix has a series of practical templates to support evaluation.   Further support on a number of issues are provided in companion documents: Guidance on outcome level evaluation: <http://web.undp.org/evaluation/documents/guidance/UNDP_Guidance_on_Outcome-Level%20_Evaluation_2011.pdf>  Guidance for conducting terminal evaluations of UNDP- supported Global Environment Facility financed projects:  <http://web.undp.org/evaluation/documents/guidance/UNDP_Guidance_on_Outcome-Level%20_Evaluation_2011.pdf>  Impact evaluation:  <http://www.unevaluation.org/document/detail/1433>  These are shorter documents in which further detail is provided on these areas. |
| Strengths | - Available in several languages - Really good overview of evaluation in development work which takes readers through the programme cycle of evaluation. - Based on consultation with experts and stakeholder including a series of workshops - Useful annexes which support the evaluation process in a step by step way. - Emphasises ownership of evaluation methods by those who are working in the programme. - Includes some in country examples. - Provides a good summary of different types of evaluation |
| Limitations | - Repeats much of what can be found elsewhere - No reference to economic evaluation or cost effectiveness |
| Link to the resources | <http://web.undp.org/evaluation/handbook/foreword.html> |
| Links to related resources | \|  \|  \|  \|  \|  \|  \|  \|  \|  \|  \|  \| \| --- \| --- \| --- \| --- \| --- \| --- \| --- \| --- \| --- \| --- \| --- \|   <http://www.undp.org/content/undp/en/home/mdgoverview.html> |
| Additional comments | States that two compendiums should accompany this handbook (1)  planning, monitoring and evaluation in conflict prevention and recovery settings and (2) Guidelines on Outcome Evaluations (under development). These are available on the Evaluation Office website at www.undp.org/eo/handbook. |

| Title | Virtual knowledge centre to end violence against women and girls: Monitoring and Evaluation |
| --- | --- |
| Short title | UNW |
| Type of document/ accessibility | Online |
| Length | Not applicable |
| Lead author / organisation | United Nations |
| Date | 2012 |
| Source | United Nations |
| Purpose and utility of guidance | The website as a whole provides information and guidance for programming to address violence against women and girls. A module on monitoring and evaluation is included. |
| Primary target audience | Those working world wide in any area related to ending violence on women and girls, including those working in the field as well as researchers and policy makers and those who are victims of such violence. |
| Contextual information | The United Nations is an organisation which was formed after the second world war to maintain international peace and security. The primary aim of the site is to encourage and support evidence-based programming to more efficiently and effectively design, implement, monitor and evaluate initiatives to prevent and respond to violence against women and girls  The website contains:   - step by step programme guidance for those working in campaigns related to ending violence against women and girls - Contains statistics on gender violence throughout the world. - It has detailed resources for implementation including how to carry out a campaign, what educational materials to provide, and different approaches that can be used. - It provides specific information about events and training sessions and further help. - All of these aim to support those who are managing, monitoring and evaluating campaigns in this area.   It also provides sources of support throughout the world for those who are victims of gendered violence |
| Summary / Overview | A specific module on monitoring and evaluation provides information on:   - What monitoring and evaluation is, why it is important and challenges that are likely to be faced - How to prepare for monitoring and evaluation; including needs assessments, frameworks (conceptual and logic), choosing indicators, and monitoring and evaluation plans. - Conducting monitoring and evaluation; what questions to ask, how to conduct baseline assessments (qualitative and quantitative), and monitoring, outcome and impact - Monitoring and evaluation for specific areas of work (health, justice, community mobilisation, conflict/post conflict and emergency) |
| Strengths | - Clear and practical step by step guide to the key processes involved in planning and conducting an evaluation - World-wide focused - Available in English, Spanish and French - Easy to navigate - Links are provided throughout to additional material |
| Limitations | - It does not set out what it is or who the target audience are. - Would be helpful if the material was also available as a pdf. - Specifically aimed at those working in gendered violence. - Does not include economic evaluation |
| Link to the resources | http://www.endvawnow.org/en/modules/view/14-programming-essentials-monitoring-evaluation.html%20-%2014#11 |
| Links to related resources |  |
| Additional comments | Has a section on monitoring and evaluation but that is not the primary purpose of this document |

| Title | Global Health Evidence Evaluation Framework: Research White Paper |
| --- | --- |
| Short title | AHRQ |
| Type of document/ accessibility | Online Pdf |
| Length | 149 pages |
| Lead author / organisation | Agency for Healthcare Research and Quality (AHRQ) (prepared by the Southern Californian Evidence Based Practice Centre under contract to AHRQ). Part of an USA International Development Programme (USAID) |
| Date | January 2013 |
| Source | AHRQ |
| Purpose and utility of guidance | To improve the quality of healthcare by helping decision makers to make well informed decisions in conjunction with clinical judgment and other information on resources and patients circumstances. This document may be used as the basis for development of clinical guidelines etc. and reimbursement and coverage policy.  This project aimed to develop an evidence framework in order to inform global health policy |
| Primary target audience | Health care decision makers—patients and clinicians, health system leaders, and policymakers |
| Contextual information | The AHRQ are a US agency which sponsors the development of evidence reports and technology assessments to improve the quality of health care in the United States.  These provide organizations with comprehensive, science-based information on common, costly medical conditions and new health care technologies and strategies  AHRQ expects that the EPC evidence reports and technology assessments will inform individual health plans, providers, and purchasers as well as the health care system as a whole by providing important information to help improve health care quality. The reports undergo peer review prior to their release as a final report. |
| Summary / overview | An extensive literature search of published and grey literature was carried out with the input from a multidisciplinary Technical Expert Panel (TEP).  This identified six existing evidence frameworks for public health/global health interventions and applied these frameworks to the evidence bases for three exemplar interventions: household water chlorination, prevention of mother-to-child transmission of HIV, and lay community health workers to reduce child mortality.   - The findings identified that there was a gap in reporting information once an intervention had been implemented. The authors then identified ten criteria that covered areas identified by the expert panel. These were then tested on three published articles for effectiveness on each of the three exemplar interventions. - The results showed that assessment of the same evidence could give different results regarding the strength of that evidence depending on which framework was used. All frameworks focused on efficacy and or effectiveness and often focused on allocation methods of study participants rather than other methods of study quality such as implementation. None explicitly assessed costs or sustainability. Incorporating insights from other frameworks helped to address these gaps. This made it difficult for policy makers to make judgment about the sensitivity of effectiveness to differences in context, and the scalability and sustainability of the intervention. . - The authors concluded that existing frameworks for the assessment of public health evidence do not deliver key pieces of information to inform best practices for community and large-scale global health programs. In particular there is a lack of information on implementation and sustainability. |
| Strengths | - Structured as a short research report with references and annexes for further information. - States clearly the purpose of the organisation and of this work - Clear, logical and thorough. - Comprehensive search and use of expert panel, |
| Limitations | - Aimed at an academic / clinical audience and not for helping people to carry out evaluation - Specifically for global health policy although it might be useful in other settings. |
| Link to the resources | http://www.ncbi.nlm.nih.gov/books/NBK121291/pdf/TOC.pdf |
| Links to related resources | www.effectivehealthcare.ahrq.gov. |
| Additional comments | This is of a different nature to most of the studies included in this search as it aims to develop a framework rather than support people carrying out evaluations. It evaluated existing frameworks and assessed their strengths and limitations  Might be useful to read in conjunction with other literature in the field of international development found in this search |

| Title | Guide to Analysing the Cost-Effectiveness of community public health prevention approaches | |
| --- | --- | --- |
| Short title | USDHH1 Cost Effectiveness | |
| Type of document/ accessibility | Online PDF | |
| Length | 94 | |
| Lead author / organisation | Amanda A. Honeycutt, Ph.D.  Laurel Clayton, B.A.  Olga Khavjou, M.A.  Eric A. Finkelstein, Ph.D.  Malavika Prabhu, B.S.  Jonathan L. Blitstein, Ph.D.  W. Douglas Evans, Ph.D.  Jeanette M. Renaud, Ph.D. | |
| Date | 2006 | |
| Source | Office of the Assistant Secretary for Planning and Evaluation  U.S. Department of Health and Human Services | |
| Purpose and utility of guidance | To support program managers and evaluators understand, design, and perform cost effectiveness evaluations of community based public health initiatives. | |
| Primary target audience | Program managers and evaluators of community based public health programs. | |
| Contextual information | The Assistant Secretary for Planning and Evaluation (ASPE) advises the Secretary of the Department of Health and Human Services on policy development in many areas including health, and provides advice and analysis on economic policy. The ASPE is tasked with coordinating the Department's evaluation, research, and demonstration activities. The ASPE conducts research and evaluation studies; develops policy analyses; and estimates the cost and benefits of policy alternatives under consideration by the Department or Congress. | |
| Summary / overview | The guide is intended to be used to support the conduct of cost effectiveness analyses. It focuses on the main questions and issues that arise – and provides a list of resources for anyone who requires further guidance or depth of information.  Chapter 2 discusses planning for a cost effectiveness study. It covers all activities from defining the study question, determining the time frame and intervention, and selecting the type of economic study to conduct (i.e., cost analysis, cost effectiveness, cost benefit).  Chapter 3 is about identifying and measuring outcomes. It begins with an overview of existing large scale community programs, and provides advice on how to select outcomes of such programs.  Chapter 4 is about identifying and quantifying program costs.  Chapter 5 outlines the process involved in conducting a cost effectiveness study (with examples).  Chapter 6 discusses how to use the results of a cost effectiveness analysis. | |
| Strengths | - Simple and straight forward language – making it relatively easy to use - Useful for those new to cost effectiveness analysis - Is presented in a logical way; outlining all key steps involved - Provides useful summaries, checklists, and examples - Provides a resource list for those wishing to extend knowledge further | |
| Limitations | - Fairly simplistic, may require evaluators to use outside resources - Largely intended for the evaluation of community based evaluations in the US – although many of the techniques are applicable outside of this area | |
| Link to the resources | <http://aspe.hhs.gov/sites/default/files/pdf/74686/report.pdf> | |
| Links to related resources |  | |
| Additional comments |  | |
| Title | Guide to Analysing the Cost-Effectiveness of community public health prevention approaches |  |
| Type of document/ accessibility | Online PDF |  |
| Length | 94 |  |
| Lead author / organisation | Amanda A. Honeycutt, Ph.D.  Laurel Clayton, B.A.  Olga Khavjou, M.A.  Eric A. Finkelstein, Ph.D.  Malavika Prabhu, B.S.  Jonathan L. Blitstein, Ph.D.  W. Douglas Evans, Ph.D.  Jeanette M. Renaud, Ph.D. |  |
| Date | 2006 |  |
| Source | Office of the Assistant Secretary for Planning and Evaluation  U.S. Department of Health and Human Services |  |
| Purpose and utility of guidance | To support program managers and evaluators understand, design, and perform cost effectiveness evaluations of community based public health initiatives. |  |
| Primary target audience | Program managers and evaluators of community based public health programs. |  |
| Contextual information | The Assistant Secretary for Planning and Evaluation (ASPE) advises the Secretary of the Department of Health and Human Services on policy development in many areas including health, and provides advice and analysis on economic policy. The ASPE is tasked with coordinating the Department's evaluation, research, and demonstration activities. The ASPE conducts research and evaluation studies; develops policy analyses; and estimates the cost and benefits of policy alternatives under consideration by the Department or Congress. |  |
| Summary / overview | The guide is intended to be used to support the conduct of cost effectiveness analyses. It focuses on the main questions and issues that arise – and provides a list of resources for anyone who requires further guidance or depth of information.  Chapter 2 discusses planning for a cost effectiveness study. It covers all activities from defining the study question, determining the time frame and intervention, and selecting the type of economic study to conduct (i.e., cost analysis, cost effectiveness, cost benefit).  Chapter 3 is about identifying and measuring outcomes. It begins with an overview of existing large scale community programs, and provides advice on how to select outcomes of such programs.  Chapter 4 is about identifying and quantifying program costs.  Chapter 5 outlines the process involved in conducting a cost effectiveness study (with examples).  Chapter 6 discusses how to use the results of a cost effectiveness analysis. |  |
| Strengths | - Simple and straight forward language – making it relatively easy to use - Useful for those new to cost effectiveness analysis - Is presented in a logical way; outlining all key steps involved - Provides useful summaries, checklists, and examples - Provides a resource list for those wishing to extend knowledge further |  |
| Limitations | - Fairly simplistic, may require evaluators to use outside resources - Largely intended for the evaluation of community based evaluations in the US – although many of the techniques are applicable outside of this area |  |
| Link to the resources | <http://aspe.hhs.gov/sites/default/files/pdf/74686/report.pdf> |  |
| Links to related resources |  |  |
| Additional comments |  |  |

| Title | The Program Manager’s guide to Evaluation: Second Edition |
| --- | --- |
| Short title | USDHH2 Evaluation |
| Type of document/ accessibility | Online Pdf |
| Length | 122 pages |
| Lead author / organisation | Office of Planning, Research and Evaluation; Administration for Children and Families; US Department of Health and Human Services |
| Date | 2010 |
| Source | US Department of Health and Human Services |
| Purpose and utility of guidance | To support program managers in planning and implementing a program evaluation. It is not intended to turn people into evaluators but to support them when working with people evaluators externally or internally. |
| Primary target audience | Program managers working in the area of Children, Youth, and Families. However, the manual is generic and can be used for many types of evaluation. |
| Contextual information | The Office of Planning, Research and Evaluation (OPRE) is part of the Administration for Children and Families (ACF) division of the US Department of Health and Human Services.  The OPRE is responsible for performance management within the ACF. The OPRE: conduct of research and policy analysis, develops and oversees research projects, provides guidance and assistance on research and evaluation methods, statistical analysis, policy and program analysis, and dissemination of findings. |
| Summary / overview | The document is designed so that each chapter addresses a specific step in the evaluation process.   - The document starts with an overview of evaluation; what it is, what questions it can answer, what is involved in conducting an evaluation, and what an evaluation will cost. - The two chapters discuss external evaluators; who to choose and how to manage them. - The following chapter discusses preparation for evaluation: deciding what to evaluate, developing a logic model, stating implementation and outcomes in measurable terms, and identifying the context. - The next chapter explains what to include in an evaluation. This contains information on the evaluation framework, evaluating implementation objectives, evaluating outcome objectives, and procedures for managing and monitoring. - Chapter seven discusses how to get the information needed. This includes what specific information is needed to address objectives, what the best sources are, how data could be collected effectively and using what tools, and how data collection can be monitored. - Chapter 8 is about making sense of data, analysing data on both implementation and outcomes, and using the results. - The final chapter provides information on reporting and disseminating findings for different stakeholders.   At the end of the document, a list of evaluation resources, other guidance documents, evaluation consultants, data collection methods, and other tool kits is provided. |
| Strengths | - This guidance assumes no or very little prior knowledge of evaluation - Provides practical advice on conducting real world evaluations, rather than academically rigours evaluations - Provides different and useful information that is not always mentioned in other guides such as the makeup of your evaluation team, how to hire an evaluator, how to create a contract. - Fairly comprehensive in the topic covered - Simple to follow and does not provide too much in-depth of information - It is generic, and so can be used for evaluation of any types of projects (i.e., it is not restricted to a specific type of project or condition) - Considers how to manage external evaluators - A glossary of terms is included - The appendix includes a number of useful templates and frameworks to support evaluation – i.e., templates for evaluation plans - Provides links to other resources - It divides evaluations by cost (low, moderate, high) and gives advice for each. - Has a related documents which gives detailed information on cost analysis - Useful glossary |
| Limitations | - Assumes evaluations will be simplistic - May not provide enough detail to support practitioners to conduct a rigorous evaluation of a program without seeking additional information (e.g., only briefly describes the difference between qualitative and quantitative data, what an RCT is… etc). - US focused - Children and Family focused - Some worked examples would have been helpful. - Many of the references are over thirty years old |
| Link to the resources | <http://www.acf.hhs.gov/sites/default/files/opre/program_managers_guide_to_eval2010.pdf> |
| Links to related resources |  |
| Additional comments |  |

| Title | A Framework for Monitoring and Evaluating HIV Prevention Programmes for Most-At-Risk Populations |
| --- | --- |
| Short title | UNAIDS |
| Type of document/ accessibility | Online Pdf |
| Length | 96 |
| Lead author / organisation | UNAIDS |
| Date | English Original 2008. Reprint 2009 |
| Source | Online |
| Purpose and utility of guidance | To support planning and implementation programmes, monitoring and evaluation, and using data and information for policy development and programme improvement, in work in AIDS in poorer countries. |
| Primary target audience | For national and subnational programme managers and others involved in this work. |
| Contextual information | UNAIDS are the Joint United Nation Programme on HIV and AIDS that leads the world in providing universal access to HIV and AIDS prevention and care. |
| Summary / overview | Existing monitoring and evaluation guides, particularly for prevention programmes, have been developed largely with generalised epidemics in mind. The requirements of monitoring and evaluation in HIV are often different because they need to focus on geographical areas where HIV is concentrated, rather than throughout the country, and on the unique needs of the most at risk populations, rather than the population as a whole. There has been methods and approaches developed for monitoring and evaluation in these populations and much of this has been documented. However they are not in the one place and need to be pulled together to provide a comprehensive review. The data collection for these groups has also been done largely in an ad hoc fashion and this document highlights the importance of subnational and project level monitoring and evaluation in order to target the most at risk populations.  This document is first step in this process and aims to lay out key guiding principles, concepts, and an organizing framework to help work in this area develop further. It also aims to bring the different methods, references and materials published separately, together into one document. It highlights the need for analytical skills for carrying out this work.  In order to investigate a problem one would start with defining what the problem is, exploring contributing factors, analysing what can be done about the problem and then when this has been put into place checking to see if it is working, what the reach is and what affect it has. This document includes methods of population size estimations in order to support the identification of the problem and the situation in which it takes place, discusses assessing the contribution factors, presents monitoring and evaluation process, discusses methods to track programme uptake and coverage, describes how to assess whether an intervention is effective using outcome evaluation studies, discusses monitoring outcome and impact indicators and the role of surveillance and covers assessing collective effectiveness through triangulation methods.  It emphasises ethics, the need to involve stakeholders including the community and population under study and the need to be sensitive to cultural norms.  It states that it is not meant to be a step by step guide to carrying out evaluation |
| Strengths | - Intended for use World Wide and includes worldwide partners. - Wide consultation during development - Principles relevant to other health topics. - The need for appropriate research skills emphasised - Illustrates theory with examples of work in the field. - Overview of main issues associated with monitoring and evaluation. - Clear about what it is and what it is not |
| Limitations | - Specific focus on AIDS - An overview and therefore not intended to be used as a manual to support carrying out evaluation. - Does not discuss theoretical issues in evaluation - Does not include cost effectiveness, resource allocation or any reference to economic evaluation - Would need to be adapted for use in different settings |
| Link to the resources | http://www.unaids.org/sites/default/files/en/media/unaids/contentassets/documents/document/2010/17_Framework_ME_Prevention_Prog_MARP_E.pdf |
| Links to related resources | http://www.who.int/hiv/pub/guidelines/en |
| Additional comments | Will be tested in the field and further practical and operational guidance developed.  This operational guidance will include differences in approaches depending on context, country, group and so on.  Includes the indicators for the most at risk populations in the annex. |

| Title | W.K. Kellogg Foundation Evaluation Handbook |
| --- | --- |
| Short title | W.K.Kellogg |
| Type of document/ accessibility | Online PDF |
| Length | 120 pages |
| Lead author / organisation | W.K Kellogg |
| Date | 2004 |
| Source | W.K. Kellogg |
| Purpose and utility of guidance | The handbook provides a framework for thinking about evaluation as a relevant and useful program tool and to support those organisations which are the recipients of Kellogg Foundation. Its principles are that evaluation should be used to show the effectiveness of one method of intervention over another not just in terms of outcomes but in terms of understanding the processes that lead to these outcomes and looking at change for the individual. It should be supportive and responsive to projects, rather than become an end in itself. |
| Primary target audience | The handbook is written primarily for project directors who have direct responsibility for the ongoing evaluation of W.K. Kellogg Foundation-funded projects. However, it may also be used by other project staff who have evaluation responsibilities, for external evaluators, and for board members. |
| Contextual information | WKKF Boilerplate: The W.K. Kellogg Foundation (WKKF), founded in 1930 as an independent, private foundation by breakfast cereal pioneer, Will Keith Kellogg, is among the largest philanthropic foundations in the United States. Guided by the belief that all children should have an equal opportunity to thrive, WKKF works with communities to create conditions for vulnerable children so they can realize their full potential in school, work and life.  The Kellogg Foundation is based in Battle Creek, Michigan, and works throughout the United States and internationally, as well as with sovereign tribes. Special emphasis is paid to priority places where there are high concentrations of poverty and where children face significant barriers to success. WKKF priority places in the U.S. are in Michigan, Mississippi, New Mexico and New Orleans; and internationally, are in Mexico and Haiti. For more information, visit [www.wkkf.org](http://www.wkkf.org/).  The guidance document starts with an overview of its underlying philosophy – critiquing the dominant “scientific method,” and suggesting that, in human service evaluation, many complicated factors are at play that should be considered.  The foundation suggest that evaluation should:   - Strengthen projects; - Use multiple approaches; - address real issues (based on local circumstances and issues); - Create a participatory process; - Allow for flexibility; and - Build capacity. |
| Summary / overview | The handbook is in two parts and serves as a framework for grant recipients both to have a shared vision for effective evaluation and to have a blueprint for designing and conducting evaluation.  Part 1- overview of evaluation including a summary of the most important characteristics of the Foundation’s evaluation approach, the contextual factors that have led to a focus on proving services work rather than improving them, and an overview of the Foundation’s three levels of evaluation (project, cluster and program / policy making) with a particular focus on project-level.  It sets out the negative consequences of ineffective evaluation. (1) belief that there is only one way to do evaluation, (2) do not examine equally important questions – not just that it works but for whom and in what circumstances (3) difficult when evaluation complex systems (4) lose sight that evaluation is political and value laden. It emphasises that if evaluation is ineffective then important projects will not get the money they need as they will not convince of their worth and that knowledge will be lost.  Part 2- provides a description of the three components of project-level evaluation – the context, the implementation and the outcome and also provides logic model examples to show how these link. It then takes readers through each of the steps to carry out an evaluation: preparing, identifying stakeholders and establishing a team, developing the questions, budget, selecting an evaluator, designing and conducting evaluation, choosing data collection methods, collecting data, analysing and interpreting, communicating findings and using the results. |
| Strengths | - Really good insight into the philosophy and history of evaluation and how this influences how it is carried out which is not often in evaluation guides. - Discusses the importance of evaluation for using resources most efficiently. - Committed to improving programs rather than proving that a program works or doesn’t work - Promotes community/stakeholder involvement - Discusses epistemological underpinnings - Differentiates between types of evaluation (context, implementation, and outcome) - Provides detailed consideration of the nine steps that are considered to be crucial for evaluation - Uses real case studies from the foundation to demonstrate issues - Includes cost of evaluation - Links project evaluation to project outcome and shows how if evaluation is built in at the beginning it will improve the project - Emphasises the policy context and the importance of placing evaluation in this. - Encourages people to influence policy from evaluation results. - Emphasises the importance of improvement for the individual rather than just the project and alongside this, the need to include different stakeholders so that one perspective is not favoured. - Emphasises how projects can build individual’s capacity (both staff and clients) and that this should be reflected in the evaluation. - Discusses the concept of perspective and how an improvement from a government’s perspective would be different from that of an individual’s perspective and how to factor this into indicators and outcomes. - States that evaluations should be reflexive, flexible and participatory. - Shows that evaluation is not value free, - Provides worksheets to support programme evaluation |
| Limitations | - May provide too much detail for someone looking for a general overview (i.e., epistemological underpinnings) - Focuses largely on complex evaluations of community projects (as opposed to relatively simple impact evaluations). - Assumes a large budget/multiple resources will be available - US focused |
| Link to the resources | <http://www.wkkf.org/resource-directory/resource/2010/w-k-kellogg-foundation-evaluation-handbook> |
| Links to related resources |  |
| Additional comments | Kellogg also feel that this can be used by smaller projects too. |

| Title | Making Choices in Health: WHO Guide to Cost Effectiveness Analysis |
| --- | --- |
| Short title | WHO1 |
| Type of document/ accessibility | Online PDF |
| Length | 329 |
| Lead author / organisation | World Health Organisation |
| Date | 2003 |
| Source | WHO |
| Purpose and utility of guidance | For researchers and policy makers to compare the cost effectiveness of new interventions compared to that of current interventions and to support them in generalising this to other interventions in order to reduce the costs of such cost-effectiveness analysis. Intended to be complementary to existing guidelines on cost effectiveness analysis |
| Primary target audience | The intended audience is policy makers and researchers working in this area. (There is an assumption that these people have some knowledge of economics). |
| Contextual information | WHO is the authority for health within the United Nations system. It is responsible for providing leadership on global health matters, shaping the health research agenda, setting norms and standards, articulating evidence-based policy options, providing technical support to countries and monitoring and assessing health trends. |
| Summary / overview | The problem of traditional (incremental) Cost Effectiveness Analysis (CEA) is that it compares new interventions to current interventions but does not address whether the current mix of interventions are themselves an efficient use of resources. It does not address the assumption that they additional resources required would need to come from another sector. In addition it is expensive to carry out Cost Effective Analyses when there are a large number of interventions, which means that poorer countries are often not able to do this. Cost effectiveness analyses should also consider the whole system, that is where other costs are incurred by society, how it affects different social groups, ethical issues and effects on future generations but this is rarely done. Therefore this guide aims to show how to assess whether the current mixture of interventions is efficient, how it compares to a proposed measure and how to maximise the generalizability across settings. In the annex it also reproduces papers published elsewhere which provide background to the technical issues discussed. The chapters are:  1. What is Generalized Cost-Effectiveness Analysis?  2. Overall study design  3. Estimating costs  4. Estimating health effects  5. Discounting  6. Uncertainty in cost-effectiveness analysis  7. Policy uses of Generalized CEA  8. Reporting CEA results  9. Recommendations  The second part of the guide includes papers published elsewhere which consider these issues and the annex includes papers where these methods have been used in practice and the ethical issues in this area. |
| Strengths | - Credible organisation - Comprehensive guide on cost effective analyses which supports policy makers in making choices of health interventions. - Supports assessment of efficiency of current system and recommends that costs are considered against ‘the null’ rather than the current system. - Shows how to generalise findings which reduces resources spent on this analyses and allows poorer counties to use findings in their own settings. - Looks at the wider health and societal system – that is it considers issues of equality, ethics, discounting, external factors for health and the effects on future generations. - Includes background papers which discusses aspects of this analysis in more detail. - Clear useful recommendations which can stand alone. |
| Limitations |  |
| Link to the resources | <http://www.webcitation.org/query.php?url=http://www.who.int/choice/publications/p_2003_generalised_cea.pdf&refdoi=10.1186/1471-2458-13-1001> |
| Links to related resources |  |
| Additional comments |  |

| Title | WHO Evaluation Practice Handbook |
| --- | --- |
| Short title | WHO2 |
| Type of document/ accessibility | Online pdf |
| Length | 161 |
| Lead author / organisation | WHO |
| Date | 2013 |
| Source | World Health Organisation |
| Purpose and utility of guidance | Offers comprehensive information and practical guidance on how to prepare for and conduct evaluations in WHO, and guidance on using and following up the results and recommendations. Specifically aimed at those working with and for WHO but could be used by anyone involved in evaluation. |
| Primary target audience | All WHO staff and partner organisations who plan, manage, conduct or are involved in WHO programmes and evaluations.  It also targets networks such as WHO’s senior management, and the Global Network Evaluation (GNE) who should disseminate this and promote evaluation throughout the organisation. |
| Contextual information | WHO is the authority for health within the United Nations system. It is responsible for providing leadership on global health matters, shaping the health research agenda, setting norms and standards, articulating evidence-based policy options, providing technical support to countries and monitoring and assessing health trends. This evaluation guide complements the World Health Organisation’s evaluation policy (set out is in the Annex of this guide). It aims to support streamlining of evaluation and provide step by step support to evaluation in WHO. It is a working tool which will be adapted to support evolving practice. In addition there are plans for an e learning guide to accompany this. |
| Summary / overview | This handbook is in three parts. The first covers the definition, objectives,  principles and management of evaluation in WHO. The second provides practical guidance on preparing for conducting an evaluation in compliance with WHO’s evaluation policy. It goes through this in a step by step process:  from planning, to carrying out to reporting and disseminating findings and ensuring these contribute towards ongoing improvement. The third part includes the annexes which provide further information on the guide and support for evaluation. It also provides operational guidance and templates, |
| Strengths | - Really comprehensive and detailed evaluation guide, including information on what evaluation is as well as detailed support on how to do it. - Useful for evaluation in any context. - Appendices give helpful additional information including roles and responsibilities for evaluation and other types of assessment that are not evaluation. - Aim to develop online tool to support this. - As part of dissemination of findings it emphasises capacity building approaches post evaluation. - Emphasises evaluation as part of ongoing improvement and relates it to future planning and commitment to action. |
| Limitations | - It would have been useful to have seen worked examples of WHO evaluations in practice using this guide, or even concrete examples of where different sections could be used in specific WHO programmes - Support from experienced evaluators would be helpful alongside this document - It does not include evaluation theory. - The information on measuring impact is limited. |
| Link to the resources | http://apps.who.int/iris/bitstream/10665/96311/1/9789241548687_eng.pdf |
| Links to related resources | http://www.uneval.org/document/detail/1160 |
| Additional comments | There are many examples of WHO evaluations on the web which can complement this.  The learning tool designed to accompany this does not seem to be available as yet but it is worth looking out for it. |
