# Supplementary material for: A review of Grey and academic literature of evaluation guidance relevant to public health interventions
Source: BMC Health Serv Res. 2017 Sep 12;17:643. doi: 10.1186/s12913-017-2588-2 (PMC5596848; doi:10.1186/s12913-017-2588-2)
Supplement: Supplementary file 5 — Overarching table listing each of the 48 guides and the key pieces of content contained within them. Covering: Background to evaluation and pre-evaluation preparatory work. Table S2. Overarching table listing each of the 48 guides and the key pieces of content contained within them. Covering: evaluation processes, types of evaluation and additional support. (DOCX 24 kb) [file 12913_2017_2588_MOESM5_ESM.docx]

Table 1: Overarching table listing each of the 48 guides and the key pieces of content contained within them. Covering: Background to evaluation and pre-evaluation preparatory work.

|  | 1 | 2 | 3 | 4 | 5 | 6 | 7 | 8 | 9 | 10 | 11 | 12 | 13 | 14 | 15 | 16 |
| --- | --- | --- | --- | --- | --- | --- | --- | --- | --- | --- | --- | --- | --- | --- | --- | --- |
| 1 |  |  |  |  |  |  |  |  |  | 🗸 |  | 🗸 |  |  | 🗸 | 🗸 |
| 2 |  |  |  |  |  |  |  |  |  |  |  |  |  |  |  |  |
| 3 | 🗸 |  |  |  |  |  |  |  |  |  |  |  | 🗸 |  | 🗸 |  |
| 4 |  |  |  |  |  |  |  |  |  |  |  |  |  |  |  | 🗸 |
| 5 |  |  |  |  |  |  |  |  | 🗸 | 🗸 |  |  |  | 🗸 | 🗸 | 🗸 |
| 6 |  |  |  |  |  |  |  |  |  | 🗸 |  |  |  | 🗸 |  | 🗸 |
| 7 |  |  |  |  |  |  |  |  | 🗸 | 🗸 | 🗸 |  |  | 🗸 |  | 🗸 |
| 8 |  |  |  |  |  |  |  |  |  |  |  |  |  |  |  |  |
| 9 |  |  |  |  |  |  |  |  |  |  |  |  |  |  |  |  |
| 10 |  |  |  |  |  |  |  |  | 🗸 |  |  |  |  | 🗸 | 🗸 |  |
| 11 |  |  |  |  |  |  |  |  |  |  |  |  |  |  |  |  |
| 12 |  |  |  |  |  |  |  |  |  |  |  |  |  |  |  |  |
| 13 | 🗸 | 🗸 | 🗸 |  |  |  |  |  |  |  |  |  |  |  |  |  |
| 14 |  |  |  |  |  |  |  |  | 🗸 |  |  |  |  |  | 🗸 |  |
| 15 | 🗸 |  |  |  |  |  |  |  |  |  |  | 🗸 |  |  |  |  |
| 16 |  |  |  | 🗸 |  |  |  |  |  |  |  |  |  |  |  |  |
| 17 | 🗸 | 🗸 |  |  |  | 🗸 |  |  |  |  |  |  |  | 🗸 |  |  |
| 18 | 🗸 |  |  |  |  |  |  |  |  | 🗸 |  |  |  |  |  | 🗸 |
| 19 |  | 🗸 |  |  |  | 🗸 |  |  |  |  |  |  |  |  |  |  |
| 20 | 🗸 |  |  |  |  |  |  |  |  |  |  |  | 🗸 | 🗸 | 🗸 |  |
| 21 |  |  |  |  |  |  |  |  |  |  |  |  |  |  |  |  |
| 22 |  | 🗸 | 🗸 |  |  |  | 🗸 |  | 🗸 |  | 🗸 |  |  |  | 🗸 |  |
| 23 |  |  |  |  |  |  | 🗸 |  | 🗸 |  |  |  |  | 🗸 | 🗸 |  |
| 24 |  |  |  |  |  |  |  |  | 🗸 |  |  |  |  |  |  |  |
| 25 | 🗸 |  |  |  |  |  | 🗸 |  |  |  |  |  |  | 🗸 | 🗸 | 🗸 |
| 26 | 🗸 | 🗸 |  |  |  |  | 🗸 | 🗸 |  |  |  |  |  | 🗸 |  | 🗸 |
| 27 |  |  |  |  |  |  | 🗸 |  |  | 🗸 |  |  | 🗸 | 🗸 |  | 🗸 |
| 28 | 🗸 | 🗸 | 🗸 |  | 🗸 |  |  |  |  |  |  |  |  |  |  |  |
| 29 |  |  |  |  |  |  |  |  |  |  |  |  |  |  | 🗸 |  |
| 30 |  |  |  |  |  |  |  |  |  |  |  |  |  |  |  |  |
| 31 | 🗸 |  |  |  |  |  |  |  |  |  |  |  |  |  |  |  |
| 32 |  |  |  |  |  |  |  |  |  |  |  |  |  |  |  |  |
| 33 | 🗸 |  |  |  |  |  |  |  |  |  |  |  |  | 🗸 |  |  |
| 34 | 🗸 |  |  |  |  |  |  |  |  |  |  |  |  | 🗸 |  |  |
| 35 | 🗸 |  |  |  |  |  |  |  |  |  |  |  |  | 🗸 |  |  |
| 36 |  |  |  |  |  |  | 🗸 |  |  |  |  |  |  |  | 🗸 |  |
| 37 |  |  |  |  |  |  |  |  |  |  |  |  |  |  |  |  |
| 38 |  |  |  |  |  |  |  |  |  |  |  |  |  |  |  |  |
| 39 |  |  |  |  | 🗸 |  |  |  |  | 🗸 |  | 🗸 |  |  |  |  |
| 40 |  |  |  |  | 🗸 |  |  |  |  | 🗸 |  |  | 🗸 | 🗸 |  | 🗸 |
| 41 | 🗸 |  |  |  | 🗸 |  | 🗸 |  |  |  |  |  | 🗸 | 🗸 | 🗸 |  |
| 42 |  | 🗸 |  |  |  |  |  |  |  |  |  |  |  |  |  |  |
| 43 |  |  |  |  |  |  |  |  | 🗸 |  |  |  |  |  |  |  |
| 44 | 🗸 |  |  |  | 🗸 |  | 🗸 |  | 🗸 | 🗸 |  |  | 🗸 | 🗸 | 🗸 |  |
| 45 |  |  |  |  |  |  |  |  |  |  |  |  | 🗸 |  |  |  |
| 46 | 🗸 |  |  |  |  |  |  |  | 🗸 | 🗸 |  |  |  |  | 🗸 | 🗸 |
| 47 |  |  |  |  |  |  |  |  |  |  |  |  |  |  |  |  |

Key

1. Overview of evaluation
2. Assessing the evidence
3. Evidence based medicine
4. Evaluability
5. Common challenges
6. Policy and evaluation
7. Using theory in evaluation
8. Developing a protocol
9. Budgeting
10. Contracting and communications
11. Pilot testing
12. Ethics
13. Needs assessment
14. Evaluation planning
15. Logic modelling
16. Stakeholder involvement

Table 2: Overarching table listing each of the 48 guides and the key pieces of content contained within them. Covering: evaluation processes, types of evaluation and additional support.

|  | 1 | 2 | 3 | 4 | 5 | 6 | 7 | 8 | 9 | 10 | 11 | 12 | 13 | 14 | 15 | 16 | 17 | 18 | 19 | 20 | 21 | 22 |
| --- | --- | --- | --- | --- | --- | --- | --- | --- | --- | --- | --- | --- | --- | --- | --- | --- | --- | --- | --- | --- | --- | --- |
| 1 |  | 🗸 |  |  |  |  |  |  |  |  |  |  |  |  |  | 🗸 | 🗸 | 🗸 | 🗸 | 🗸 | 🗸 | 🗸 |
| 2 |  |  |  |  |  |  |  |  |  | 🗸 |  |  |  |  |  |  | 🗸 |  | 🗸 |  |  |  |
| 3 |  |  |  |  |  |  |  |  |  |  | 🗸 | 🗸 |  |  |  |  |  |  |  | 🗸 | 🗸 | 🗸 |
| 4 |  |  |  |  |  |  |  |  |  |  |  | 🗸 |  |  | 🗸 |  |  | 🗸 | 🗸 | 🗸 | 🗸 | 🗸 |
| 5 |  |  |  | 🗸 |  |  |  |  |  | 🗸 | 🗸 | 🗸 |  |  | 🗸 | 🗸 | 🗸 | 🗸 | 🗸 | 🗸 |  | 🗸 |
| 6 |  |  |  |  |  |  |  |  |  | 🗸 |  | 🗸 | 🗸 |  | 🗸 | 🗸 |  | 🗸 | 🗸 | 🗸 | 🗸 | 🗸 |
| 7 |  |  |  |  |  |  |  |  |  | 🗸 |  |  |  | 🗸 | 🗸 |  |  |  | 🗸 | 🗸 |  | 🗸 |
| 8 |  | 🗸 |  |  |  |  |  |  |  |  |  |  |  |  | 🗸 |  |  |  |  |  |  |  |
| 9 |  |  |  |  |  |  |  |  |  |  | 🗸 |  |  |  | 🗸 |  |  |  |  |  |  |  |
| 10 |  |  |  |  |  |  |  |  |  |  |  |  |  |  |  |  | 🗸 |  | 🗸 | 🗸 |  |  |
| 11 |  |  |  |  |  |  |  |  |  |  |  |  |  |  |  |  |  |  |  |  |  |  |
| 12 |  |  |  |  |  |  |  |  |  |  |  |  |  |  |  |  |  |  |  |  |  |  |
| 13 |  |  |  |  |  |  |  |  |  | 🗸 |  |  |  |  |  |  |  |  |  |  |  |  |
| 14 |  |  |  |  |  |  |  |  |  |  |  |  | 🗸 | 🗸 |  | 🗸 | 🗸 |  | 🗸 | 🗸 | 🗸 | 🗸 |
| 15 |  | 🗸 |  |  |  |  |  |  |  | 🗸 |  |  |  |  |  |  |  |  | 🗸 | 🗸 | 🗸 | 🗸 |
| 16 |  |  |  |  |  |  |  |  |  |  |  |  |  |  |  |  |  |  |  |  |  |  |
| 17 |  | 🗸 | 🗸 | 🗸 |  |  |  |  |  |  |  |  |  |  | 🗸 |  |  |  | 🗸 | 🗸 |  | 🗸 |
| 18 |  | 🗸 | 🗸 | 🗸 |  |  |  |  |  | 🗸 |  |  |  |  | 🗸 |  |  |  | 🗸 | 🗸 | 🗸 | 🗸 |
| 19 |  |  |  | 🗸 |  |  |  |  |  |  |  |  |  |  |  |  |  |  |  |  |  |  |
| 20 |  |  |  |  |  | 🗸 |  |  |  |  |  |  |  |  | 🗸 | 🗸 | 🗸 | 🗸 |  | 🗸 |  |  |
| 21 |  |  |  |  |  |  |  |  |  |  |  |  |  |  |  |  |  |  |  |  |  |  |
| 22 |  | 🗸 | 🗸 | 🗸 |  |  | 🗸 |  |  |  |  |  |  |  |  | 🗸 | 🗸 | 🗸 | 🗸 |  | 🗸 | 🗸 |
| 23 |  | 🗸 | 🗸 |  |  |  | 🗸 |  |  |  |  |  |  |  |  |  | 🗸 | 🗸 | 🗸 |  | 🗸 |  |
| 24 |  |  |  |  | 🗸 |  |  |  |  |  |  |  |  |  |  |  | 🗸 | 🗸 | 🗸 | 🗸 | 🗸 |  |
| 25 | 🗸 |  |  |  |  |  |  |  |  |  |  |  | 🗸 |  | 🗸 | 🗸 | 🗸 | 🗸 | 🗸 | 🗸 | 🗸 | 🗸 |
| 26 | 🗸 |  |  |  |  |  |  |  |  | 🗸 |  | 🗸 |  | 🗸 | 🗸 | 🗸 |  |  | 🗸 |  |  |  |
| 27 |  |  | 🗸 |  |  | 🗸 |  |  |  |  | 🗸 |  |  |  |  | 🗸 | 🗸 | 🗸 | 🗸 | 🗸 |  |  |
| 28 |  |  |  |  |  |  |  |  |  | 🗸 |  |  |  |  |  |  |  |  |  |  |  |  |
| 29 | 🗸 | 🗸 | 🗸 |  |  |  |  |  |  | 🗸 |  |  |  |  |  |  | 🗸 |  |  |  |  |  |
| 30 |  |  |  |  |  |  |  |  | 🗸 |  |  |  |  |  |  |  |  |  |  |  |  |  |
| 31 |  |  |  |  |  |  |  |  |  |  |  |  |  |  |  |  |  |  |  |  |  |  |
| 32 |  |  |  |  |  |  |  |  |  | 🗸 |  |  |  | 🗸 | 🗸 |  | 🗸 |  |  | 🗸 |  |  |
| 33 |  | 🗸 | 🗸 |  |  |  | 🗸 |  |  | 🗸 |  |  |  |  | 🗸 | 🗸 | 🗸 | 🗸 |  | 🗸 | 🗸 | 🗸 |
| 34 |  | 🗸 | 🗸 |  |  |  | 🗸 |  |  | 🗸 |  |  |  |  | 🗸 | 🗸 | 🗸 | 🗸 |  | 🗸 | 🗸 | 🗸 |
| 35 |  | 🗸 | 🗸 |  |  |  | 🗸 |  |  | 🗸 |  |  |  |  | 🗸 | 🗸 | 🗸 | 🗸 |  | 🗸 | 🗸 | 🗸 |
| 36 |  |  | 🗸 | 🗸 |  |  |  |  |  |  | 🗸 |  |  |  |  |  | 🗸 |  | 🗸 | 🗸 | 🗸 |  |
| 37 |  |  |  |  |  |  |  |  |  | 🗸 |  |  |  |  |  |  |  |  | 🗸 | 🗸 | 🗸 |  |
| 38 |  |  |  |  |  |  |  | 🗸 |  |  |  |  |  |  |  |  |  |  |  |  |  |  |
| 39 |  |  |  |  |  |  |  |  | 🗸 |  |  |  |  | 🗸 |  |  |  |  | 🗸 |  |  |  |
| 40 |  |  | 🗸 |  |  |  |  |  | 🗸 |  |  | 🗸 |  |  | 🗸 | 🗸 | 🗸 | 🗸 | 🗸 | 🗸 | 🗸 | 🗸 |
| 41 |  |  | 🗸 |  |  |  |  |  |  |  |  |  |  |  |  |  | 🗸 | 🗸 | 🗸 | 🗸 | 🗸 | 🗸 |
| 42 |  |  |  |  |  |  |  |  |  |  |  |  |  |  |  |  |  |  |  |  |  |  |
| 43 |  |  |  | 🗸 |  |  |  |  |  |  |  |  |  |  |  | 🗸 | 🗸 | 🗸 | 🗸 | 🗸 | 🗸 | 🗸 |
| 44 |  |  |  |  |  |  |  |  |  | 🗸 |  |  | 🗸 |  | 🗸 | 🗸 | 🗸 | 🗸 | 🗸 | 🗸 | 🗸 | 🗸 |
| 45 |  | 🗸 | 🗸 |  |  |  | 🗸 |  |  |  |  |  |  |  |  |  | 🗸 |  |  |  |  |  |
| 46 | 🗸 | 🗸 | 🗸 |  |  |  | 🗸 |  |  |  |  |  | 🗸 |  | 🗸 |  |  |  | 🗸 | 🗸 | 🗸 | 🗸 |
| 47 |  |  |  | 🗸 |  |  |  |  |  |  |  |  |  |  |  |  |  |  |  |  |  |  |

Key

1. Overview of
2. Process evaluation
3. Outcome evaluation
4. Economic evaluation
5. Evaluating natural experiments
6. Evaluating community projects
7. Fidelity
8. Organisations offering support
9. Recommendations
10. Tools and tool kits
11. Links to additional resources
12. Quality assurance
13. Hiring an evaluator
14. Training
15. Overview of evaluation processes
16. Defining questions
17. Choosing outcomes
18. Describing the intervention
19. Research design and methods
20. Collecting data
21. Managing, analysing and interpreting data
22. Learning and reporting
